# Global Epidemiological Features of Human Monkeypox Cases and Their Associations With Social-Economic Level and International Travel Arrivals: A Systematic Review and Ecological Study

**DOI:** 10.3389/ijph.2023.1605426

**Published:** 2023-01-20

**Authors:** Min Du, Huimin Sun, Shimo Zhang, Jie Yuan, Wenxing Yan, Qiao Liu, Chenyuan Qin, Min Liu, Jue Liu

**Affiliations:** ^1^ Department of Epidemiology and Biostatistics, School of Public Health, Peking University, Beijing, China; ^2^ Global Center for Infectious Disease and Policy Research, Global Health and Infectious Diseases Group, Peking University, Beijing, China; ^3^ Institute for Global Health and Development, Peking University, Beijing, China

**Keywords:** systematic review, epidemiology, disparities, travel, mpox

## Abstract

**Objectives:** We aimed to evaluate global epidemiological features of human monkeypox (mpox) cases and their associations with social-economic level and international travel arrivals.

**Methods:** We estimated the pooled value by random-effects models. Then, we conducted an ecological study to evaluate the relationship of confirmed cases with social-economic indices and international travel arrivals using correlation analyses.

**Results:** The average age (2022: 35.52, 95% CI [28.09, 42.94] vs. before 2022: 18.38, 95% CI [14.74, 22.02]) and comorbidity rate (2022: 15.7%, 95% CI [8.9%, 22.4%] vs. before 2022: 14.9%, 95% CI [8.5%, 21.3%]) of mpox cases in the 2022 human mpox outbreak were significantly higher than those of cases before 2022. During the 2022 mpox outbreak, the proportion of men who have sex with men (MSM) was high (79.8%, 95% CI [65.5%, 94.2%]). The number of confirmed mpox cases in 2022 significantly correlated with high social-economic levels and international travel arrivals (all *p* < 0.05).

**Conclusion:** Our findings highlighted the importance of early surveillance and timely detection in high-risk populations, including older people, MSM, and travelers, which is crucial to curb the wide transmission of mpox.

## Introduction

As of 21 September 2022, human monkeypox (mpox) had spread across 106 countries or territories [1, 2]. Human mpox, a sporadic zoonosis in rural rainforest villages of Western and Central Africa, is caused by two clades of mpox virus, namely, the Central African clade (Clade I) and the West African clade (Clade II) [3, 4]. Human infections have been documented in individuals handling infected monkeys, Gambian giant rats, and squirrels, with rodents being the most likely reservoir of the virus [5]. Mpox virus was first identified in captive monkeys in 1958, and in a child from the Democratic Republic of the Congo (DRC) in 1970 [4]. The symptoms of mpox include skin rashes, fever, and intense headache [5]. Mpox spreads by close contact with an animal infected with mpox virus, but also by skin-to-skin contact, respiratory droplets, or oral fluids during intimate sexual contact, and contact with fabrics, objects, or surfaces contaminated with mpox virus to achieve human-to-human spread [6]. Before May 2022, human mpox was an epidemic in African countries [5], and it was only occasionally imported to non-epidemic countries, such as Singapore [7], the United States [8], and the United Kingdom by travelers to places with circulating mpox (endemic areas) or with exposure to infected animals [9].

However, at the beginning of May 2022, after the United Kingdom informed the World Health Organization (WHO) about a confirmed case of mpox who returned from Nigeria to the United Kingdom, there were subsequently clusters of mpox virus infections in multiple non-epidemic countries [6, 10]. Between 1 January and 15 June 2022, a cumulative total of 2,103 laboratory-confirmed cases, one probable case, and one death had been reported to the WHO from 42 countries in five WHO regions [10]. The 2022 multiple-country mpox outbreak was affected by complex factors, including not being previously immunized against smallpox, stronger sexual transmission, and increased travel [10–12].

With the ongoing 2022 multiple-country mpox outbreak, the WHO is calling for more research to understand the differences in mpox epidemiology from that before 2022 [13]. One study reported 333 confirmed mpox cases from 2009 to 2014; the age of these cases ranged from 1 month to 67 years, and there were slightly more males (53.4%, 178/333) [14]. Yinka-Ogunleye et al. [15] reported that from 2017 to 2018, the proportion of male patients was 68.85% among 122 confirmed or probable cases aged from 2 days to 50 years. Previous studies showed that mpox cases before 2022 were mainly adolescents or young males. However, recent studies have reported that in 2022, mpox cases had a higher proportion of male patients and those of older age [16–18]. For example, Thornhill et al. reported that among 528 confirmed mpox cases, there were 527 male cases with a median age of 38 years [18]. In addition, several studies have reported that this outbreak differs from the previously reported mpox cases’ characteristics, such as in the high proportion of men who have sex with men (MSM) [17, 18].

Considering the above information, we need to explore the differences between cases before 2022 and those from 2022. Although one study has summarized case fatality rates (CFRs) of mpox before 2022, other epidemiological parameters, including incubation period, the secondary attack rate, animal contact history, and travel history, were not assessed [19]. In addition, it is worth noting that the 2022 outbreak is the first mpox outbreak simultaneously occurring in high-income and low-income countries, but there has been no research on the relationship between human mpox cases and social-economic levels. Therefore, we initiated a systematic literature review and meta-analysis to review the epidemiological and clinical characteristics of mpox cases, analyze the evolution since the first cases in the 1970s through the present day, and compare the key points of the mpox epidemic before 2022 and the 2022 multiple-country mpox outbreak. In addition, we evaluated the disparities in social-economic levels of human mpox cases by analyzing the relationship between the number of confirmed cases, sociodemographic index, human development index, healthcare access and quality index, and international travel arrivals.

## Methods

### Systematic Review and Meta-Analysis

In our study, we extracted data from the literature on mpox cases, including suspected cases, confirmed cases, probable cases, and possible cases. A specific case definition is shown in Supplementary Appendix, p. 1. If we were not able to classify cases as one of the abovementioned four specific types, we classified them as the “all cases” group [20]. We searched PubMed, Medline, Web of Science, and Embase for studies published until 14 June 2022. The literature search was based on the terms “Monkeypox” or “Monkeypox virus” or “monkey pox.” No language restrictions were applied. Two authors (SMZ and HMS) searched and screened the literature independently. The systematic literature review was reported in accordance with the Preferred Reporting Items for Systematic Reviews and Meta-Analyses (PRISMA) checklist 2020 (Supplementary Appendix, pp. 81–85). This review was registered in PROSPERO (CRD42022339404).

Studies irrelevant to the subject of the meta-analysis, studies with insufficient data, duplicate studies or those with overlapping participants, modeling studies that did not provide original data, and non-human studies were all excluded. Full-text articles were then critically evaluated independently by two researchers (SMZ and HMS) to determine whether at least one of the review objectives was met. For the eligible articles, data extraction was done independently by two authors (SMZ and HMS) with any disagreements arbitrated by a third author (MD). In addition, we collected unpublished data from five sources, namely the websites of the WHO (June 29, 2022; Multi-Country Mpox Outbreak: Situation Update), United States Centers for Disease Control and Prevention (CDC) (29 June 2022), African CDC (14 June 2022), Nigerian CDC (14 June 2022), and ProMed (14 June 2022). One researcher performed search of the gray literature (MD), and two researchers (SMZ and HMS) reviewed the findings and added the relevant information to the data extraction sheet.

The included articles were case reports, epidemiological studies, and surveillance data from the websites. For these types, no formal checklists for critical appraisal are available, so informal quality assessments were performed. Information on study quality was added based on a self-reported assessment with a total score of 10 (Supplementary Appendix, p. 2). [21]. This self-reported assessment was designed based on the 24 June 2022, *Surveillance, case investigation, and contact tracing for monkeypox—*Interim guidance from the WHO [21].

### Ecological Study

We extracted data on the total population (https://data.worldbank.org/indicator/SP.POP.TOTL) [22], healthcare access and quality index (HAQ) (https://ghdx.healthdata.org/record/ihme-data/gbd-2016-healthcare-access-and-quality-index-1990-2016) [23, 24], human development index (HDI) (https://hdr.undp.org/data-center/human-development-index#/indicies/HDI) [25], and sociodemographic index (SDI) (http://ghdx.healthdata.org/record/ihme-data/gbd-2019-socio-demographic-index-sdi-1950-2019) from World Bank and Global Burden of Disease Study (GBD) to explore the disparities in social-economic levels among different countries and territories during the 2022 multiple-country mpox outbreak. HAQ is calculated based on principal component analysis, providing an overall score of personal healthcare access and quality on a scale of 0–100 by the GBD team [24]. The HDI is a summary measure of average achievement in the following key dimensions of human development: a long and healthy life, being knowledgeable, and having a decent standard of living [25]. GBD researchers developed SDI as a composite indicator of total fertility rate among those aged <25 years, education level for those aged ≥15 years, and lag-distributed income *per capita* [26]. Additionally, we added the number of international travel arrivals (thousands) to analyze the effect of tourism on the 2022 multiple-country monkeypox outbreak. The data on international travel arrivals were obtained from the World Bank (https://data.worldbank.org/indicator/ST.INT.ARVL?name_desc=false) [27]. International travel arrivals are defined as international inbound tourists (overnight visitors) who travel to a country other than that in which they have their usual residence, but outside their usual environment, for a period not exceeding 12 months, and whose main purpose in visiting is other than an activity remunerated from within the country visited [27].

### Statistical Analysis

In the meta-analysis, the specific calculation method for average values and standard deviations of each study was based on common and optimal estimate methods [28–31]. Because specific clade data were not always reported in the literature, we used the geographical spread of the clades as described by the WHO to assign the clade variants as follows: Clade I (DRC, Gabon, Central African Republic, South Sudan, and Republic of the Congo) and Clade II (all other countries, except Cameroon because it had detected both clades) [19, 32]. Der Simonian and Laird random-effects models [33] were used to calculate the pooled effect and its 95% confidence interval (CI). We performed subgroup analyses, where the subgroups were based on the study design, location, national income level, cases reported time, type of cases, sample size, type of virus, and study quality scores. Publication bias was assessed by funnel plot and the Egger regression test [34]. We performed two sensitivity analyses to test the robustness of our results by excluding studies with sample size <5 and studies with quality scores ≤5.

In the ecological study, we presented the relationship between confirmed cases of mpox cases and HAQ (2015), HDI (2019), SDI (2019), and international travel arrivals (2019) using bubble and scatter charts. Moreover, their correlations were evaluated by Pearson correlation analyses.

All of the data analyses were completed using R software version 4.0.5 (R Foundation) and Stata 16.0 (StataCorp LLC, Texas, United States). Two-sided *p* < 0.05 indicated statistical significance.

## Results

**FIGURE 1:**
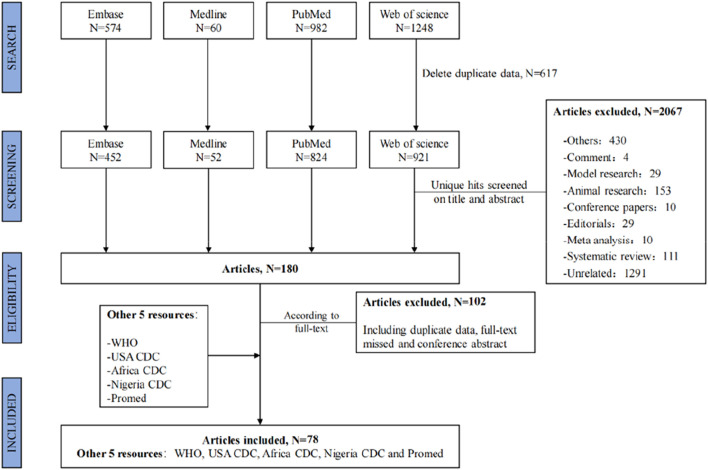
Flowchart of the selection process. Global epidemiological features of human monkeypox cases and their associations with social-economic level and international travel arrivals: a systematic review and ecological study (Global. 2022).

The search strategy yielded a total of 2,864 publications, 180 of which were selected for full-text screening. Of these, 78 articles were suitable for data extraction [3, 9, 14, 15, 35–108]. Additional gray literature extracted from the five website sources was also included for data extraction [1, 5, 10, 109, 110]. The flowchart of the selection process for the systematic review is shown in Figure 1.

### Pooled Estimates of Demographic, Clinical, and Epidemiological Characteristics for Two Periods (Before 2022 and in 2022) and Two Clades (Clades I and II)

Specific studies for estimates of demographic, clinical, and epidemiological characteristics are shown in Supplementary Tables S1–S10. The overall estimates and their other subgroup analysis except for periods (before 2022 and in 2022) and clades (Clades I and II) are shown in Supplementary Tables S11–S19 and Supplementary Figures S1–S12.

We observed that the average age of 167 mpox cases reported in 2022 (35.52 years, 95% CI [28.09, 42.94]) was higher than that of 3,346 mpox cases reported before 2022 (18.38 years, 95% CI [14.74, 22.02]) (Table 1; Supplementary Figure S2). The comorbidity rate was 14.9% (95% CI [8.5%, 21.3%]) before 2022, while it increased to 15.7% (95% CI [8.9%, 22.4%]) in 2022 (Table 1; Supplementary Figure S6). CFR was higher in 2022 (4.7%, 95% CI [3.0%, 6.3%]) than before 2022 (3.4%, 95% CI [2.9%, 4.0%]) (Table 1; Supplementary Figure S8). We observed that the proportion of travel history was 37.0% (95% CI [27.1%, 46.9%]) before 2022, while it was reduced to 29.6% (95% CI [3.7%, 55.5%]) in 2022 without reaching statistical significance (Table 1; Supplementary Figure S12). All nine studies reporting patients who identified as MSM were in the high-income region in 2022. We estimated that the proportion of MSM was 79.8% (95% CI [65.5%, 94.2%]) among 510 cases (Table 1).

**TABLE 1:** Estimates of demographic, clinical and epidemiological characteristics among all mpox cases by two periods (before 2022 and 2022). Global epidemiological features of human monkeypox cases and their associations with social-economic level and international travel arrivals: a systematic review and ecological study (Global. 2022).

	No. studies	Sample size (N or n/N)	Effect	Lower limit	Higher limit
Average of age (years)					
Before 2022	31	3,346	18.38	14.74	22.02
In 2022	5	167	35.52	28.09	42.94
Comorbidity rate (%)					
Before 2022	14	19/117	14.9%	8.5%	21.3%
In 2022	4	336/1,801	15.7%	8.9%	22.4%
Case fatality rate (%)					
Before 2022	37	1,368/60,365	3.4%	2.9%	4.0%
In 2022	9	163/3,298	4.7%	3.0%	6.3%
Proportion of travel history (%)					
Before 2022	6	158/443	37.0%	27.1%	46.9%
In 2022	7	106/307	29.6%	3.7%	55.5%
Proportion of MSM (%) in 2022^a^	9	392/510	79.8%	65.5%	94.2%

^a^ Notes: Proportion of MSM (%) in 2022 was only reported in studies in 2022. MSM, men who make sex with men.

For different clades, the comorbidity rate (%) of 1,468 mpox cases infected with Clade I (20.4%, 95% CI [10.6%, 30.3%]) was higher than that of 450 mpox cases infected with Clade II (9.8%, 95% CI [4.8%, 14.8%]). In addition, the proportion of animal contact history (%) of 351 mpox cases infected with Clade II (45.1%, 95% CI [23.7%, 66.4%]) was higher than that of 4,994 mpox cases infected with Clade I (34.7%, 95% CI [16.3%, 53.1%]) (Table 2).

**TABLE 2:** Estimates of demographic, clinical and epidemiological characteristics among all mpox cases by two clades (clade I and clade II)^a^. Global epidemiological features of human monkeypox cases and their associations with social-economic level and international travel arrivals: a systematic review and ecological study (Global. 2022).

	No. studies	Sample size (N or n/N)	Effect	Lower limit	Higher limit
Average of age (years)					
Clade I	17	2,999	14.54	13.24	15.84
Clade II	18	507	30.40	26.27	34.54
Proportion of male patients (%)					
Clade I	22	2,370/4,149	55.4%	51.6%	59.2%
Clade II	20	498/808	60.4%	51.6%	69.3%
Duration of symptoms (days)					
Clade I	1	282	16.38	6.05	26.72
Clade II	3	71	10.37	5.73	15.00
Comorbidity rate (%)					
Clade I	6	310/1,468	20.4%	10.6%	30.3%
Clade II	12	45/450	9.8%	4.8%	14.8%
Case fatality rate (%)					
Clade I	30	1,393/60900	3.5%	2.8%	4.1%
Clade II	12	49/1,131	3.6%	2.5%	4.7%
Secondary attack rate (%)					
Clade I	5	523/10716	4.5%	2.4%	6.7%
Clade II	3	15/493	3.9%	−1.4%	9.2%
Incubation period (days)					
Clade I	1	16	9.17	4.18	14.15
Clade II	4	80	10.26	4.51	16.00
Proportion of animal contact history (%)					
Clade I	17	2,325/4,994	34.7%	16.3%	53.1%
Clade II	8	131/351	45.1%	23.7%	66.4%
Proportion of travel history (%)					
Clade I	2	148/425	32.0%	18.6%	45.5%
Clade II	11	116/325	36.5%	14.6%	58.3%
Proportion of MSM (%) in 2022^b^	9	392/510	79.8%	65.5%	94.2%

^a^ Notes: Congo Basin or Central African clade (Clade I) and West African clade (Clade II).

^b^ Proportion of MSM (%) in 2022 was only reported in studies in 2022 which all were clade II. MSM, men who make sex with men.

Sensitivity analysis showed that all results were stable (Supplementary Appendix, pp. 65–66). Funnel plots for all of the estimations are shown in Supplementary Figures S13–S15. Egger regression tests for the average age (z = 0.62; *p* = 0.536), proportion of male patients (z = −0.97; *p* = 0.332), average duration of symptoms (z = 1.82; *p  *=  0.069), secondary attack rate (SAR) (z = 1.21; *p* = 0.227), average incubation period (z = 0.67; *p* = 0.503), proportion of animal contact history (z = −0.18; *p* = 0.860), proportion of travel history (z = 0.62; *p* = 0.532), and proportion of MSM (z = −1.61; *p* = 0.107) indicated that there was no publication bias, except for the estimation of the comorbidity rate (z = 3.55; *p* < 0.001) and the CFR (z = 6.33; *p* < 0.0001).

There was one study reporting four pregnant women and their pregnancy outcomes [52]. All four cases had normal hematological and clinical chemistry findings, except for a decreased albumin level [52]. Of the four pregnant women, only one gave birth to a healthy infant, while three experienced fetal demise [52]. One of the three was stillborn, with the macerated stillborn showing diffuse cutaneous maculopapular skin lesions involving the head, trunk, and extremities, including palms of hands and soles of feet [52]. Fetal tissue, placental levels, and cord vein blood all had similarly high levels of the virus [52]. A very high viral load likely resulting in placental proinflammatory cytokine release may have been the mechanism of injury [52]. Currently, no vaccine against mpox is approved for use in pregnancy.

### Association Between SDI, HAQ, HDI, International Arrivals and Human Mpox Cases

Supplementary Figure S16 presents the progression of mpox epidemic countries and territories (sources shown in Supplementary Table S20) [111, 112]. Up to 27 June 2022, the main epidemic region was the European region, and it expanded to other continents outside Africa, including the Americas, the Western Pacific region, and the Eastern Mediterranean region.

**FIGURE 2:**
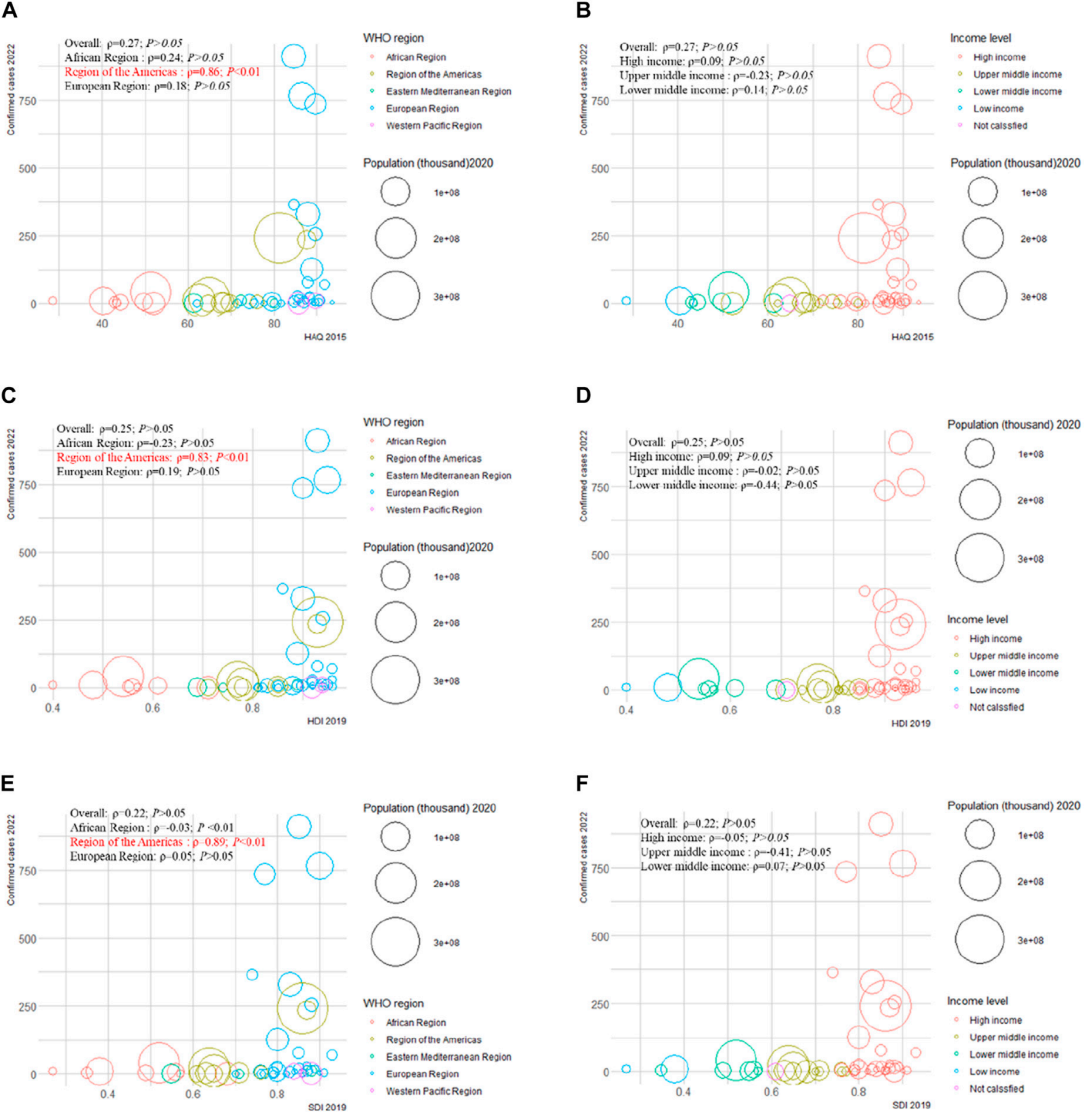
Mpox confirmed cases at the country and territorial levels. Global epidemiological features of human monkeypox cases and their associations with social-economic level and international travel arrivals: a systematic review and ecological study (Global. 2022). Confirmed mpox cases were reported by the WHO and CDC on 27 June 2022. The size of the circles increased with the population. The *ρ* indices and *p* values were derived from Pearson correlation analysis. **(A)** correlation between confirmed mpox cases and HAQ by WHO region; **(B)** correlation between confirmed mpox cases and HAQ by income level; **(C)** correlation between confirmed mpox cases and HDI by WHO region; **(D)** correlation between confirmed mpox cases and HDI by income level; **(E)** correlation between confirmed mpox cases and SDI by WHO region; **(F)** correlation between confirmed mpox cases and SDI at income level. CDC, center for disease control and prevention; HAQ, healthcare access and quality index; HDI, human development index; SDI, sociodemographic index; WHO, World Health Organization.

**FIGURE 3:**
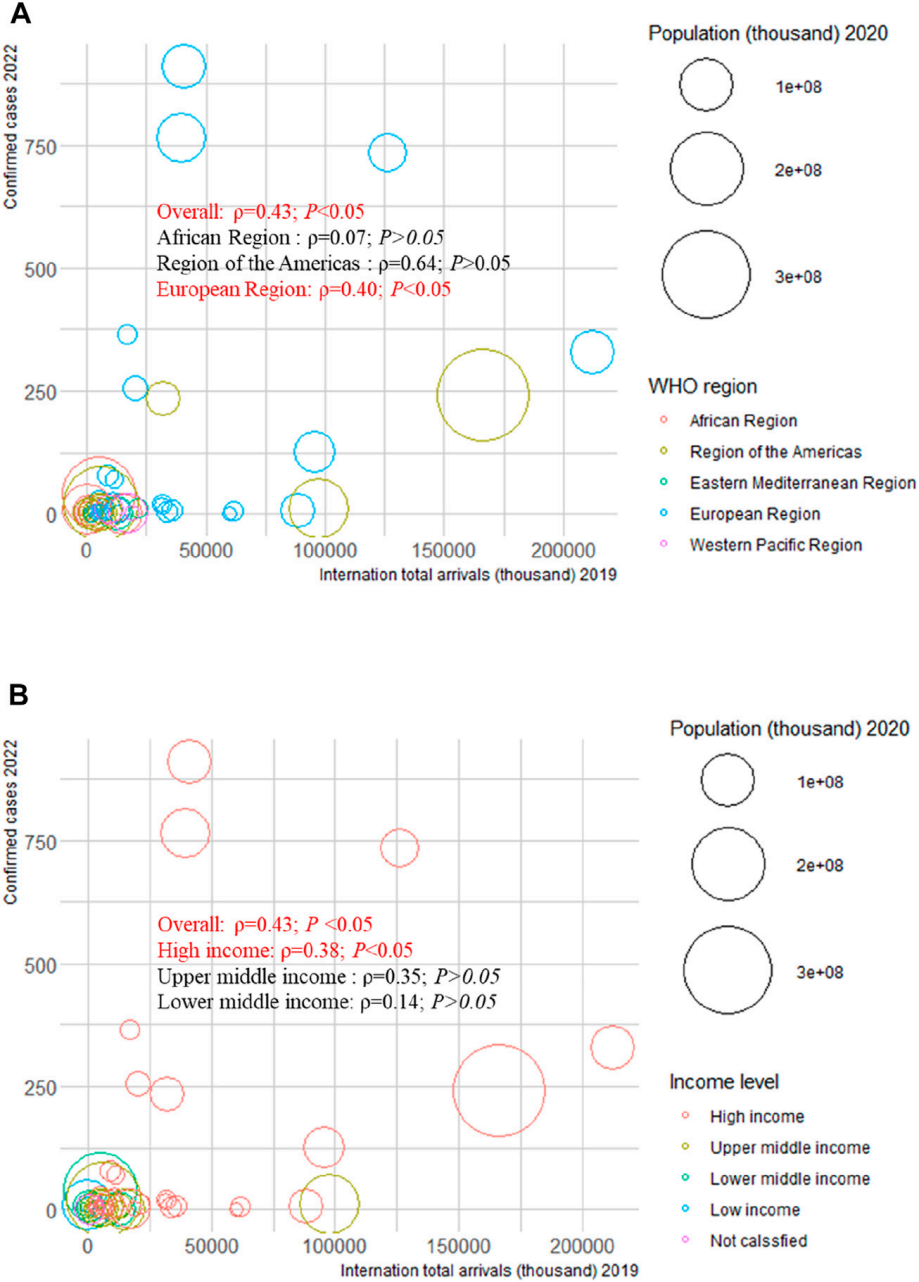
Mpox confirmed cases at the country and territorial levels. Global epidemiological features of human monkeypox cases and their associations with social-economic level and international travel arrivals: a systematic review and ecological study (Global. 2022). Confirmed mpox cases were reported by the WHO and CDC on 27 June 2022. The size of the circles increased with the population. The *ρ* indices and *p* values were derived from Pearson correlation analysis. **(A)** correlation between confirmed mpox cases and international travel arrivals by WHO region; **(B)** correlation between confirmed mpox cases and international travel arrivals by income level. CDC, center for disease control and prevention; WHO, World Health Organization.

We examined the correlation between confirmed cases in 2022 and HAQ in 2015, HDI in 2019, SDI in 2019, and international total arrivals in 2019 among the 55 countries (all original values are shown in Supplementary Table S21). Surprisingly, a significant positive correlation was detected between confirmed cases and HAQ in 2015 (*ρ* = 0.86; *p* < 0.01), HDI in 2019 (*ρ* = 0.83; *p* < 0 .01), and SDI in 2019 (*ρ* = 0.89; *p* < 0.01) in the American region (Figure 2). As of 27 June 2022, the top three countries with the highest number of confirmed cases in the 2022 multiple-country mpox outbreak were the United Kingdom (910), Germany (765), and Spain (736) (Supplementary Figure S17A); the top three countries with the highest number of international total arrivals in 2020 were France (211998), the United States (166009), and Spain (126170) (Supplementary Figure S17B). There was a significant correlation between confirmed cases and international total arrivals in 2019 (*ρ* = 0.40; *p* < 0.05) among the 55 countries, and it remained significant in the European region (*ρ* = 0.40; *p* < 0.05) and high-income region (*ρ* = 0.38; *p* < 0.05) (Figure 3).

## Discussion

To the best of our knowledge, this is the first study reviewing the latest global epidemiological and clinical characteristics of mpox cases from 1970 to 2022 and then reporting estimates by two periods and clades. We estimated demographic characteristics (average age and proportion of male patients), clinical characteristics (duration of symptoms and comorbidity rate), and epidemiological characteristics (CFR, SAR, average incubation period, proportion of animal contact history, proportion of travel history, and proportion of MSM). We also reviewed maternal and fetal outcomes among pregnant women.

The average age of mpox cases was 21.05 years. Mpox cases reported in 2022 were older than those before 2022. Our study estimated that the proportion of male patients was 57.9%, and it was higher in the European region. Up to now, the 2022 Mpox Outbreak Global Map shows that the top three countries are the United Kingdom, Germany, and Spain—all in the European region—the main epidemic region [1]. It was reported that the 2022 multiple-country outbreak of mpox virus belonged to Clade II [20]. Girometti et al. reported 54 individuals with confirmed mpox, who identified themselves as MSM, with a median age of 41 years in London, between 14 May and 25 May 2022 [16]. Perez Duque et al. reported that 27 male confirmed cases of mpox had a median age of 33 years in Portugal [36]. However, the median age of 1,057 confirmed cases with 53.7% males in DRC from 2011 to 2015 was 14.0 years [37]. Our findings indicate that mpox virus infection mainly occurs in young males, but the 2022 multiple-country outbreak of mpox virus shows a higher proportion of older patients than before. This demographic change may hint that the high-risk population in 2022 is older.

The average duration of symptoms was 11.41 days, and it was lower in the high-income region and the Americas than in the low-income region and African region. The high-income region and the Americas generally have a higher quality of medical service [113]. In addition, sites of skin lesions and proportion of fever have changed in the 2022 multiple-country mpox outbreak, compared with that before 2022. In London, between 14 May and 25 May 2022, 54 confirmed cases of mpox, who identified themselves as MSM, presented a lower proportion of fever (see Box, Supplementary Appendix, pp. 79–80) [14–16, 59]. The most likely site of lesions changed from face to genitals in 2022 (see Box, Supplementary Appendix, pp. 79–80) [14–16, 37, 59]. As presentation from box, it may be possible that MSM are infected with mpox virus through sexual contact as rashes are more likely to occur on the genitals; in contrast, the lesions are more likely to occur on the face or hands in cases with a higher percentage of exposure to animals [15, 16, 37]. In our study, the estimated CFR was 3.8%, and patients reported in 2022 had a higher CFR. According to the WHO, in recent times, the CFR has been approximately 3%–6% in the general population and has been higher among younger people [19, 32]. Our study reported that the comorbidity rate of mpox cases was 16%, and it was higher in 2022. We also estimated that the proportion of MSM was 80%. The latest literature from the United Kingdom showed that nearly 100% of patients in London between 14 May and 25 May 2022, were MSM [16]. Thus far, many patients in the United Kingdom and other non-endemic countries are men who are gay, bisexual, and have sex with men [113]. Our findings suggest that attention should be paid to the severity of diseases among patients with comorbidities so as to avoid fatal outcomes and the spreading of mpox among MSM.

Mpox virus is transmitted between animals and humans, and from human to human [113]. However, the mpox virus outbreak in Portugal from 29 April to 23 May 2022 showed that most cases were neither part of identified transmission chains nor linked to travel or had contact with symptomatic people or with animals [36]. We estimated that the proportion of animal contact history and travel history was 38% and 34%, respectively. Animal-to-human transmission mainly occurred in the African region, and it caused clusters of mpox cases in the United States in 2003 [114]. According to Haider et al., the diversity and extent of the animal reservoir for mpox remain unknown, and the synanthropic rodent population has increased in recent years in Africa, leading to more human–rodent interactions and thus increased transmission [115]. Additionally, for non-epidemic countries, travel may be an important factor to promote human-to-human transmission [8, 114, 116, 117]. We found a significant correlation between confirmed cases and international total arrivals in 2019. The sudden and unexpected simultaneous appearance of mpox in several non-endemic countries suggests that there might have been undetected transmission for a long time, amplified by recent large social events and increased travel [113]. Our study suggests that it may be necessary to pay attention to the mpox epidemic of animal reservoir and travelers, especially patients during the incubation period. In this sense, strengthening epidemiological surveillance systems and disseminating adequate information through reliable channels (official social media and web pages) with clear and assertive messages could contribute to gaining greater confidence from the broad public and assisting in early case detection, thereby halting transmission chains and preventing further outbreaks [118].

The strengths of this review are that it included a broad search strategy on mpox worldwide, without time or language limits, which reduced selection bias. In addition, there was a thorough review of the gray literature for comprehensive data extraction. However, there were some limitations. First, mpox may occur in some countries where it could be unreported or undetected; therefore, due to data availability, our results may underestimate the real-world data. Second, since specific data on the clades were infrequently reported, we assigned clades based on the geographical spread described by the WHO. However, these may not be fully consistent with the reported cases, so our results may have information bias. Third, although there were articles presenting data on the transmission of mpox, many studies did not attribute cases to animal-to-human transmission or human-to-human transmission. Therefore, we could not analyze the changes over time, CFR, and SAR among different transmission routes. Finally, there was a lack of studies that reported the proportion of MSM before 2022, so we could not compare it in 2022 with that before 2022. In addition, the majority of cases before 2022 in Central and Western Africa were never published; although we included data from African CDC and Nigerian CDC, data were skewed toward the 2022 cases due to reporting bias.

In conclusion, our study provided the estimation of the average age, proportion of male patients, average duration of symptoms, comorbidity rate, CFR, SAR, average incubation period, proportion of animal contact history, proportion of travel history, and proportion of MSM. We observed that the average age and comorbidity rate in 2022 were higher than those before 2022. Confirmed cases of the 2022 multiple-country mpox outbreak correlated with international total arrivals in 2020 among 55 countries. The multiple-country outbreak of mpox in 2022 highlights the importance of urgent response and global cooperation in coping with the transmission and impact of the disease. Except for providing information on the pooled estimates, our study also emphasized the demographic changes and the comorbidity rate in 2022, compared with before 2022. Additionally, we focused on the high 2022 proportion of MSM and the positive relationship between travelers and confirmed cases in 2022. To understand and explore the changing epidemiology of the mpox epidemic, increased surveillance and timely detection are crucial tools, especially in high-risk populations, including older people, MSM, and travelers.
