# Supplementary material for: Global Epidemiological Features of Human Monkeypox Cases and Their Associations With Social-Economic Level and International Travel Arrivals: A Systematic Review and Ecological Study
Source: Int J Public Health. 2023 Jan 20;68:1605426. doi: 10.3389/ijph.2023.1605426 (PMC9894882; doi:10.3389/ijph.2023.1605426)
Supplement: Supplementary file 1 [file DataSheet1.docx]

**Supplementary appendix**

This appendix formed part of the original submission.

Global epidemiological features of human monkeypox cases and their associations with social-economic level and international travel arrivals: A systematic review and ecological study

**Appendices**

**2022-12-26**

**Monkeypox case definitions were as followings:**

1. Suspected cases: Sudden onset of high fever, followed by a vesicular-pustule eruption presenting predominantly on the face, palms of the hands, and soles of the feet; or the presence of at least 5 smallpox type scabs [1].
2. Confirmed cases: Suspected case with laboratory confirmation (Positive IgM Antibody, PCR, or virus isolation) [1].
3. Probable cases: Suspected case with no possibility of laboratory confirmation, but with epidemiological link to a confirmed case [1].
4. Possible cases: Vesicular, pustular or crusted rash, not diagnosed as chickenpox by the family or the health-care provider; history of fever and vesicular or crusty rash; individual met one of the epidemiologic criteria or demonstrated elevated levels of orthopoxvirus-specific IgM and had unexplained rash and fever and ≥2 other signs or symptoms from the clinical criteria [1].

Our self-reported assessment for study quality was designed based on 24 June 2022 *Surveillance, case investigation and contact tracing for monkeypox-* Interim guidance from world health organization [2]. We considered the study design, case definition diagnostic test, demographic information, diagnostic test of suspected cases and clinical and risk factor information.

Specific Questions were following (total scores were 10):

**Study design** Q1. The study design was?

• case reports, cross-sectional studies, cohort studies, government reports – **2 points**

• others (eg, mail)– **1 point**

• No/unclear – 0 points

**Case definition** Q2. Whether common/standard definitions were used?

• Yes – **1 point**

• No/unclear – 0 points

**Diagnostic test** Q3. Whether laboratory confirmation (Positive IgM Antibody, PCR, or virus isolation) was used?

• Yes – **2 points**

• Not described but data from government reports – **1 point**

• No/unclear – 0 points

**Demographic information** Q4. Whether complete demographic information (Age, sex) was reported?

• at least two – **2 points**

• one – **1 point**

• No/unclear – 0 points

**Diagnostic test of suspected cases** Q5. Whether suspected cases with laboratory testing performed was reported?

• yes – **1 point**

• No/unclear – 0 points

**Clinical and risk factor information** Q6. Whether complete clinical and risk factor information was reported?

• both two– **2 points**

• one – **1 point**

• No/unclear – 0 points

**Table S1: Information and indicators of original literature related to average age (Global. 2022)**

| Title | Study design | Author | Year | Country | WHO region | Income level | Reported time | Cases | Sample size1 | Sample size | Average of age | Standard deviation of age | Virus | Quality scores |
| --- | --- | --- | --- | --- | --- | --- | --- | --- | --- | --- | --- | --- | --- | --- |
| Community transmission of monkeypox in the United Kingdom, April to May 2022[3] | case report | Roberto Vivancos | 2022 | The United Kingdom | European Region | High income | 2022 | confirmed cases | 82.00 | 5~99 | 37.6476 | 8.3009 | West African clade | >5 |
| Ongoing monkeypox virus outbreak, Portugal, 29 April to 23 May 2022[4] | case report | Mariana Perez Duque | 2022 | Portugal | European Region | High income | 2022 | confirmed cases | 27.00 | 5~99 | 35.5006 | 22.6979 | West African clade | >5 |
| Clinical and Epidemiological Findings from Enhanced Monkeypox Surveillance in Tshuapa Province, Democratic Republic of the Congo During 2011–2015[5] | cross-sectional study | Erin R. Whitehouse | 2021 | Democratic Republic of the Congo | African Region | Low income | before2022 | confirmed cases | 1057.00 | >500 | 14.9497 | 13.0863 | Central African clade | >5 |
| A Tale of Two Viruses: Coinfections of Monkeypox and Varicella Zoster Virus in the Democratic Republic of Congo[6] | cross-sectional study | Christine M. Hughes | 2021 | Democratic Republic of the Congo | African Region | Low income | before2022 | confirmed cases | 400.00 | 100~500 | 14.639 | 11.2677 | Central African clade | >5 |
| Clinical Course and Outcome of Human Monkeypox in Nigeria[7] | cross-sectional study | Dimie Ogoina | 2020 | Nigeria | African Region | Lower middle income | before2022 | confirmed cases | 40.00 | 5~99 | 31.003 | 12.5033 | West African clade | >5 |
| Descriptive epidemiology of Monkeypox outbreak in Bayelsa State South-South Nigeria, November 2017[8] | cross-sectional study | M. Ibegu | 2020 | Nigeria | African Region | Lower middle income | before2022 | all cases | 30.00 | 5~99 | 24.583 | 32.6936 | West African clade | >5 |
| Monkeypox Rash Severity and Animal Exposures in the Democratic Republic of the Congo[9] | cross-sectional study | Reena H. Doshi | 2019 | Democratic Republic of the Congo | African Region | Low income | before2022 | confirmed cases | 223.00 | 100~500 | 11.6491 | 6.7155 | Central African clade | >5 |
| Human Monkeypox in Sierra Leone after 44-Year Absence of Reported Cases[10] | case report | Mary G. Reynolds | 2019 | Sierra Leone | African Region | Low income | before2022 | confirmed cases | 2.00 | <5 | 17.958333 | 10.778096 | West African clade | >5 |
| Monkeypox virus phylogenetic similarities between a human case detected in Cameroon in 2018 and the 2017-2018 outbreak in Nigeria[11] | case report | Serge AlainSadeuh-Mba | 2019 | Cameroon | African Region | Lower middle income | before2022 | suspected cases | 7.00 | 5~99 | 25.9042 | 21.2323 | Cameroon | >5 |
| The 2017 human monkeypox outbreak in Nigeria—Report of outbreak experience and response in the Niger Delta University Teaching Hospital, Bayelsa State, Nigeria[12] | cross-sectional study | Dimie Ogoina | 2019 | Nigeria | African Region | Lower middle income | before2022 | all cases | 21.00 | 5~99 | 27.5113 | 9.8428 | West African clade | >5 |
| Epidemiologic and Ecologic Investigations of Monkeypox, Likouala Department, Republic of the Congo, 2017[13] | cross-sectional study | Reena H. Doshi | 2019 | Republic of the Congo | African Region | Lower middle income | before2022 | all cases | 22.00 | 5~99 | 14.0427 | 10.2114 | Central African clade | >5 |
| Intrafamily Transmission of Monkeypox Virus, Central African Republic, 2018[14] | cross-sectional study | Camille Besombes | 2019 | Central African Republic | African Region | Low income | before2022 | confirmed cases | 6.00 | 5~99 | 15.3829 | 12.7125 | Central African clade | >5 |
| Exportation of Monkeypox Virus From the African Continent[15] | cross-sectional study | Matthew R Mauldin | 2022 | Nigeria | African Region | Lower middle income | before2022 | confirmed cases | 6.00 | 5~99 | 35.666667 | 3.8815804 | West African clade | ≤5 |
| Letter to the editor: multiple introductions of MPX in Italy from different geographic areas[16] | others | Federica Ferraro | 2022 | Italy | European Region | High income | 2022 | confirmed cases | 29.00 | 5~99 | 36.2425 | 8.3897 | West African clade | >5 |
| Trend and enhanced surveillance of Monkeypox during COVID-19 pandemic in Nigeria[17] | cross-sectional study | Lateefat Kikelomo Amao | 2022 | Nigeria | African Region | Lower middle income | before2022 | confirmed cases | 3.00 | <5 | 29.666667 | 10.263203 | West African clade | >5 |
| Investigation of an outbreak of monkeypox in an area occupied by armed groups, Central African Republic[18] | cross-sectional study | [E Kalthan](https://pubmed.ncbi.nlm.nih.gov/?sort=date&term=Kalthan+E&cauthor_id=29573840) | 2018 | Central African Republic | African Region | Low income | before2022 | confirmed cases | 26.00 | 5~99 | 25.4181 | 14.3885 | Central African clade | >5 |
| Enhancing case definitions for surveillance of human monkeypox in the Democratic Republic of Congo[19] | cross-sectional study | Osadebe, Lynda | 2017 | Democratic Republic of the Congo | African Region | Low income | before2022 | confirmed cases | 333.00 | 100~500 | 14.7825 | 11.5444 | Central African clade | >5 |
| A Nosocomial Outbreak of Human Monkeypox in the Central African Republic[20] | case report | Nakoune, Emmanuel | 2017 | Central African Republic | African Region | Low income | before2022 | all cases | 10.00 | 5~99 | 24.8502 | 12.8505 | Central African clade | >5 |
| Maternal and Fetal Outcomes Among Pregnant Women With Human Monkeypox Infection in the Democratic Republic of Congo[21] | case report | Mbala, Placide K. | 2017 | Democratic Republic of the Congo | African Region | Low income | before2022 | confirmed cases | 4.00 | <5 | 24 | 3.91578 | Central African clade | >5 |
| Extended human-to-human transmission during a monkeypox outbreak in the Democratic Republic of the Congo[22] | cross-sectional study | Nolen, L. D. | 2016 | Democratic Republic of the Congo | African Region | Low income | before2022 | confirmed cases | 20.00 | 5~99 | 20.0438 | 18.0205 | Central African clade | ≤5 |
| Cytokine modulation correlates with severity of monkeypox disease in humans[23] | others | Johnston, S. C. | 2015 | Democratic Republic of the Congo | African Region | Low income | before2022 | confirmed cases | 19.00 | 5~99 | 11.773684 | 8.4027669 | Central African clade | ≤5 |
| Detection of human monkeypox in the Republic of the Congo following intensive community education[24] | case report | Reynolds, M. G. | 2013 | Republic of the Congo | African Region | Lower middle income | before2022 | all cases | 7.00 | 5~99 | 9.1428571 | 4.3369948 | Central African clade | >5 |
| Maculopapular lesions in the Central African Republic[25] | case report | Berthet, N. | 2010 | Central African Republic | African Region | Low income | before2022 | confirmed cases | 2.00 | <5 | 14.5 | 0.7071068 | Central African clade | >5 |
| Major increase in human monkeypox incidence 30 years after smallpox vaccination campaigns cease in the Democratic Republic of Congo[26] | cross-sectional study | Rimoin, Anne W. | 2010 | Democratic Republic of the Congo | African Region | Low income | before2022 | confirmed cases | 760.00 | >500 | 10.6725 | 11.1162 | Central African clade | >5 |
| Occupational risks during a monkeypox outbreak, Wisconsin, 2003[27] | cross-sectional study | Croft, D. R. | 2007 | United States | Region of the Americas | High income | before2022 | all cases | 27.00 | 5~99 | 27.3689 | 11.2693 | West African clade | >5 |
| Clinical characteristics of human monkeypox, and risk factors for severe disease[28] | cross-sectional study | Huhn, G. D. | 1980 | United States | Region of the Americas | High income | before2022 | confirmed cases | 34.00 | 5~99 | 26.1106 | 9.8023 | West African clade | >5 |
| Human monkeypox infection: a family cluster in the midwestern United States[29] | case report | Sejvar, James J. | 2004 | United States | Region of the Americas | High income | before2022 | confirmed cases | 3.00 | <5 | 23 | 14.798649 | West African clade | >5 |
| Multistate outbreak of monkeypox--Illinois, Indiana, and Wisconsin, 2003[30] | case report | Centers for Disease Control and Prevention (CDC) | 2003 | United States | Region of the Americas | High income | before2022 | all cases | 53.00 | 5~99 | 26.423 | 10.8138 | West African clade | >5 |
| Update: multistate outbreak of monkeypox--Illinois, Indiana, Kansas, Missouri, Ohio, and Wisconsin, 2003[31] | case report | Centers for Disease Control and Prevention (CDC) | 2003 | United States | Region of the Americas | High income | before2022 | all cases | 71.00 | 5~99 | 28.0703 | 28.0703 | West African clade | >5 |
| Outbreaks of disease suspected of being due to human monkeypox virus infection in the Democratic Republic of Congo in 2001[32] | cross-sectional study | Meyer, H. | 2002 | Democratic Republic of the Congo | African Region | Low income | before2022 | suspected cases | 14.00 | 5~99 | 10.035714 | 7.6522101 | Central African clade | >5 |
| Outbreak of human monkeypox, Democratic Republic of Congo, 1996-1997[33] | cross-sectional study | Hutin, Y. J. F. | 2001 | Democratic Republic of the Congo | African Region | Low income | before2022 | all cases | 88.00 | 5~99 | 12.5714 | 12.6202 | Central African clade | >5 |
| Human monkeypox, 1970-79[34] | cross-sectional study | Breman, J. G. | 1980 | Zaire (38), Liberia (4), Nigeria (3), Ivory Coast (1), and Sierra Leone(1) | other | other | before2022 | all cases | 47.00 | 5~99 | 6.4654 | 7.8716 | West African clade | >5 |
| Simian smallpox (or monkey smallpox): Study of 8 cases observed at Impfondo hospital in Republic of Congo[35] | case report | Boumandouki, P. | 2007 | Republic of the Congo | African Region | Lower middle income | before2022 | confirmed cases | 8.00 | 5~99 | 9.05 | 5.86 | Central African clade | >5 |
| Transmission of monkeypox among persons exposed to infected prairie dogs in Indiana in 2003[36] | cross-sectional study | Kile, J. C. | 2005 | United States | Region of the Americas | High income | before2022 | confirmed cases | 3.00 | <5 | 23 | 14.798649 | West African clade | >5 |
| Monkeypox update (09)[37] | others | promed mail | 2022 | Portugal | European Region | High income | 2022 | all cases | 27.00 | 5~99 | 33.8836 | 7.2624 | West African clade | >5 |
| Monkeypox update (01)[37] | others | promed mail | 2022 | Australia | Western Pacific Region | High income | 2022 | confirmed cases | 2.00 | <5 | 35 | 7.0710678 | West African clade | >5 |

**Notes：**Reported time refers to the year of reported cases (classified as 2022 group before 2022); Virus strain was classified as West African clade and Central African clade based on WHO; Quality Score was classified as >5 and ≤5; Study design includes case report, cross-sectional study, cohort study, case-control study, others (CDC website, promed mail etc.); Sample size includes >5, 5~99, 100~500 and >500 four groups; Cases type: we extracted data reported the monkeypox cases including suspected cases, confirmed cases, probable cases and possible cases based on reported literature, if literature reported monkeypox cases including multiple cases types, we classified it as “all cases” group.

**Table S2: Information and indicators of original literature related to proportion of male patients (Global. 2022)**

| Title | Study design | Author | Year | Country | WHO region | Income level | Reported time | Cases | Sample size2 | Sample size | Male | Virus | Quality scores |
| --- | --- | --- | --- | --- | --- | --- | --- | --- | --- | --- | --- | --- | --- |
| Clinical features and management of human monkeypox: a retrospective observational study in the UK[38] | cross-sectional study | Hugh Adler | 2022 | The United Kingdom | European Region | High income | before2022 | confirmed cases | 7.00 | 5~99 | 4.00 | West African clade | >5 |
| Clinical and Epidemiological Findings from Enhanced Monkeypox Surveillance in Tshuapa Province, Democratic Republic of the Congo During 2011–2015[5] | cross-sectional study | Erin R. Whitehouse | 2021 | Democratic Republic of the Congo | African Region | Low income | before2022 | confirmed cases | 1054.00 | 500 | 568.00 | Central African clade | >5 |
| Clinical Course and Outcome of Human Monkeypox in Nigeria[7] | cross-sectional study | Dimie Ogoina | 2020 | Nigeria | African Region | Lower middle income | before2022 | confirmed cases | 40.00 | 5~99 | 31.00 | West African clade | >5 |
| Descriptive epidemiology of Monkeypox outbreak in Bayelsa State South-South Nigeria, November 2017[8] | cross-sectional study | M. Ibegu | 2020 | Nigeria | African Region | Lower middle income | before2022 | all | 30.00 | 5~99 | 20.00 | West African clade | >5 |
| Monkeypox Rash Severity and Animal Exposures in the Democratic Republic of the Congo[9] | cross-sectional study | Reena H. Doshi | 2019 | Democratic Republic of the Congo | African Region | Low income | before2022 | confirmed cases | 223.00 | 100~500 | 155.00 | Central African clade | >5 |
| Descriptive epidemiology of monkeypox in Nigeria, September 2017–June 2019[39] | cross-sectional study | S. Akar | 2020 | Nigeria | African Region | Lower middle income | before2022 | confirmed cases | 165.00 | 100~500 | 115.00 | West African clade | ≤5 |
| Outbreak of human monkeypox in Nigeria in 2017–18: a clinical and epidemiological report[40] | cross-sectional study | Gemma Hobson | 2019 | Nigeria | African Region | Lower middle income | before2022 | all | 122.00 | 100~500 | 84.00 | West African clade | >5 |
| Monkeypox virus phylogenetic similarities between a human case detected in Cameroon in 2018 and the 2017-2018 outbreak in Nigeria[11] | case report | Serge AlainSadeuh-Mba | 2019 | Cameroon | African Region | Lower middle income | before2022 | suspected cases | 7.00 | 5~99 | 4.00 | Cameroon | >5 |
| The 2017 human monkeypox outbreak in Nigeria—Report of outbreak experience and response in the Niger Delta University Teaching Hospital, Bayelsa State, Nigeria[12] | cross-sectional study | Dimie Ogoina | 2019 | Nigeria | African Region | Lower middle income | before2022 | all | 21.00 | 5~99 | 17.00 | West African clade | >5 |
| Epidemiologic and Ecologic Investigations of Monkeypox, Likouala Department, Republic of the Congo, 2017[13] | cross-sectional study | Reena H. Doshi | 2019 | Republic of the Congo | African Region | Lower middle income | before2022 | all | 22.00 | 5~99 | 8.00 | Central African clade | >5 |
| Exportation of Monkeypox Virus From the African Continent[15] | cross-sectional study | Matthew R Mauldin | 2022 | Nigeria | African Region | Lower middle income | before2022 | confirmed cases | 6.00 | 5~99 | 5.00 | West African clade | ≤5 |
| Letter to the editor: multiple introductions of MPX in Italy from different geographic areas[16] | others | Federica Ferraro | 2022 | Italy | European Region | High income | 2022 | confirmed cases | 29.00 | 5~99 | 28.00 | West African clade | >5 |
| Trend and enhanced surveillance of Monkeypox during COVID-19 pandemic in Nigeria[17] | cross-sectional study | Lateefat Kikelomo Amao | 2022 | Nigeria | African Region | Lower middle income | before2022 | suspected cases | 19.00 | 5~99 | 9.00 | West African clade | >5 |
| Do Monkeypox Exposures Vary by Ethnicity? Comparison of Aka and Bantu Suspected Monkeypox Cases[41] | cross-sectional study | Sarah Anne J. Guagliardo | 2020 | Republic of the Congo | African Region | Lower middle income | before2022 | suspected cases | 39.00 | 5~99 | 11.00 | Central African clade | >5 |
| Reemergence of Human Monkeypox in Nigeria, 2017[42] | cross-sectional study | Yinka-Ogunleye, Adesola | 2018 | Nigeria | African Region | Lower middle income | before2022 | confirmed cases | 42.00 | 5~99 | 28.00 | West African clade | >5 |
| Investigation of an outbreak of monkeypox in an area occupied by armed groups, Central African Republic[18] | cross-sectional study | [E Kalthan](https://pubmed.ncbi.nlm.nih.gov/?sort=date&term=Kalthan+E&cauthor_id=29573840) | 2018 | Central African Republic | African Region | Low income | before2022 | all | 26.00 | 5~99 | 14.00 | Central African clade | >5 |
| Enhancing case definitions for surveillance of human monkeypox in the Democratic Republic of Congo[19] | cross-sectional study | Osadebe, Lynda | 2017 | Democratic Republic of the Congo | African Region | Low income | before2022 | confirmed cases | 333.00 | 100~500 | 178.00 | Central African clade | >5 |
| A Nosocomial Outbreak of Human Monkeypox in the Central African Republic[20] | case report | Nakoune, Emmanuel | 2017 | Central African Republic | African Region | Low income | before2022 | all | 10.00 | 5~99 | 5.00 | Central African clade | >5 |
| Varicella Coinfection in Patients with Active Monkeypox in the Democratic Republic of the Congo[43] | cross-sectional study | Hoff, N. A. | 2017 | Democratic Republic of the Congo | African Region | Low income | before2022 | confirmed cases | 785.00 | 500 | 480.00 | Central African clade | >5 |
| Extended human-to-human transmission during a monkeypox outbreak in the Democratic Republic of the Congo[22] | cross-sectional study | Nolen, L. D. | 2016 | Democratic Republic of the Congo | African Region | Low income | before2022 | confirmed cases | 20.00 | 5~99 | 12.00 | Central African clade | ≤5 |
| Human Monkeypox in the Kivus, a Conflict Region of the Democratic Republic of the Congo[44] | case report | McCollum, Andrea M. | 2015 | Democratic Republic of the Congo | African Region | Low income | before2022 | all | 6.00 | 5~99 | 4.00 | Central African clade | >5 |
| Cytokine modulation correlates with severity of monkeypox disease in humans[23] | others | Johnston, S. C. | 2015 | Democratic Republic of the Congo | African Region | Low income | before2022 | confirmed cases | 19.00 | 5~99 | 7.00 | Central African clade | ≤5 |
| Detection of human monkeypox in the Republic of the Congo following intensive community education[24] | case report | Reynolds, M. G. | 2013 | Republic of the Congo | African Region | Lower middle income | before2022 | suspected cases | 7.00 | 5~99 | 3.00 | Central African clade | >5 |
| Major increase in human monkeypox incidence 30 years after smallpox vaccination campaigns cease in the Democratic Republic of Congo[26] | cross-sectional study | Rimoin, Anne W. | 2010 | Democratic Republic of the Congo | African Region | Low income | before2022 | confirmed cases | 760.00 | 500 | 472.00 | Central African clade | >5 |
| Human monkeypox outbreak caused by novel virus belonging to Congo Basin clade, Sudan, 2005[45] | cross-sectional study | Formenty, P. | 2010 | Sudan | Eastern Mediterranean Region | Low income | before2022 | all | 19.00 | 5~99 | 9.00 | Central African clade | >5 |
| Monkeypox-induced immunity and failure of childhood smallpox vaccination to provide complete protection[46] | cross-sectional study | Karem, K. L. | 2007 | United States | Region of the Americas | High income | before2022 | all | 30.00 | 5~99 | 13.00 | West African clade | >5 |
| Occupational risks during a monkeypox outbreak, Wisconsin, 2003[27] | cross-sectional study | Croft, D. R. | 2007 | United States | Region of the Americas | High income | before2022 | all | 27.00 | 5~99 | 9.00 | West African clade | >5 |
| Clinical manifestations of human monkeypox influenced by route of infection[47] | cross-sectional study | Reynolds, M. G. | 2006 | United States | Region of the Americas | High income | before2022 | all | 47.00 | 5~99 | 22.00 | West African clade | >5 |
| Clinical characteristics of human monkeypox, and risk factors for severe disease[28] | cross-sectional study | Huhn, G. D. | 2005 | United States | Region of the Americas | High income | before2022 | confirmed cases | 34.00 | 5~99 | 18.00 | West African clade | >5 |
| Human monkeypox infection: a family cluster in the midwestern United States[29] | case report | Sejvar, James J. | 2004 | United States | Region of the Americas | High income | before2022 | confirmed cases | 3.00 | <5 | 1.00 | West African clade | >5 |
| The detection of monkeypox in humans in the Western Hemisphere[48] | cross-sectional study | Reed, K. D. | 2004 | United States | Region of the Americas | High income | before2022 | all | 11.00 | 5~99 | 5.00 | West African clade | >5 |
| Multistate outbreak of monkeypox--Illinois, Indiana, and Wisconsin, 2003[30] | case report | Centers for Disease Control and Prevention (CDC) | 2003 | United States | Region of the Americas | High income | before2022 | all | 53.00 | 5~99 | 29.00 | West African clade | >5 |
| Update: multistate outbreak of monkeypox--Illinois, Indiana, Kansas, Missouri, Ohio, and Wisconsin, 2003[31] | case report | Centers for Disease Control and Prevention (CDC) | 2003 | United States | Region of the Americas | High income | before2022 | all | 71.00 | 5~99 | 32.00 | West African clade | >5 |
| Outbreaks of disease suspected of being due to human monkeypox virus infection in the Democratic Republic of Congo in 2001[32] | cross-sectional study | Meyer, H. | 2002 | Democratic Republic of the Congo | African Region | Low income | before2022 | suspected cases | 10.00 | 5~99 | 8.00 | Central African clade | >5 |
| Outbreak of human monkeypox, Democratic Republic of Congo, 1996-1997[33] | cross-sectional study | Hutin, Y. J. F. | 2001 | Democratic Republic of the Congo | African Region | Low income | before2022 | all | 88.00 | 5~99 | 50.00 | Central African clade | >5 |
| Human monkeypox--Kasai Oriental, Zaire, 1996-1997[49] | case report | Centers for Disease, Control, Prevention | 1997 | Democratic Republic of the Congo | African Region | Low income | before2022 | possible cases | 92.00 | 5~99 | 51.00 | Central African clade | >5 |
| Clinico-epidemiological features of monkeypox patients with an animal or human source of infection[50] | cross-sectional study | Jezek, Z. | 1988 | Democratic Republic of the Congo | African Region | Low income | before2022 | confirmed cases | 338.00 | 100~500 | 182.00 | Central African clade | >5 |
| Human monkeypox: clinical features of 282 patients[51] | cross-sectional study | Jezek, Z. | 1987 | Democratic Republic of the Congo | African Region | Low income | before2022 | confirmed cases | 282.00 | 100~500 | 143.00 | Central African clade | >5 |
| Four generations of probable person-to-person transmission of human monkeypox[52] | case report | Jezek, Z. | 1986 | Democratic Republic of the Congo | African Region | Low income | before2022 | all | 5.00 | 5~99 | 4.00 | Central African clade | >5 |
| Human monkeypox, 1970-79[34] | cross-sectional study | Breman, J. G. | 1980 | Zaire (38), Liberia (4), Nigeria (3), Ivory Coast (1), and Sierra Leone(1) | others | others | before2022 | all | 47.00 | 5~99 | 26.00 | West African clade | >5 |
| Human monkeypox[53] | case report | Foster, S. O. | 1972 | Liberia, Nigeria, and Sierra Leone | others | others | before2022 | confirmed cases | 4.00 | <5 | 2.00 | West African clade | >5 |
| Simian smallpox (or monkey smallpox): Study of 8 cases observed at Impfondo hospital in Republic of Congo[35] | case report | Boumandouki, P. | 2007 | Republic of the Congo | African Region | Lower middle income | before2022 | all | 8.00 | 5~99 | 4.00 | Central African clade | >5 |
| Extended interhuman transmission of monkeypox in a hospital community in the Republic of the Congo, 2003[54] | case report | Learned, L. A. | 2005 | Republic of the Congo | African Region | Lower middle income | before2022 | confirmed cases | 3.00 | <5 | 2.00 | Central African clade | >5 |

**Notes：**Reported time refers to the year of reported cases (classified as 2022 group before 2022); Virus strain was classified as West African clade and Central African clade based on WHO; Quality Score was classified as >5 and ≤5; Study design includes case report, cross-sectional study, cohort study, case-control study, others (CDC website, promed mail etc.); Sample size includes >5 and 5~99, 100~500 three groups; Cases type: we extracted data reported the monkeypox cases including suspected cases, confirmed cases, probable cases and possible cases based on reported literature, if literature reported monkeypox cases including multiple cases types, we classified it as “all cases” group.

**Table S3: Information and indicators of original literature related to duration of symptoms (days) (Global. 2022)**

| Title | Study design | Author | Year | Country | WHO region | Income level | Reported time | Cases | Sample size1 | Sample size | Type of symptoms | average of duration of symptoms | Standard deviation | Virus | Quality scores |
| --- | --- | --- | --- | --- | --- | --- | --- | --- | --- | --- | --- | --- | --- | --- | --- |
| Family cluster of three cases of monkeypox imported from Nigeria to the United Kingdom, May 2021[55] | case report | Gemma Hobson | 2021 | The United Kingdom | European Region | High income | before2022 | confirmed cases | 3 | <5 | all | 17.666667 | 7.0945989 | West African clade | >5 |
| Clinical characteristics of human monkeypox, and risk factors for severe disease[28] | cross-sectional study | Huhn, G. D. | 2005 | United States | Region of the Americas | High income | before2022 | confirmed cases | 34 | 5~99 | rash | 12.7743 | 4.0644 | West African clade | >5 |
| Clinical characteristics of human monkeypox, and risk factors for severe disease[28] | cross-sectional study | Huhn, G. D. | 2005 | United States | Region of the Americas | High income | before2022 | confirmed cases | 34 | 5~99 | fever | 7.8894 | 2.6299 | West African clade | >5 |
| Human monkeypox: clinical features of 282 patients[51] | cross-sectional study | Jezek, Z. | 1987 | Democratic Republic of the Congo | African Region | Low income | before2022 | all cases | 282 | 100~500 | all | 16.3845 | 5.2708 | Central African clade | >5 |

**Notes：**Reported time refers to the year of reported cases (classified as 2022 group before 2022); Virus strain was classified as West African clade and Central African clade based on WHO; Quality Score was classified as >5 and ≤5; Study design includes case report, cross-sectional study, cohort study, case-control study, others (CDC website, promed mail etc.); Sample size includes >5 and 5~99, 100~500 three groups.

**Table S4: Information and indicators of original literature related to comorbidity rate (Global. 2022)**

| Title | Study design | Author | Year | WHO region | Income level | Reported time | Proportion of MSM | Average age | Percentage of men | Comorbidity type | Cases with comorbidity | Valid cases | Sample size | Virus strain | Quality score |
| --- | --- | --- | --- | --- | --- | --- | --- | --- | --- | --- | --- | --- | --- | --- | --- |
| Monkeypox Outbreak - Nine States, May 2022[56] | case report | Faisal S. Minhaj | 2022 | Region of the Americas | High income | 2022 | 95% | 18y~ |  | other immunodeficiency diseases | 3 | 17 | 5~99 | West African clade | >5 |
| Epidemiological, clinical and virological characteristics of four cases of monkeypox support transmission through sexual contact, Italy, May 2022[57] | case report | Andrea Antinori | 2022 | European Region | High income | 2022 | 100% | 18y~ | 75%~100% | HIV | 2 | 4 | <5 | West African clade | >5 |
| A Tale of Two Viruses: Coinfections of Monkeypox and Varicella Zoster Virus in the Democratic Republic of Congo[6] | cross-sectional study | Christine M. Hughes | 2021 | African Region | Low income | before 2022 |  | 0y~ |  | others | 134 | 534 | ≥500 | Central African clade | >5 |
| Clinical Course and Outcome of Human Monkeypox in Nigeria[7] | cross-sectional study | Dimie Ogoina | 2020 | African Region | Lower middle income | before 2022 |  | 18y~ | 75%~100% | HIV | 9 | 40 | 5~99 | West African clade | >5 |
| Descriptive epidemiology of monkeypox in Nigeria, September 2017–June 2019[39] | cross-sectional study | S. Akar | 2020 | African Region | Lower middle income | before 2022 |  | 18y~ | 50%~ | other immunodeficiency diseases | 6 | 165 | 100~499 | West African clade | ≤5 |
| Outbreak of human monkeypox in Nigeria in 2017–18: a clinical and epidemiological report[40] | cross-sectional study | Gemma Hobson | 2019 | African Region | Lower middle income | before 2022 |  | 18y~ | 50%~ | HIV | 4 | 7 | 5~99 | West African clade | >5 |
| The 2017 human monkeypox outbreak in Nigeria—Report of outbreak experience and response in the Niger Delta University Teaching Hospital, Bayelsa State, Nigeria[12] | cross-sectional study | Dimie Ogoina | 2019 | African Region | Lower middle income | before 2022 |  | 18y~ | 75%~100% | HIV | 2 | 18 | 5~99 | West African clade | >5 |
| Monkeypox 2022 outbreak: cases with exclusive genital lesions[58] | case report | Benjamin Davido | 2022 | European Region | High income | 2022 | 100% | 18y~ | 75%~100% | others | 1 | 2 | <5 | West African clade | >5 |
| Reemergence of Human Monkeypox in Nigeria, 2017[42] | cross-sectional study | Yinka-Ogunleye, Adesola | 2018 | African Region | Lower middle income | before 2022 |  | 18y~ | 50%~ | other immunodeficiency diseases | 1 | 42 | 5~99 | West African clade | >5 |
| Varicella Coinfection in Patients with Active Monkeypox in the Democratic Republic of the Congo[43] | cross-sectional study | Hoff, N. A. | 2017 | African Region | Low income | before 2022 |  | 0y~ | 50%~ | others | 151 | 782 | ≥500 | Central African clade | >5 |
| Extended human-to-human transmission during a monkeypox outbreak in the Democratic Republic of the Congo[22] | cross-sectional study | Nolen, L. D. | 2016 | African Region | Low income | before 2022 |  | 0y~ | 50%~ | others | 5 | 60 | 5~99 | Central African clade | ≤5 |
| Simple technique for in field samples collection in the cases of skin rash illness and subsequent PCR detection of orthopoxviruses and varicella zoster virus[59] | cross-sectional study | Dumont, C. | 2014 | African Region | Low income | before 2022 |  |  |  | others | 12 | 25 | 5~99 | Central African clade | ≤5 |
| Endemic human monkeypox, Democratic Republic of Congo, 2001-2004[60] | cross-sectional study | Rimoin, A. W. | 2007 | African Region | Low income | before 2022 |  | 0y~ | 25%~ | others | 1 | 51 | 5~99 | Central African clade | >5 |
| Human monkeypox infection: a family cluster in the midwestern United States[29] | case report | Sejvar, James J. | 2004 | Region of the Americas | High income | before 2022 |  | 18y~ | 25%~ | others | 1 | 3 | <5 | West African clade | >5 |
| Multistate outbreak of monkeypox--Illinois, Indiana, and Wisconsin, 2003[30] | case report | CDC | 2003 | Region of the Americas | High income | before 2022 |  | 18y~ | 50%~ | others | 1 | 14 | 5~99 | West African clade | >5 |
| Outbreaks of disease suspected of being due to human monkeypox virus infection in the Democratic Republic of Congo in 2001[32] | cross-sectional study | Meyer, H. | 2002 | African Region | Low income | before 2022 |  | 0y~ | 75%~100% | others | 7 | 16 | 5~99 | Central African clade | >5 |
| Monkeypox update (09) [37] | others | promed mail | 2022 | European Region | High income | 2022 | 95% | 18y~ | 75%~100% | HIV | 14 | 96 | 5~99 | West African clade | >5 |
| Monkeypox - Africa (06): Nigeria[37] | others | promed mail | 2018 | African Region | Lower middle income | before 2022 |  | 18y~ | 50%~ | other immunodeficiency diseases | 1 | 42 | 5~99 | West African clade | >5 |

**Notes：**Reported time refers to the year of reported cases (classified as 2022 group before 2022); Average age was classified as four groups: <18 year-child and teenager, 18 to 45 years- young people; 45 to 60 years old - middle-aged people; 60 and above – old people; Percentage of men refers to the proportion of male which is artificially divided into four categories -0~25%; 25-50%; 50-75%; 75-100%; 50-75%; 75-100%; Virus strain was classified as West African clade and Central African clade based on WHO; Quality Score was classified as >5 and ≤5; Study design includes case report, cross-sectional study, cohort study, case-control study, others (CDC website, promed mail etc.); Sample size includes >5 and≤5 two groups; Proportion of MSM refers to among the articles reporting homosexual transmission, the proportion of homosexual patients (based on available data) in the existing articles which can be divided into 100% and 95%; type of comorbidity include HIV, other immunodeficiency diseases, others.

**Table S5: Information and indicators of original literature related to case fatality rate (Global. 2022)**

| Title | Study design | Author | Year | WHO region | Income level | Reported time | Cases type | Cases | Death | Sample size | Reported sexual transmission | Average age | Percentage of men | Vaccination | Travel | Animal contact | Comorbidity | Virus strain | Quality score |
| --- | --- | --- | --- | --- | --- | --- | --- | --- | --- | --- | --- | --- | --- | --- | --- | --- | --- | --- | --- |
| Clinical Course and Outcome of Human Monkeypox in Nigeria[7] | cross-sectional study | Dimie Ogoina | 2020 | African Region | Lower middle income | before 2022 | Confirmed | 40 | 5 | 5~99 | No | 18y~ | 75%~100% | No |  |  | HIV | West African clade | >5 |
| Descriptive epidemiology of monkeypox in Nigeria, September 2017–June 2019[39] | cross-sectional study | S. Akar | 2020 | African Region | Lower middle income | before 2022 | Confirmed | 165 | 9 | 100~499 | No | 18y~ | 50%~ | No |  |  | other immunodeficiency disease | West African clade | ≤5 |
| Outbreak of human monkeypox in Nigeria in 2017–18: a clinical and epidemiological report[40] | cross-sectional study | Gemma Hobson | 2019 | African Region | Lower middle income | before 2022 | All | 276 | 7 | 100~499 | No | 18y~ | 50%~ | No |  | Yes | HIV | West African clade | >5 |
| Temporal and Spatial Dynamics of Monkeypox in Democratic Republic of Congo, 2000–2015[61] | cross-sectional study | Bien-Aime´ Makasa Mandja | 2019 | African Region | Low income | before 2022 | All | 19273 | 292 | ≥500 | No |  |  | No |  |  |  | Central African clade | ≤5 |
| Reemergence of Human Monkeypox in Nigeria, 2017[42] | cross-sectional study | Yinka-Ogunleye, Adesola | 2018 | African Region | Lower middle income | before 2022 | Confirmed | 42 | 1 | 5~99 | No | 18y~ | 50%~ | No |  |  | other immunodeficiency disease | West African clade | >5 |
| Investigation of an outbreak of monkeypox in an area occupied by armed groups, Central African Republic[18] | cross-sectional study | [E Kalthan](https://pubmed.ncbi.nlm.nih.gov/?sort=date&term=Kalthan+E&cauthor_id=29573840) | 2018 | African Region | Low income | before 2022 | All | 26 | 2 | 5~99 | No | 18y~ | 50%~ | Yes |  | Yes |  | Central African clade | >5 |
| Notes from the Field: Responding to an Outbreak of Monkeypox Using the One Health Approach - Nigeria, 2017-2018[62] | case report | Eteng, Womi-Eteng | 2018 | African Region | Lower middle income | before 2022 | Confirmed | 89 | 6 | 5~99 | No |  |  | No |  |  |  | West African clade | >5 |
| Strengthening of Surveillance during Monkeypox Outbreak, Republic of the Congo, 2017[63] | case report | Doshi, R. H. | 2018 | African Region | Lower middle income | before 2022 | All | 81 | 6 | 5~99 | No |  |  | No |  |  |  | Central African clade | ≤5 |
| A Nosocomial Outbreak of Human Monkeypox in the Central African Republic[20] | case report | Nakoune, Emmanuel | 2017 | African Region | Low income | before 2022 | All | 10 | 2 | 5~99 | No | 18y~ | 50%~ | No | Yes | Yes |  | Central African clade | >5 |
| Extended human-to-human transmission during a monkeypox outbreak in the Democratic Republic of the Congo[22] | cross-sectional study | Nolen, L. D. | 2016 | African Region | Low income | before 2022 | All | 104 | 10 | 5~99 | No | 0y~ | 50%~ | Yes |  |  | others | Central African clade | ≤5 |
| Human monkeypox disease surveillance and time trends in The Democratic Republic of Congo, 2001-2013[64] | others | Hoff, N. | 2014 | African Region | Low income | before 2022 | All | 19437 | 326 | ≥500 | No |  |  | No |  |  |  | Central African clade |  |
| Detection of human monkeypox in the Republic of the Congo following intensive community education[24] | case report | Reynolds, M. G. | 2013 | African Region | Lower middle income | before 2022 | All | 10 | 1 | 5~99 | No | 0y~ | 25%~ | No |  | Yes |  | Central African clade | >5 |
| A tale of two clades: monkeypox viruses[65] | cross-sectional study | Likos, A. M. | 2005 | African Region | Low income | before 2022 | All | 127 | 3 | 100~499 | No | 18y~ |  | Yes |  |  |  | Central African clade | >5 |
| Outbreaks of disease suspected of being due to human monkeypox virus infection in the Democratic Republic of Congo in 2001[32] | cross-sectional study | Meyer, H. | 2002 | African Region | Low income | before 2022 | Confirmed | 23 | 5 | 5~99 | No | 0y~ | 75%~100% | No |  | Yes | others | Central African clade | >5 |
| Human monkeypox--Kasai Oriental, Zaire, 1996-1997[49] | case report | Centers for Disease, Control, Prevention | 1997 | African Region | Low income | before 2022 | All | 92 | 3 | 5~99 | No | 18y~ | 50%~ | Yes |  |  |  | Central African clade | >5 |
| Clinico-epidemiological features of monkeypox patients with an animal or human source of infection[50] | cross-sectional study | Jezek, Z. | 1988 | African Region | Low income | before 2022 | All | 338 | 33 | 100~499 | No | 0y~ | 50%~ | Yes |  | Yes |  | Central African clade | >5 |
| Human monkeypox: clinical features of 282 patients[51] | cross-sectional study | Jezek, Z. | 1987 | African Region | Low income | before 2022 | Confirmed | 270 | 27 | 100~499 | No | 18y~ | 50%~ | Yes |  |  |  | Central African clade | >5 |
| Human monkeypox, 1970-79[34] | cross-sectional study | Breman, J. G. | 1980 |  |  | before 2022 | All | 47 | 8 | 5~99 | No | 0y~ | 50%~ | Yes | No | Yes |  |  | >5 |
| Extended interhuman transmission of monkeypox in a hospital community in the Republic of the Congo, 2003[54] | case report | Learned, L. A. | 2005 | African Region | Lower middle income | before 2022 | All | 11 | 1 | 5~99 | No | 0y~ | 50%~ | No |  | Yes |  | West African clade | >5 |
| From the Centers for Disease Control and Prevention. Human monkeypox--Kasai Oriental, Democratic Republic of Congo, February 1996-October 1997[66] | others | No authors listed | 1998 | African Region | Low income | before 2022 | All | 344 | 5 | 100~499 | No | 0y~ |  | No | Yes |  |  | Central African clade | >5 |
| Human monkeypox in Kasai Oriental, Zaire (1996-1997) [67] | others | Mwanbal, P. T. | 1997 | African Region | Low income | before 2022 | All | 92 | 3 | 5~99 | No | 18y~ | 50%~ | Yes |  |  |  | Central African clade | ≤5 |
| Re-emergence of human monkeypox in Zaire in 1996. Monkeypox Epidemiologic Working Group[68] | others | Mukinda, V. B. | 1997 | African Region | Low income | before 2022 | All | 71 | 6 | 5~99 | No |  |  | No |  | Yes |  | Central African clade | >5 |
| Multi-country monkeypox outbreak: situation update 10 June 2022[69] | case report | WHO | 2022 |  |  | 2022 | All | 1536 | 72 | ≥500 | No |  |  | No |  |  |  |  |  |
| Multi-country monkeypox outbreak: situation update 10 June 2022[69] | case report | WHO | 2022 | African Region | Lower middle income | 2022 | All | 28 | 2 | 5~99 | No |  |  | No |  |  |  |  |  |
| Multi-country monkeypox outbreak: situation update 10 June 2022[69] | case report | WHO | 2022 | African Region | Low income | 2022 | All | 17 | 2 | 5~99 | No |  |  | No |  |  |  | Central African clade |  |
| Multi-country monkeypox outbreak: situation update 10 June 2022[69] | case report | WHO | 2022 | African Region | Lower middle income | 2022 | All | 7 | 3 | 5~99 | No |  |  | No |  |  |  | Central African clade |  |
| Multi-country monkeypox outbreak: situation update 10 June 2022[69] | case report | WHO | 2022 | African Region | Low income | 2022 | All | 1356 | 64 | ≥500 | No |  |  | No |  |  |  | Central African clade |  |
| Multi-country monkeypox outbreak: situation update 10 June 2022[69] | case report | WHO | 2022 | African Region | Lower middle income | 2022 | Confirmed | 31 | 1 | 5~99 | No |  |  | No |  |  |  | West African clade |  |
| Monkeypox[70] | case report | African CDC | 2022 | African Region | Low income | before 2022 | All | 26 | 1 | 5~99 | No |  |  | No |  |  |  | Central African clade |  |
| An Update of Monkeypox Outbreak in Nigeria[71] | case report | Nigeria CDC | 2022 | African Region | Lower middle income | 2022 | Confirmed | 36 | 1 | 5~99 | No |  |  | No |  |  |  | West African clade | >5 |
| An Update of Monkeypox Outbreak in Nigeria[71] | case report | Nigeria CDC | 2022 | African Region | Lower middle income | before 2022 | Confirmed | 88 | 5 | 5~99 | No |  |  | No |  |  |  | West African clade |  |
| An Update of Monkeypox Outbreak in Nigeria[71] | case report | Nigeria CDC | 2022 | African Region | Lower middle income | before 2022 | Confirmed | 49 | 3 | 5~99 | No |  |  | No |  |  |  | West African clade |  |
| An Update of Monkeypox Outbreak in Nigeria[71] | case report | Nigeria CDC | 2022 | African Region | Lower middle income | 2022 | Confirmed | 262 | 9 | 100~499 | No | 18y~ | 50%~ | No |  |  |  | West African clade |  |
| Monkeypox update (07) [37] | others | promed mail | 2022 | African Region | Lower middle income | 2022 | All | 25 | 9 | 5~99 | No |  |  | No | Yes |  |  |  | ≤5 |
| Monkeypox - Africa (09): Congo DR[37] | others | promed mail | 2021 | African Region | Low income | before 2022 | All | 2764 | 72 | ≥500 | No |  |  | No |  |  |  | Central African clade | ≤5 |
| Monkeypox - Africa (09): Congo DR[37] | others | promed mail | 2021 | African Region | Low income | before 2022 | All | 6257 | 229 | ≥500 | No |  |  | No |  |  |  | Central African clade | ≤5 |
| Monkeypox - Africa (09): Congo DR[37] | others | promed mail | 2021 | African Region | Low income | before 2022 | All | 5288 | 107 | ≥500 | No |  |  | No |  |  |  | Central African clade | ≤5 |
| Monkeypox - Africa (08): Congo DR, WHO[37] | others | promed mail | 2020 | African Region | Low income | before 2022 | All | 4594 | 171 | ≥500 | No | 0y~ |  | No |  |  |  | Central African clade | ≤5 |
| Monkeypox - Africa: Central African Republic[37] | others | promed mail | 2019 | African Region | Low income | before 2022 | Confirmed | 25 | 2 | 5~99 | No |  |  | No |  |  |  | Central African clade | ≤5 |
| Monkeypox - Africa (06): Nigeria[37] | others | promed mail | 2018 | African Region | Lower middle income | before 2022 | Confirmed | 42 | 1 | 5~99 | No | 18y~ | 50%~ | No |  |  | other immunodeficiency disease | West African clade | >5 |
| Monkeypox - Africa: Congo DR, Central African Republic[37] | others | promed mail | 2017 | African Region | Low income | before 2022 | All | 78 | 4 | 5~99 | No |  |  | No |  |  |  | Central African clade | ≤5 |
| Monkeypox - Central African Republic (04): (HK) WHO[37] | others | promed mail | 2016 | African Region | Low income | before 2022 | All | 26 | 1 | 5~99 | No |  |  | No |  |  |  | Central African clade | >5 |
| Monkeypox - Congo DR (02): (BU) [37] | others | promed mail | 2016 | African Region | Low income | before 2022 | All | 51 | 2 | 5~99 | No |  |  | No |  |  |  | Central African clade | ≤5 |
| Monkeypox - Congo DR (Bokungu) [37] | others | promed mail | 2008 | African Region | Low income | before 2022 | All | 39 | 3 | 5~99 | No | 0y~ |  | No |  |  |  | Central African clade | ≤5 |
| Monkeypox - Congo, Dem. Rep. (Mbuji-Mayi): 1999[37] | others | promed mail | 2000 | African Region | Low income | before 2022 | All | 70 | 6 | 5~99 | No |  |  | No |  |  |  | Central African clade | ≤5 |
| First appearance of monkey pox in human beings in Gabon[72] | case report | Meyer, A. | 1991 | African Region | Upper middle income | before 2022 | All | 4 | 2 | <5 | No |  |  | No |  |  |  | Central African clade | ≤5 |

**Notes：**Reported time refers to the year of reported cases (classified as 2022 group before 2022); Average age was classified as four groups: <18 year-child and teenager, 18 to 45 years- young people; 45 to 60 years old - middle-aged people; 60 and above – old people; Percentage of men refers to the proportion of male which is artificially divided into four categories -0~25%; 25-50%; 50-75%; 75-100%; 50-75%; 75-100%; Virus strain was classified as West African clade and Central African clade based on WHO; Quality Score was classified as >5 and ≤5; Study design includes case report、cross-sectional study、cohort study、case-control study、others (CDC website, promed mail etc.); Sample size includes >5 and≤5 two groups; Cases type: we extracted data reported the monkeypox cases including suspected cases, confirmed cases, probable cases and possible cases based on reported literature, if literature reported monkeypox cases including multiple cases types, we classified it as “all cases” group; Reported sexual transmission included Yes (articles reporting sexual transmission) and NO (articles that do not report sexual transmission); Vaccination included Yes (articles reporting that people have been vaccinated) and NO (articles that did not report vaccination) groups; Travel included Yes (articles reporting travel history) and NO group (articles did not report travel history); Contact with animals included Yes (articles reporting a history of animal contact) and ;NO group (articles did not report history of animal contact); Other diseases refers to the types of comorbidity, which are divided into HIV, Immunodeficiency, varicella-zoster virus (VZV) and Others.

**Table S6: Information and indicators of original literature related to secondary attack rate (Global. 2022)**

| Title | Study design | Author | Year | WHO region | Income level | Reported time | Secondary cases | Contacts | Sample size | Average age | Virus strain |
| --- | --- | --- | --- | --- | --- | --- | --- | --- | --- | --- | --- |
| Family cluster of three cases of monkeypox imported from Nigeria to the United Kingdom, May 2021[55] | case report | Gemma Hobson | 2021 | European Region | High income | before 2022 | 2 | 38 | 5~99 |  | West African clade |
| Human-to-Human Transmission of Monkeypox Virus,United Kingdom, October 2018[73] | case report | Aisling Vaughan | 2020 | European Region | High income | before 2022 | 1 | 288 | 100~499 |  | West African clade |
| Use of Surveillance Outbreak Response Management and Analysis System for Human Monkeypox Outbreak, Nigeria, 2017-2019[74] | case report | Bernard C. Silenou | 2020 | African Region | Lower middle income | before 2022 | 12 | 167 | 100~499 |  | West African clade |
| Human monkeypox transmission dynamics thirty years after smallpox eradication in the Sankuru district, democratic republic of Congo[75] | others | McMullen, C. L. | 2015 | African Region | Low income | before 2022 | 20 | 293 | 100~499 |  | Central African clade |
| Human monkeypox: secondary attack rates[76] | cross-sectional study | Jezek, Z. | 1988 | African Region | Low income | before 2022 | 69 | 2278 | ≥500 | 0y~ | Central African clade |
| The transmission potential of monkeypox virus in human populations[77] | cross-sectional study | Fine, P. E. | 1988 | African Region | Low income | before 2022 | 47 | 1573 | ≥500 |  | Central African clade |
| Human monkeypox: A study of 2,510 contacts of 214 patients[78] | cross-sectional study | Jezek, Z. | 1986 | African Region | Low income | before 2022 | 62 | 2510 | ≥500 | 0y~ | Central African clade |
| From the Centers for Disease Control and Prevention. Human monkeypox--Kasai Oriental, Democratic Republic of Congo, February 1996-October 1997[66] | others | No authors listed | 1998 | African Region | Low income | before 2022 | 325 | 4062 | ≥500 | 0y~ | Central African clade |

**Notes：**Reported time refers to the year of reported cases (classified as 2022 group before 2022); Average age was classified as four groups: <18 year-child and teenager, 18 to 45 years- young people; 45 to 60 years old - middle-aged people; 60 and above – old people; Virus strain was classified as West African clade and Central African clade based on WHO; Quality Score was classified as >5 and ≤5; Study design includes case report、cross-sectional study、cohort study、case-control study、others (CDC website, promed mail etc.).

**Table S7: Information and indicators of original literature related to average of incubation period (days)** **(Global. 2022)**

| Title | Study design | Author | Year | Country | WHO region | Income level | Reported time | Cases | Sample size1 | Sample size | Average of incubation period | standard deviation | Virus | Quality scores |
| --- | --- | --- | --- | --- | --- | --- | --- | --- | --- | --- | --- | --- | --- | --- |
| Extended human-to-human transmission during a monkeypox outbreak in the Democratic Republic of the Congo[22] | cross-sectional study | Nolen, L. D. | 2016 | Democratic Republic of the Congo | African Region | Low income | before2022 | all cases | 16 | 5~99 | 9.1667 | 2.5441 | Central African clade | ≤5 |
| Clinical characteristics of human monkeypox, and risk factors for severe disease[28] | cross-sectional study | Huhn, G. D. | 2005 | United States | Region of the Americas | High income | before2022 | confirmed cases | 29 | 5~99 | 13.7848 | 5.4681 | West African clade | >5 |
| The detection of monkeypox in humans in the Western Hemisphere[48] | cross-sectional study | Reed, K. D. | 2004 | United States | Region of the Americas | High income | before2022 | all cases | 11 | 5~99 | 14.6016 | 6.2766 | West African clade | >5 |
| Update: multistate outbreak of monkeypox--Illinois, Indiana, Kansas, Missouri, Ohio, and Wisconsin, 2003[31] | case report | Centers for Disease Control and Prevention (CDC) | 2003 | United States | Region of the Americas | High income | before2022 | all cases | 35 | 5~99 | 14.8446 | 23.1894 | West African clade | >5 |
| Transmission of monkeypox among persons exposed to infected prairie dogs in Indiana in 2003[36] | cross-sectional study | Kile, J. C. | 2005 | United States | Region of the Americas | High income | before2022 | all cases | 5 | 5~99 | 6 | 4.2381 | West African clade | >5 |

**Notes：**Reported time refers to the year of reported cases (classified as 2022 group before 2022); Virus strain was classified as West African clade and Central African clade based on WHO; Quality Score was classified as >5 and ≤5; Study design includes case report, cross-sectional study, cohort study, case-control study, others (CDC website, promed mail etc.);.

**Table S8: Information and indicators of original literature related to proportion of animal contact history (Global. 2022)**

| Title | Study design | Author | Year | WHO region | Income level | Reported time | Average age | Percentage of men | Vaccination | Animal contact cases | Valid cases | Sample size | Types of animal | Virus strain | Quality score |
| --- | --- | --- | --- | --- | --- | --- | --- | --- | --- | --- | --- | --- | --- | --- | --- |
| Clinical and Epidemiological Findings from Enhanced Monkeypox Surveillance in Tshuapa Province, Democratic Republic of the Congo During 2011–2015[5] | cross-sectional study | Erin R. Whitehouse | 2021 | African Region | Low income | before 2022 | 0y~ | 50%~ | Yes | 199 | 324 | 100~499 | non-human primates | Central African clade | >5 |
| Clinical and Epidemiological Findings from Enhanced Monkeypox Surveillance in Tshuapa Province, Democratic Republic of the Congo During 2011–2015[5] | cross-sectional study | Erin R. Whitehouse | 2021 | African Region | Low income | before 2022 | 0y~ | 50%~ | Yes | 81 | 324 | 100~499 | rodents | Central African clade | >5 |
| Clinical and Epidemiological Findings from Enhanced Monkeypox Surveillance in Tshuapa Province, Democratic Republic of the Congo During 2011–2015[5] | cross-sectional study | Erin R. Whitehouse | 2021 | African Region | Low income | before 2022 | 0y~ | 50%~ | Yes | 44 | 324 | 100~499 | others | Central African clade | >5 |
| Monkeypox Rash Severity and Animal Exposures in the Democratic Republic of the Congo[9] | cross-sectional study | Reena H. Doshi | 2019 | African Region | Low income | before 2022 | 0y~ | 50%~ | Yes | 756 | 831 | ≥500 | rodents | Central African clade | >5 |
| Monkeypox Rash Severity and Animal Exposures in the Democratic Republic of the Congo[9] | cross-sectional study | Reena H. Doshi | 2019 | African Region | Low income | before 2022 | 0y~ | 50%~ | Yes | 640 | 831 | ≥500 | non-human primates | Central African clade | >5 |
| Outbreak of human monkeypox in Nigeria in 2017–18: a clinical and epidemiological report[40] | cross-sectional study | Gemma Hobson | 2019 | African Region | Lower middle income | before 2022 | 18y~ | 50%~ | No | 10 | 118 | 100~499 |  | West African clade | >5 |
| Intrafamily Transmission of Monkeypox Virus, Central African Republic, 2018[14] | cross-sectional study | Camille Besombes | 2019 | African Region | Low income | before 2022 | 0y~ | 0%~ | No | 1 | 6 | 5~99 |  | Central African clade | >5 |
| Exportation of Monkeypox Virus From the African Continent[15] | cross-sectional study | Matthew R Mauldin | 2022 | African Region | Lower middle income | before 2022 | 18y~ | 75%~100% | No | 3 | 6 | 5~99 |  | West African clade | ≤5 |
| Vaccinating against monkeypox in the Democratic Republic of the Congo[79] | cross-sectional study | Petersen, Brett W. | 2019 | African Region | Low income | before 2022 | 18y~ | 75%~100% | Yes | 9 | 699 | ≥500 |  | Central African clade | ≤5 |
| Investigation of an outbreak of monkeypox in an area occupied by armed groups, Central African Republic[18] | cross-sectional study | [E Kalthan](https://pubmed.ncbi.nlm.nih.gov/?sort=date&term=Kalthan+E&cauthor_id=29573840) | 2018 | African Region | Low income | before 2022 | 18y~ | 50%~ | Yes | 1 | 26 | 5~99 |  | Central African clade | >5 |
| A Nosocomial Outbreak of Human Monkeypox in the Central African Republic[20] | case report | Nakoune, Emmanuel | 2017 | African Region | Low income | before 2022 | 18y~ | 50%~ | No | 1 | 10 | 5~99 |  | Central African clade | >5 |
| Human Monkeypox in the Kivus, a Conflict Region of the Democratic Republic of the Congo[44] | case report | McCollum, Andrea M. | 2015 | African Region | Low income | before 2022 | 0y~ | 50%~ | No | 1 | 6 | 5~99 | consume bush meat | Central African clade | >5 |
| Cytokine modulation correlates with severity of monkeypox disease in humans[23] | others | Johnston, S. C. | 2015 | African Region | Low income | before 2022 | 0y~ | 25%~ | No | 3 | 19 | 5~99 |  | Central African clade | ≤5 |
| Genomic variability of monkeypox virus among humans, Democratic Republic of the Congo[80] | cross-sectional study | Kugelman, J. R. | 2014 | African Region | Low income | before 2022 | 0y~ | 50%~ | No | 20 | 760 | ≥500 |  | Central African clade | >5 |
| Risk factors associated with human monkeypox in the democratic republic of Congo[81] | others | Hoff, N. | 2014 | African Region | Low income | before 2022 |  |  | No | 252 | 390 | 100~499 |  | Central African clade |  |
| Detection of human monkeypox in the Republic of the Congo following intensive community education[24] | case report | Reynolds, M. G. | 2013 | African Region | Lower middle income | before 2022 | 0y~ | 25%~ | No | 1 | 10 | 5~99 |  | Central African clade | >5 |
| Clinical manifestations of human monkeypox influenced by route of infection[47] | cross-sectional study | Reynolds, M. G. | 2006 | Region of the Americas | High income | before 2022 |  | 25%~ | No | 27 | 47 | 5~99 | others | West African clade | >5 |
| Clinical characteristics of human monkeypox, and risk factors for severe disease[28] | cross-sectional study | Huhn, G. D. | 2005 | Region of the Americas | High income | before 2022 | 18y~ | 50%~ | Yes | 19 | 34 | 5~99 | others | West African clade | >5 |
| Update: multistate outbreak of monkeypox--Illinois, Indiana, Kansas, Missouri, Ohio, and Wisconsin, 2003[31] | case report | CDC | 2003 | Region of the Americas | High income | before 2022 | 18y~ | 25%~ | Yes | 35 | 71 | 5~99 |  | West African clade | >5 |
| Clinico-epidemiological features of monkeypox patients with an animal or human source of infection[50] | cross-sectional study | Jezek, Z. | 1988 | African Region | Low income | before 2022 | 0y~ | 50%~ | Yes | 245 | 338 | 100~499 |  | Central African clade | >5 |
| Human monkeypox: disease pattern, incidence and attack rates in a rural area of northern Zaire[82] | cross-sectional study | Jezek, Z. | 1988 | African Region | Low income | before 2022 | 0y~ |  | Yes | 70 | 91 | 5~99 |  | Central African clade | ≤5 |
| Monkeypox, human, prairie dogs - USA (15) [37] | others | promed mail | 2003 | Region of the Americas | High income | before 2022 | 18y~ | 25%~ | No | 35 | 71 | 5~99 |  | West African clade | >5 |
| Human Monkeypox in Sierra Leone after 44-Year Absence of Reported Cases[10] | case report | Mary G. Reynolds | 2019 | African Region | Low income | before 2022 | 0y~ | 75%~100% | No | 1 | 2 | <5 |  | West African clade | >5 |
| Two cases of monkeypox imported to the United Kingdom, September 2018[83] | case report | Vaughan, A. | 2018 | European Region | High income | before 2022 |  | 75%~100% | No | 1 | 2 | <5 | consume bush meat | West African clade | >5 |
| Four generations of probable person-to-person transmission of human monkeypox[52] | case report | Jezek, Z. | 1986 | African Region | Low income | before 2022 | 0y~ | 75%~100% | Yes | 1 | 5 | <5 |  | Central African clade | >5 |

**Notes：**Reported time refers to the year of reported cases (classified as 2022 group before 2022); Average age was classified as four groups: <18 year-child and teenager, 18 to 45 years- young people; 45 to 60 years old - middle-aged people; 60 and above – old people; Percentage of men refers to the proportion of male which is artificially divided into four categories -0~25%; 25-50%; 50-75%; 75-100%; 50-75%; 75-100%; Virus strain was classified as West African clade and Central African clade based on WHO; Quality Score was classified as >5 and ≤5; Study design includes case report、cross-sectional study、cohort study、case-control study、others (CDC website, promed mail etc.); Sample size includes >5 and≤5 two groups; Type of animals included non-human primates, rodents, consume bush meat and others; Vaccination included Yes (articles reporting that people have been vaccinated) and NO (articles that did not report vaccination) groups.

**Table S9: Information and indicators of original literature related to proportion of proportion of travel history (Global. 2022)**

| Title | Study design | Author | Year | WHO region | Income level | Reported time | Secondary cases | Sexual transmition | Average age | Percentage of men | Vaccination | Travel-related cases | Valid cases | Sample size | Traveling area | Virus strain | Quality score |
| --- | --- | --- | --- | --- | --- | --- | --- | --- | --- | --- | --- | --- | --- | --- | --- | --- | --- |
| Community transmission of monkeypox in the United Kingdom, April to May 2022[3] | case report | Roberto Vivancos | 2022 | European Region | High income | 2022 | No | Yes | 18y~ | 75%~100% | No | 1 | 82 | 5~99 |  | West African clade | >5 |
| Clinical features and management of human monkeypox: a retrospective observational study in the UK[38] | cross-sectional study | Hugh Adler | 2022 | European Region | High income | before 2022 | Yes | No |  | 50%~ | Yes | 4 | 7 | 5~99 | Nigeria | West African clade | >5 |
| Exportation of Monkeypox Virus From the African Continent[15] | cross-sectional study | Matthew R Mauldin | 2022 | African Region | Lower middle income | before 2022 | Yes | No | 18y~ | 75%~100% | No | 4 | 6 | 5~99 | Nigeria | West African clade | ≤5 |
| Letter to the editor: multiple introductions of MPX in Italy from different geographic areas[16] | others | Federica Ferraro | 2022 | European Region | High income | 2022 |  | Yes | 18y~ | 75%~100% | No | 23 | 29 | 5~99 | Europe and West Africa | West African clade | >5 |
| Human Monkeypox in the Kivus, a Conflict Region of the Democratic Republic of the Congo[44] | case report | McCollum, Andrea M. | 2015 | African Region | Low income | before 2022 |  | No | 0y~ | 50%~ | Yes | 1 | 6 | 5~99 |  | Central African clade | >5 |
| From the Centers for Disease Control and Prevention. Human monkeypox--Kasai Oriental, Democratic Republic of Congo, February 1996-October 1997[66] | others | No authors listed | 1998 | African Region | Low income | before 2022 | Yes | No |  |  | No | 147 | 419 | 100~499 |  | Central African clade | >5 |
| Monkeypox update (12) [37] | others | promed mail | 2022 | Region of the Americas | Upper middle income | 2022 |  | No | 18y~ | 75%~100% | No | 1 | 7 | 5~99 | Europe | West African clade | >5 |
| Monkeypox update (12) [37] | others | promed mail | 2022 | European Region | High income | 2022 |  | Yes | 18y~ | 75%~100% | No | 75 | 152 | 100~499 |  | West African clade | ≤5 |
| Monkeypox update (09) [37] | others | promed mail | 2022 | European Region | High income | 2022 | Yes | Yes | 18y~ | 75%~100% | Yes | 4 | 27 | 5~99 | Europe and Brazl | West African clade | >5 |
| Monkeypox - UK (03): local transmission[37] | others | promed mail | 2022 | European Region | High income | 2022 |  | Yes |  |  | No | 1 | 7 | 5~99 | Nigeria | West African clade | ≤5 |
| Family cluster of three cases of monkeypox imported from Nigeria to the United Kingdom, May 2021[55] | case report | Gemma Hobson | 2021 | European Region | High income | before 2022 | Yes | No |  |  | No | 1 | 3 | <5 | Nigeria | West African clade | >5 |
| Monkeypox update (09) [37] | others | promed mail | 2022 | Region of the Americas | High income | 2022 | Yes | No |  |  | No | 1 | 3 | <5 | Europe | West African clade | ≤5 |
| Monkeypox - UK: ex Africa[37] | others | promed mail | 2021 | European Region | High income | before 2022 | Yes | No |  |  | No | 1 | 2 | <5 | Nigeria | West African clade | >5 |

**Notes：**Reported time refers to the year of reported cases (classified as 2022 group before 2022); Average age was classified as four groups: <18 year-child and teenager, 18 to 45 years- young people; 45 to 60 years old - middle-aged people; 60 and above – old people; Percentage of men refers to the proportion of male which is artificially divided into four categories -0~25%; 25-50%; 50-75%; 75-100%; 50-75%; 75-100%; Virus strain was classified as West African clade and Central African clade based on WHO; Quality Score was classified as >5 and ≤5; Study design includes case report、cross-sectional study、cohort study、case-control study、others (CDC website, promed mail etc.); Sample size includes >5 and≤5 two groups; Vaccination included Yes (articles reporting that people have been vaccinated) and NO (articles that did not report vaccination) groups; Second-generation cases: Yes (Second-generation case were reported) and NO (second-generation cases were not report); Sexual transmission: Yes (articles reported situation of sexual transmission) and NO (articles did not report situation of sexual transmission).

**Table S10: Information and indicators of original literature related to the proportion of homosexual (Global. 2022)**

| Title | Study design | Author | Year | WHO region | Income level | Reported time | MSM | Valid cases | Sample size | Average age | Percentage of men | Travel | Virus strain | Quality score |
| --- | --- | --- | --- | --- | --- | --- | --- | --- | --- | --- | --- | --- | --- | --- |
| Community transmission of monkeypox in the United Kingdom, April to May 2022[3] | case report | Roberto Vivancos | 2022 | European Region | High income | 2022 | 66 | 82 | 5~99 | 18y~ | 75%~100% | Yes | West African clade | >5 |
| Ongoing monkeypox virus outbreak, Portugal, 29 April to 23 May 2022[4] | case report | Mariana Perez Duque | 2022 | European Region | High income | 2022 | 18 | 19 | 5~99 | 18y~ | 75%~100% | Yes | West African clade | >5 |
| Monkeypox Outbreak - Nine States, May 2022[56] | case report | Faisal S. Minhaj | 2022 | Region of the Americas | High income | 2022 | 16 | 17 | 5~99 | 18y~ |  | Yes | West African clade | >5 |
| Seven monkeypox cases are confirmed in England[84] | case report | Elisabeth Mahase | 2022 | European Region | High income | 2022 | 4 | 7 | 5~99 |  |  |  | West African clade | ≤5 |
| Letter to the editor: multiple introductions of MPX in Italy from different geographic areas[16] | others | Federica Ferraro | 2022 | European Region | High income | 2022 | 16 | 18 | 5~99 | 18y~ | 75%~100% | Yes | West African clade | >5 |
| Monkeypox update (12) [37] | others | promed mail | 2022 | European Region | High income | 2022 | 151 | 152 | 100~499 | 18y~ | 75%~100% | Yes | West African clade | ≤5 |
| Monkeypox update (09) [37] | others | promed mail | 2022 | European Region | High income | 2022 | 111 | 199 | 100~499 | 18y~ | 75%~100% | Yes | West African clade | >5 |
| Monkeypox update (05) [37] | others | promed mail | 2022 | European Region | High income | 2022 | 6 | 9 | 5~99 |  |  |  | West African clade | ≤5 |
| Monkeypox - UK (03): local transmission[37] | others | promed mail | 2022 | European Region | High income | 2022 | 4 | 7 | 5~99 |  |  | Yes | West African clade | ≤5 |

**Notes：**Reported time refers to the year of reported cases (classified as 2022 group before 2022); Average age was classified as four groups: <18 year-child and teenager, 18 to 45 years- young people; 45 to 60 years old - middle-aged people; 60 and above – old people; Percentage of men refers to the proportion of male which is artificially divided into four categories -0~25%; 25-50%; 50-75%; 75-100%; 50-75%; 75-100%; Virus strain was classified as West African clade and Central African clade based on WHO; Quality Score was classified as >5 and ≤5; Study design includes case report, cross-sectional study, cohort study, case-control study and others (CDC website, promed mail etc.); Sample size includes >5 and≤5 two groups; Travel included Yes (articles reporting travel history) and NO group (articles did not report travel history).

**Table S11: The subgroup analysis for demographic characteristics estimates including average age and proportion of male patients for monkeypox cases (Global. 2022)**

|  | Average of age (years) | | | | |  |  |  | Proportion of male patients (%) | | | |
| --- | --- | --- | --- | --- | --- | --- | --- | --- | --- | --- | --- | --- |
|  | No. studies | Sample size | Effect | Lower limit | Higher limit |  | No. studies | Sample size | No. male patients | Effect | Lower limit | Higher limit |
| **All** | 36 | 3513 | 21.05 | 17.20 | 24.90 |  | 43 | 2872/4964 | 57.9% | 53.8% | 62.1% | 43 |
| **Study design** |  |  |  |  |  |  |  |  |  |  |  |  |
| Case report | 12 | 276 | 20.20 | 13.42 | 26.98 |  | 12 | 269 | 141 | 52.7% | 46.8% | 58.6% |
| Cross-sectional study | 20 | 3160 | 20.14 | 14.85 | 25.43 |  | 29 | 4647 | 2696 | 57.9% | 54.2% | 61.5% |
| Others | 2 | 77 | 25.51 | 13.77 | 37.26 |  | 2 | 48 | 35 | 67.6% | 9.1% | 126.1% |
| **WHO region** |  |  |  |  |  |  |  |  |  |  |  |  |
| African Region | 24 | 3108 | 18.01 | 13.88 | 22.14 |  | 30 | 4582 | 2674 | 59.4% | 55.7% | 63.0% |
| Region of the Americas | 6 | 191 | 25.75 | 15.60 | 35.91 |  | 8 | 276 | 129 | 46.6% | 40.7% | 52.4% |
| European Region | 4 | 165 | 35.72 | 26.92 | 44.52 |  | 2 | 36 | 32 | 81.1% | 43.4% | 118.8% |
| Eastern Mediterranean Region |  |  | - |  |  |  | 1 | 19 | 9 | 47.4% | 24.9% | 69.8% |
| Western Pacific Region | 1 | 2 | 35.00 | 21.14 | 48.86 |  | - |  |  |  |  |  |
| Other | 1 | 47 | 6.47 | -8.96 | 21.89 |  | 2 | 51 | 28 | 54.9% | 41.3% | 68.6% |
| **Income level** |  |  |  |  |  |  |  |  |  |  |  |  |
| High income | 11 | 358 | 32.11 | 26.12 | 38.11 |  | 10 | 312 | 161 | 52.0% | 33.4% | 70.6% |
| Lower middle income | 9 | 144 | 21.84 | 11.60 | 32.08 |  | 14 | 531 | 341 | 61.2% | 52.3% | 70.1% |
| Low income | 15 | 2964 | 14.76 | 13.44 | 16.09 |  | 17 | 4070 | 2342 | 57.4% | 53.9% | 61.0% |
| Others | 1 | 47 | 6.47 | -8.96 | 21.89 |  | 2 | 51 | 28 | 54.9% | 41.3% | 68.6% |
| **Type of cases** |  |  |  |  |  |  |  |  |  |  |  |  |
| All cases | 11 | 403 | 19.01 | 12.05 | 25.97 |  | 18 | 643 | 355 | 54.4% | 48.1% | 60.6% |
| Confirmed cases | 23 | 3089 | 22.33 | 17.28 | 27.37 |  | 19 | 4147 | 2431 | 62.6% | 56.8% | 68.4% |
| Suspected cases | 2 | 21 | 11.86 | -2.25 | 25.97 |  | 5 | 82 | 35 | 49.9% | 29.5% | 70.4% |
| Possible cases |  |  | - |  |  |  | 1 | 92 | 51 | 55.4% | 45.3% | 65.6% |
| **Sample size** |  |  |  |  |  |  |  |  |  |  |  |  |
| <5 | 7 | 19 | 22.64 | 15.04 | 30.25 |  | 3 | 10 | 5 | 50.0% | 20.1% | 79.9% |
| 5~99 | 24 | 721 | 22.11 | 16.62 | 27.61 |  | 31 | 892 | 490 | 56.6% | 48.7% | 64.5% |
| 100~500 | 3 | 956 | 12.90 | 2.79 | 23.02 |  | 6 | 1463 | 857 | 60.8% | 53.5% | 68.0% |
| >500 | 2 | 1817 | 12.47 | -4.14 | 29.07 |  | 3 | 2599 | 1520 | 59.0% | 53.7% | 64.3% |
| **Quality scores** |  |  |  |  |  |  |  |  |  |  |  |  |
| ≤5 | 3 | 45 | 24.30 | 5.79 | 42.82 |  | 4 | 210 | 139 | 62.1% | 45.6% | 78.7% |
| >5 | 33 | 3468 | 19.95 | 16.40 | 23.50 |  | 39 | 4754 | 2733 | 57.5% | 53.2% | 61.8% |

**Table S12: Estimates of duration of symptoms (days) for monkeypox cases (Global. 2022)**

|  |  | No. studies | Sample size | Effect | Lower limit | Higher limit |
| --- | --- | --- | --- | --- | --- | --- |
| **All** |  | 4 | 353 | 11.41 | 7.03 | 15.78 |
| **Study design** | |  |  |  |  |  |
| case report | | 1 | 3 | 17.67 | 3.76 | 31.57 |
| cross-sectional study | | 3 | 350 | 10.84 | 6.10 | 15.57 |
| **WHO region** | |  |  |  |  |  |
| African Region | | 1 | 282 | 16.38 | 6.05 | 26.72 |
| Region of the Americas | | 2 | 68 | 9.35 | 4.97 | 13.73 |
| European Region | | 1 | 3 | 17.67 | 3.76 | 31.57 |
| **Income level** | |  |  |  |  |  |
| High income | | 3 | 71 | 10.37 | 5.73 | 15.00 |
| Low income | | 1 | 282 | 16.38 | 6.05 | 26.72 |
| **Type of cases** | |  |  |  |  |  |
| all cases | | 1 | 282 | 16.38 | 6.05 | 26.72 |
| confirmed cases | | 3 | 71 | 10.37 | 5.73 | 15.00 |
| **Sample size** | |  |  |  |  |  |
| <5 | | 1 | 3 | 17.67 | 3.76 | 31.57 |
| 5~99 | | 2 | 68 | 9.35 | 4.97 | 13.73 |
| 100~500 | | 1 | 282 | 16.38 | 6.05 | 26.72 |
| **Type of symptoms** | | |  |  |  |  |
| all | | 2 | 285 | 16.84 | 8.55 | 25.13 |
| rash |  | 1 | 34 | 12.77 | 4.81 | 20.74 |
| fever | | 1 | 34 | 7.89 | 2.74 | 13.04 |

**Table S13: Estimates of comorbidity rate for monkeypox cases (Global. 2022)**

|  | No.Studies | Sample size | No.Cases with comorbidity | Effect | Lower limit | Higher limit |
| --- | --- | --- | --- | --- | --- | --- |
| **All** | 18 | 1918 | 355 | 15.5% | 9.7% | 21.4% |
| **Study design** |  |  |  |  |  |  |
| case report | 5 | 40 | 8 | 16.4% | 3.6% | 29.1% |
| cross-sectional study | 11 | 1740 | 332 | 16.8% | 9.6% | 24.1% |
| others | 2 | 138 | 15 | 8.2% | -3.8% | 20.1% |
| **WHO region** |  |  |  |  |  |  |
| Region of the Americas | 3 | 34 | 5 | 11.8% | 1.2% | 22.4% |
| African Region | 12 | 1782 | 333 | 15.3% | 8.5% | 22.0% |
| European Region | 3 | 102 | 17 | 24.7% | 0.7% | 48.7% |
| **Income level** |  |  |  |  |  |  |
| Lower middle income | 6 | 314 | 23 | 6.8% | 1.5% | 12.0% |
| Low income | 6 | 1468 | 310 | 20.4% | 10.6% | 30.3% |
| High income | 6 | 136 | 22 | 14.5% | 8.7% | 20.3% |
| **Proportion of MSM** |  |  |  |  |  |  |
| 100% | 2 | 6 | 3 | 50.0% | 10.0% | 90.0% |
| 95% | 2 | 113 | 17 | 15.0% | 8.4% | 21.6% |
| **Average age** |  |  |  |  |  |  |
| 18y~ | 12 | 450 | 45 | 9.8% | 4.8% | 14.8% |
| 0y~ | 5 | 1443 | 298 | 16.8% | 6.7% | 26.8% |
| **Percentage of men** |  |  |  |  |  |  |
| 75%~100% | 6 | 176 | 35 | 21.1% | 11.4% | 30.7% |
| 50%~ | 7 | 1112 | 169 | 8.9% | 1.5% | 16.4% |
| 25%~ | 2 | 54 | 2 | 5.9% | -14.5% | 26.3% |
| **Comorbidity type** |  |  |  |  |  |  |
| HIV | 5 | 165 | 31 | 20.2% | 9.8% | 30.6% |
| other immunodeficiency diseases | 4 | 266 | 11 | 3.3% | 1.2% | 5.4% |
| others | 9 | 1487 | 313 | 19.5% | 10.6% | 28.3% |
| **Quality scores** |  |  |  |  |  |  |
| >5 | 15 | 1668 | 332 | 15.9% | 9.2% | 22.6% |
| ≤5 | 3 | 250 | 23 | 15.3% | 1.6% | 29.1% |
| **Sample size** |  |  |  |  |  |  |
| <5 | 3 | 9 | 4 | 44.0% | 12.0% | 76.0% |
| 5~99 | 12 | 428 | 60 | 13.1% | 7.3% | 19.0% |
| 100~499 | 1 | 165 | 6 | 3.6% | 0.8% | 6.5% |
| ≥500 | 2 | 1316 | 285 | 22.1% | 16.4% | 27.7% |

**Table S14: Estimates of the case fatality rate for monkeypox cases (Global. 2022)**

|  | No.Studies | No.Valid cases | No.Death | Effect | Lower limit | Higher limit |
| --- | --- | --- | --- | --- | --- | --- |
| **All** | 46 | 63667 | 1533 | 3.8% | 3.2% | 4.3% |
| **Study design** |  |  |  |  |  |  |
| case report | 18 | 3733 | 184 | 4.7% | 4.0% | 5.3% |
| cross-sectional study | 12 | 20731 | 402 | 6.3% | 3.9% | 8.7% |
| others | 16 | 39203 | 947 | 3.0% | 2.2% | 3.7% |
| **Type of cases** |  |  |  |  |  |  |
| Confirmed | 13 | 1162 | 75 | 5.3% | 3.6% | 7.1% |
| All | 33 | 62505 | 1458 | 3.5% | 2.9% | 4.1% |
| **Income level** |  |  |  |  |  |  |
| Lower middle income | 17 | 1282 | 70 | 4.8% | 3.1% | 6.5% |
| Low income | 26 | 60798 | 1381 | 3.4% | 2.8% | 4.0% |
| Upper middle income | 1 | 4 | 2 | 50.0% | 1.0% | 99.0% |
| **Average age** |  |  |  |  |  |  |
| 18y~ | 12 | 1444 | 72 | 4.1% | 2.6% | 5.7% |
| 0y~ | 9 | 5510 | 237 | 6.6% | 3.9% | 9.2% |
| **Percentage of men** |  |  |  |  |  |  |
| 75%~100% | 2 | 63 | 10 | 15.0% | 6.2% | 23.8% |
| 50%~ | 14 | 1777 | 116 | 5.6% | 3.7% | 7.6% |
| 25%~ | 1 | 10 | 1 | 10.0% | -8.6% | 28.6% |
| **Sample size** |  |  |  |  |  |  |
| ≥500 | 8 | 60505 | 1333 | 2.9% | 2.3% | 3.6% |
| <5 | 1 | 4 | 2 | 50.0% | 1.0% | 99.0% |
| 5~99 | 30 | 1376 | 103 | 6.0% | 4.5% | 7.5% |
| 100~499 | 7 | 1782 | 93 | 4.7% | 2.4% | 7.0% |
| **Quality score** |  |  |  |  |  |  |
| >5 | 19 | 1920 | 118 | 5.5% | 3.6% | 7.5% |
| ≤5 | 16 | 38910 | 927 | 3.5% | 2.6% | 4.4% |

**Table S15: Estimates of secondary attack rate for monkeypox cases (Global. 2022)**

|  | No.  Studies | No.  Contacts | No.  Secondary cases | Effect | Lower limit | Higher limit |
| --- | --- | --- | --- | --- | --- | --- |
| **All** | 8 | 11209 | 538 | 4.2% | 2.2% | 6.2% |
| **Study design** |  |  |  |  |  |  |
| case report | 3 | 493 | 15 | 3.9% | -1.4% | 9.2% |
| cross-sectional study | 3 | 6361 | 178 | 2.8% | 2.4% | 3.2% |
| others | 2 | 4355 | 345 | 7.9% | 7.1% | 8.7% |
| **WHO region** |  |  |  |  |  |  |
| African Region | 6 | 10883 | 535 | 4.8% | 2.8% | 6.9% |
| European Region | 2 | 326 | 3 | 1.5% | -2.6% | 5.5% |
| **Income level** |  |  |  |  |  |  |
| Lower middle income | 1 | 167 | 12 | 7.2% | 3.3% | 11.1% |
| Low income | 5 | 10716 | 523 | 4.5% | 2.4% | 6.7% |
| High income | 2 | 326 | 3 | 1.5% | -2.6% | 5.5% |
| **Quality score** |  |  |  |  |  |  |
| >5 | 5 | 10461 | 505 | 4.2% | 1.9% | 6.5% |
| ≤5 | 2 | 455 | 13 | 3.5% | -3.2% | 10.2% |
| **Sample size** |  |  |  |  |  |  |
| 5~99 | 1 | 38 | 2 | 5.3% | -1.8% | 12.4% |
| 100~499 | 3 | 748 | 33 | 4.6% | -0.7% | 9.9% |
| ≥500 | 4 | 10423 | 503 | 4.1% | 1.7% | 6.5% |

**Table S16: Estimates of incubation period (days) for monkeypox cases (Global. 2022)**

|  | No. studies | Sample size | Effect | Lower limit | Higher limit |
| --- | --- | --- | --- | --- | --- |
| **All** | 5 | 96 | 9.63 | 5.87 | 13.40 |
| **Study design** |  |  |  |  |  |
| case report | 1 | 35 | 14.85 | -30.61 | 60.30 |
| cross-sectional study | 4 | 61 | 9.60 | 5.82 | 13.38 |
| **WHO region** |  |  |  |  |  |
| African Region | 4 | 16 | 9.17 | 4.18 | 14.15 |
| Region of the Americas | 1 | 80 | 10.26 | 4.51 | 16.00 |
| **Income level** |  |  |  |  |  |
| High income | 1 | 80 | 10.26 | 4.51 | 16.00 |
| Low income | 4 | 16 | 9.17 | 4.18 | 14.15 |
| **Type of cases** |  |  |  |  |  |
| all cases | 1 | 67 | 9.05 | 5.03 | 13.07 |
| confirmed cases | 4 | 29 | 13.79 | 3.07 | 24.50 |
| **Quality scores** |  |  |  |  |  |
| ≤5 | 1 | 16 | 9.17 | 4.18 | 14.15 |
| >5 | 4 | 80 | 10.26 | 4.51 | 16.00 |

**Table S17: Estimates of proportion of animal contact history for monkeypox cases (Global. 2022)**

|  | No.Studies | Sample size | No.Animal contact cases | Effect | Lower limit | Higher limit |
| --- | --- | --- | --- | --- | --- | --- |
| **All** | 25 | 5345 | 2456 | 37.8% | 22.6% | 53.1% |
| **Study design** |  |  |  |  |  |  |
| case report | 7 | 106 | 41 | 25.4% | 6.9% | 43.9% |
| cross-sectional study | 15 | 4759 | 2125 | 40.9% | 21.7% | 60.1% |
| others | 3 | 480 | 290 | 44.3% | 19.3% | 69.3% |
| **WHO region** |  |  |  |  |  |  |
| Region of the Americas | 4 | 223 | 116 | 52.1% | 45.5% | 58.6% |
| African Region | 20 | 5120 | 2339 | 34.4% | 17.4% | 51.3% |
| European Region | 1 | 2 | 1 | 50.0% | -19.3% | 119.3% |
| **Income level** |  |  |  |  |  |  |
| Lower middle income | 3 | 134 | 14 | 13.3% | -0.9% | 27.6% |
| Low income | 17 | 4986 | 2325 | 36.7% | 18.0% | 55.3% |
| High income | 5 | 225 | 117 | 52.0% | 45.5% | 58.5% |
| **Average age** |  |  |  |  |  |  |
| 18y~ | 8 | 1035 | 113 | 26.1% | 13.0% | 39.1% |
| 0y~ | 14 | 3871 | 2063 | 39.5% | 13.9% | 65.2% |
| **Percentage of men** |  |  |  |  |  |  |
| 75%~100% | 5 | 714 | 15 | 25.0% | -0.9% | 50.8% |
| 50%~ | 13 | 3945 | 2020 | 36.7% | 10.3% | 63.1% |
| 25%~ | 5 | 218 | 101 | 37.3% | 20.4% | 54.2% |
| 0%~ | 1 | 6 | 1 | 16.7% | -13.2% | 46.5% |
| **Types of animal** |  |  |  |  |  |  |
| non-human primates | 2 | 1155 | 839 | 69.4% | 54.1% | 84.7% |
| rodents | 2 | 1155 | 837 | 58.0% | -6.6% | 122.7% |
| consume bush meat | 2 | 8 | 2 | 21.9% | -5.5% | 49.3% |
| others | 3 | 405 | 90 | 41.7% | 7.4% | 75.9% |
| **Quality score** |  |  |  |  |  |  |
| >5 | 20 | 4140 | 2119 | 36.9% | 16.3% | 57.5% |
| ≤5 | 4 | 815 | 85 | 35.5% | -11.7% | 82.6% |
| **Sample size** |  |  |  |  |  |  |
| 5~99 | 12 | 397 | 197 | 34.7% | 16.8% | 52.5% |
| 100~499 | 6 | 1818 | 831 | 40.9% | 18.1% | 63.8% |
| ≥500 | 4 | 3121 | 1425 | 43.0% | 4.4% | 81.5% |
| <5 | 3 | 9 | 3 | 30.2% | 1.6% | 58.7% |

**Table S18: Estimates of proportion of travel history for monkeypox cases (Global. 2022)**

|  | No.Studies | Sample size | No.Travel-related cases | Effect | Lower limit | Higher limit |
| --- | --- | --- | --- | --- | --- | --- |
| **All** | 13 | 750 | 264 | 34.5% | 17.8% | 51.2% |
| **Study design** | |  |  |  |  |  |
| case report | 3 | 91 | 3 | 4.3% | -6.7% | 15.3% |
| cross-sectional study | 2 | 13 | 8 | 61.8% | 35.5% | 88.1% |
| others | 8 | 646 | 253 | 36.8% | 22.2% | 51.4% |
| **WHO region** | |  |  |  |  |  |
| European Region | 8 | 309 | 107 | 36.3% | 10.5% | 62.0% |
| Region of the Americas | 2 | 10 | 2 | 17.9% | -5.4% | 41.2% |
| African Region | 3 | 431 | 152 | 36.3% | 17.2% | 55.3% |
| **Income level** | |  |  |  |  |  |
| Lower middle income | 1 | 6 | 4 | 66.7% | 28.9% | 104.4% |
| Low income | 2 | 425 | 148 | 32.0% | 18.6% | 45.5% |
| High income | 9 | 312 | 111 | 36.0% | 11.5% | 60.5% |
| Upper middle income | 1 | 7 | 1 | 14.3% | -11.6% | 40.2% |
| **Reported time** | |  |  |  |  |  |
| 2022 | 7 | 307 | 106 | 29.6% | 3.7% | 55.5% |
| before 2022 | 6 | 443 | 158 | 37.0% | 27.1% | 46.9% |
| **Traveling area** | |  |  |  |  |  |
| Nigeria | 5 | 25 | 11 | 41.6% | 18.3% | 65.0% |
| Europe and West Africa | 1 | 29 | 23 | 79.3% | 64.6% | 94.1% |
| Europe | 2 | 10 | 2 | 17.9% | -5.4% | 41.2% |
| Europe and Brazil | 1 | 27 | 4 | 14.8% | 1.4% | 28.2% |
| **Average age** | |  |  |  |  |  |
| 18y~ | 6 | 303 | 108 | 36.7% | 8.1% | 65.2% |
| 0y~ | 1 | 6 | 1 | 16.7% | -13.2% | 46.5% |
| **Percentage of men** | |  |  |  |  |  |
| 75%~100% | 6 | 303 | 108 | 36.7% | 8.1% | 65.2% |
| 50%~ | 2 | 13 | 5 | 35.4% | -4.1% | 75.0% |
| **Quality scores** | |  |  |  |  |  |
| >5 | 9 | 582 | 183 | 31.7% | 12.3% | 51.1% |
| ≤5 | 4 | 168 | 81 | 41.2% | 19.7% | 62.7% |
| **Sample size** | |  |  |  |  |  |
| 100~499 | 2 | 571 | 222 | 41.8% | 27.9% | 55.8% |
| 5~99 | 8 | 171 | 39 | 31.9% | 7.7% | 56.1% |
| <5 | 3 | 8 | 3 | 37.1% | 4.0% | 70.3% |

**Table S19: Estimates of proportion of men who make sex with men for monkeypox cases (Global. 2022)**

|  | No.  Studies | Sample size | No.MSM | Effect | Lower limit | Higher limit |
| --- | --- | --- | --- | --- | --- | --- |
| **All** | 9 | 510 | 392 | 79.8% | 65.5% | 94.2% |
| **Study design** | |  |  |  |  |  |
| case report | 4 | 125 | 104 | 87.2% | 76.7% | 97.7% |
| others | 5 | 385 | 288 | 75.0% | 49.2% | 100.8% |
| **WHO region** | |  |  |  |  |  |
| European Region | 8 | 493 | 376 | 77.5% | 61.2% | 93.8% |
| Region of the Americas | 1 | 17 | 16 | 94.1% | 82.9% | 105.3% |
| **Income level** | |  |  |  |  |  |
| High income | 9 | 510 | 392 | 79.8% | 65.5% | 94.2% |
| **Reported time** | |  |  |  |  |  |
| 2022 | 9 | 510 | 392 | 79.8% | 65.5% | 94.2% |
| **Quality score** | |  |  |  |  |  |
| >5 | 5 | 335 | 227 | 82.5% | 65.6% | 99.3% |
| ≤5 | 4 | 175 | 165 | 73.7% | 46.0% | 101.3% |
| **Sample size** | |  |  |  |  |  |
| 5~99 | 7 | 159 | 130 | 84.8% | 76.2% | 93.5% |
| 100~499 | 2 | 351 | 262 | 77.7% | 35.0% | 120.4% |

**
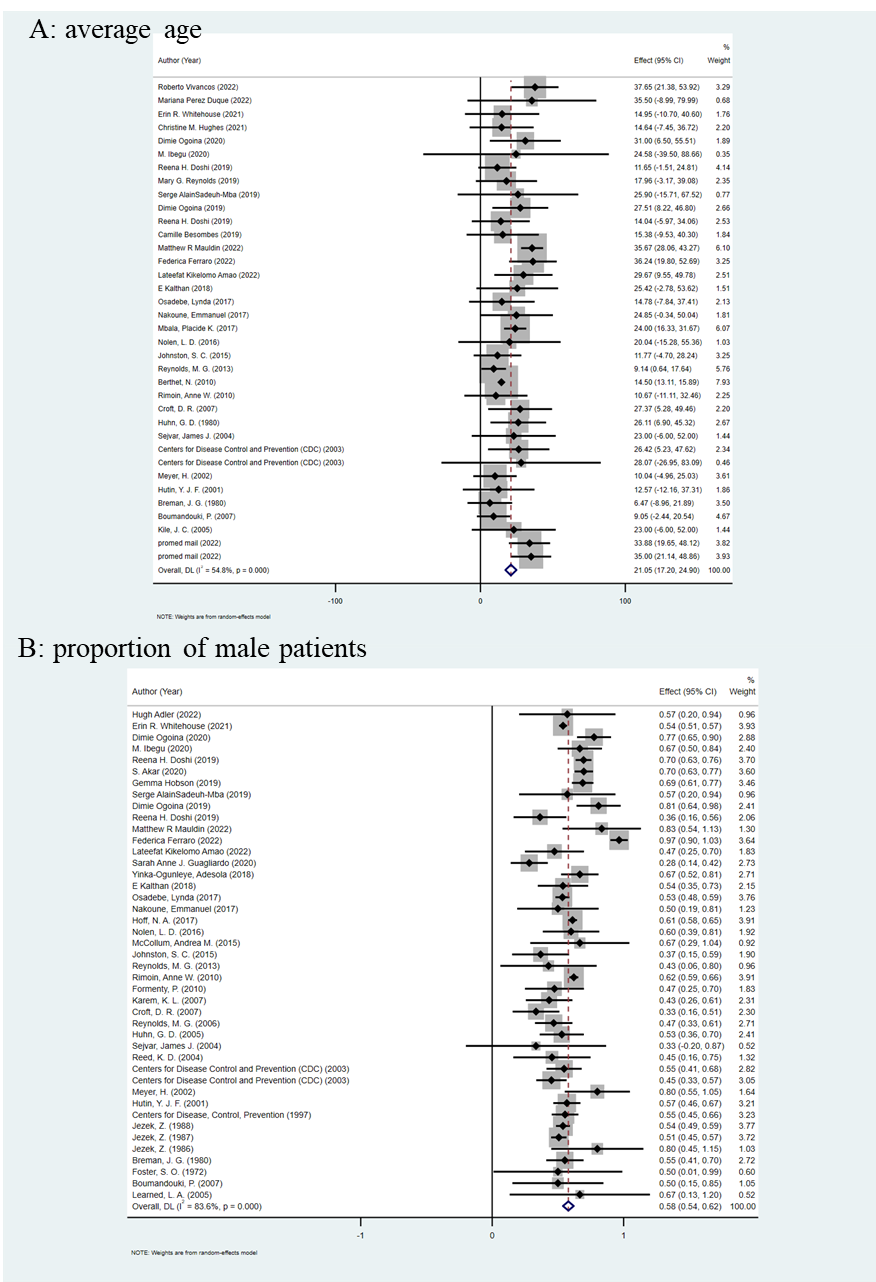
**

**Figure S1: Forest plots for the estimation of average age and proportion of male patients (Global. 2022)**

**
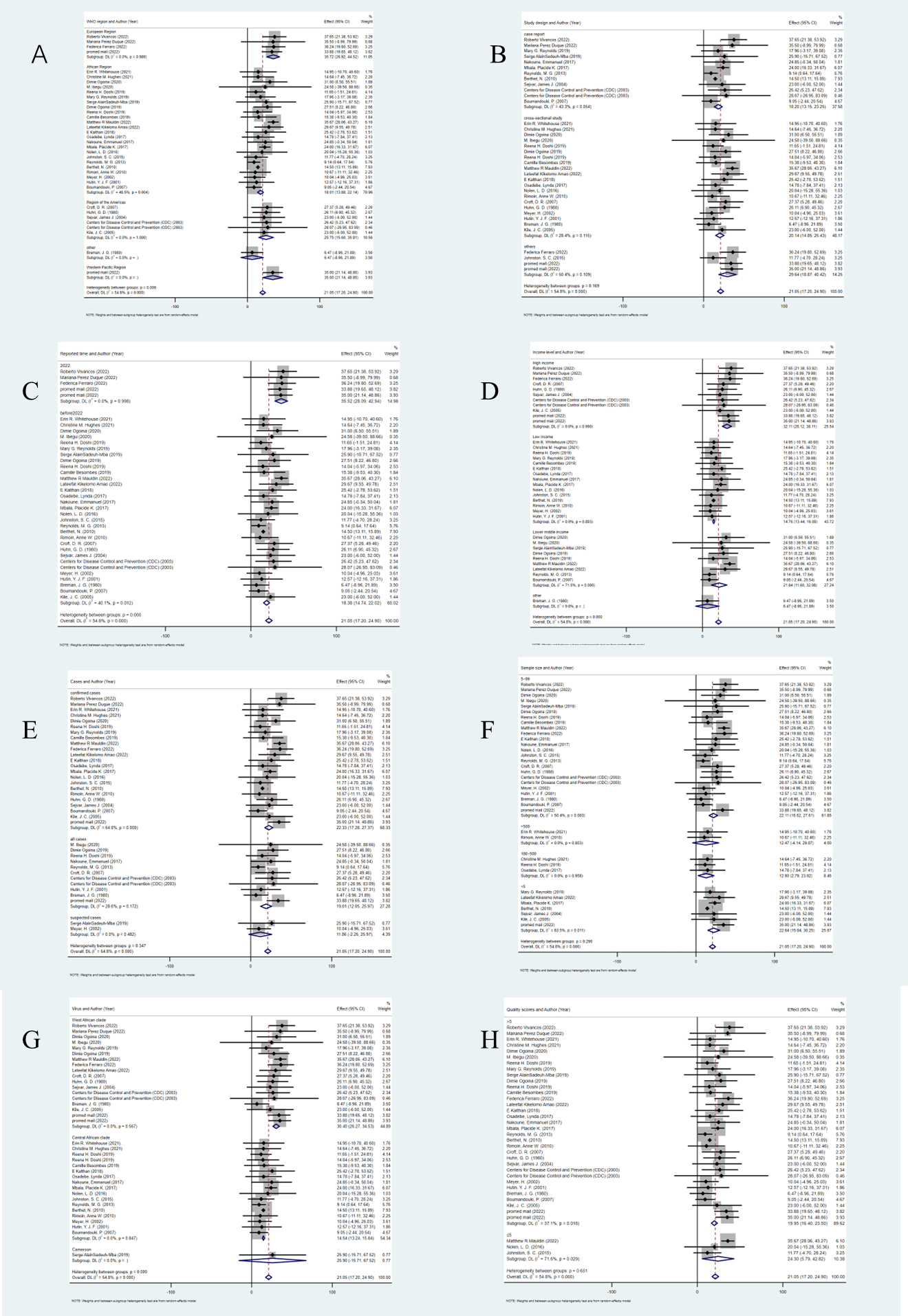
**

**Figure S2: Forest plots for the estimation of average age by subgroups (Global. 2022)**

**A: Study design; B: WHO region; C: Income level; D: Reported time; E: Type of cases; F: Sample size; G: Virus; H: Quality scores**

**
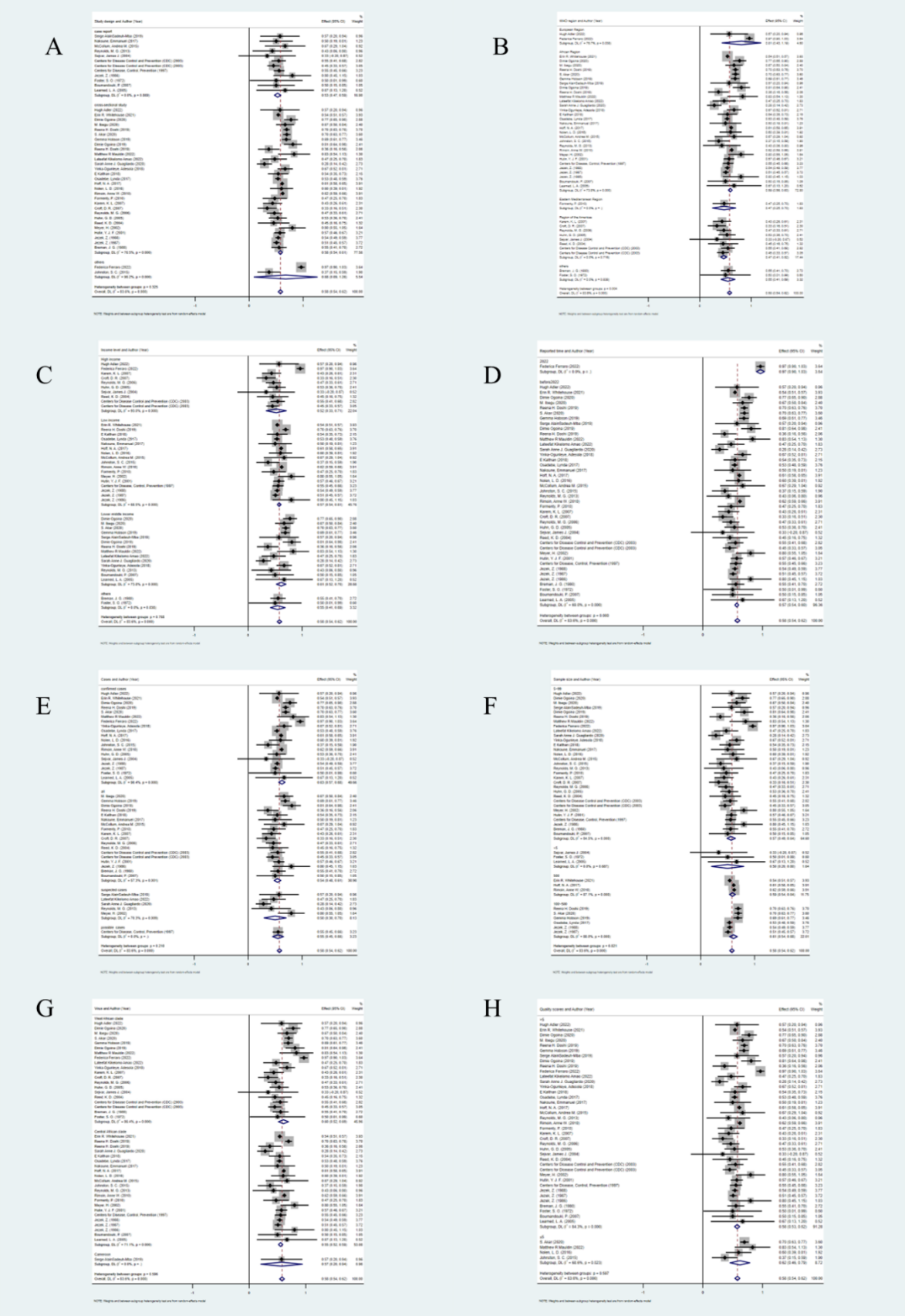
**

**Figure S3: Forest plots for proportion of male patients by subgroups (Global. 2022)**

**A: Study design; B: WHO region; C: Income level; D: Reported time; E: Type of cases; F: Sample size; G: Virus; H: Quality scores**

**
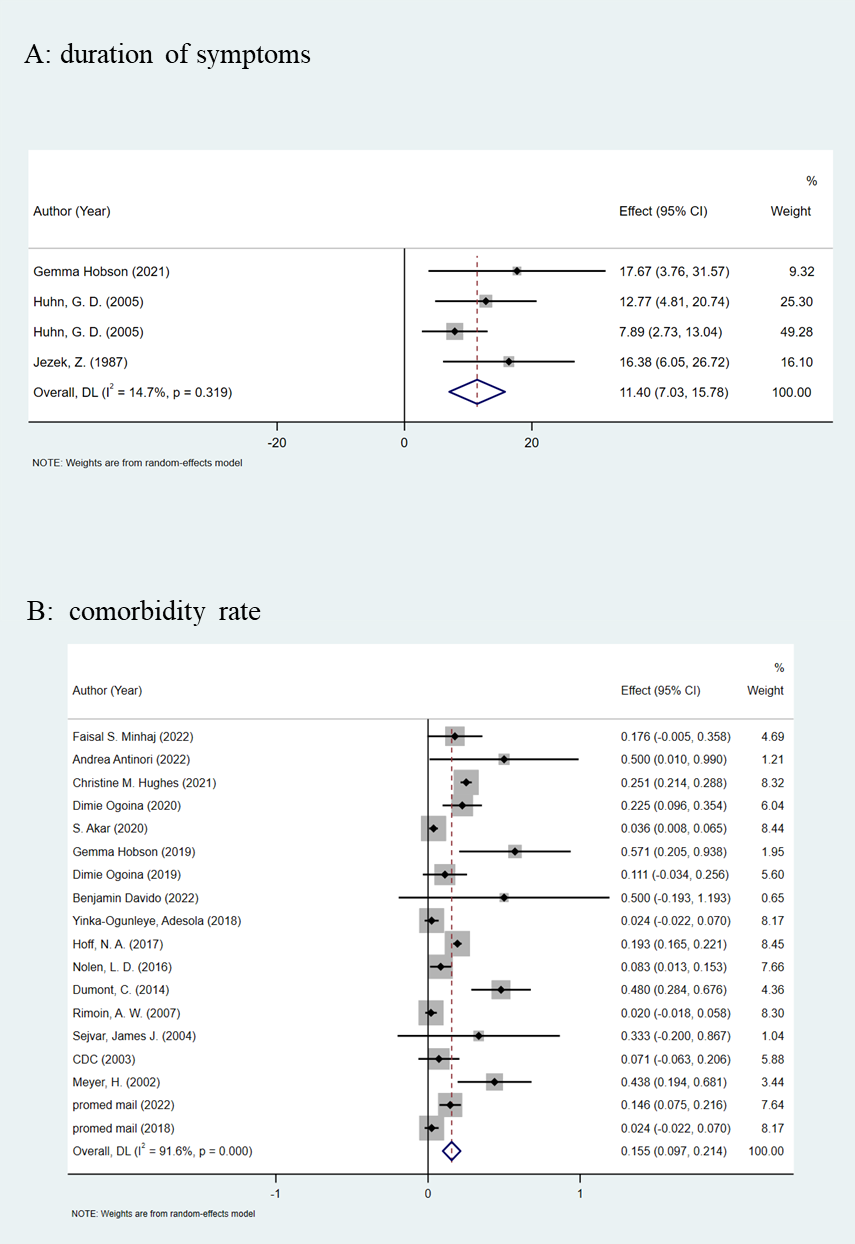
**

**Figure S4: Forest plots for the estimation of duration of symptoms (days) and comorbidity rate (Global. 2022)**

**
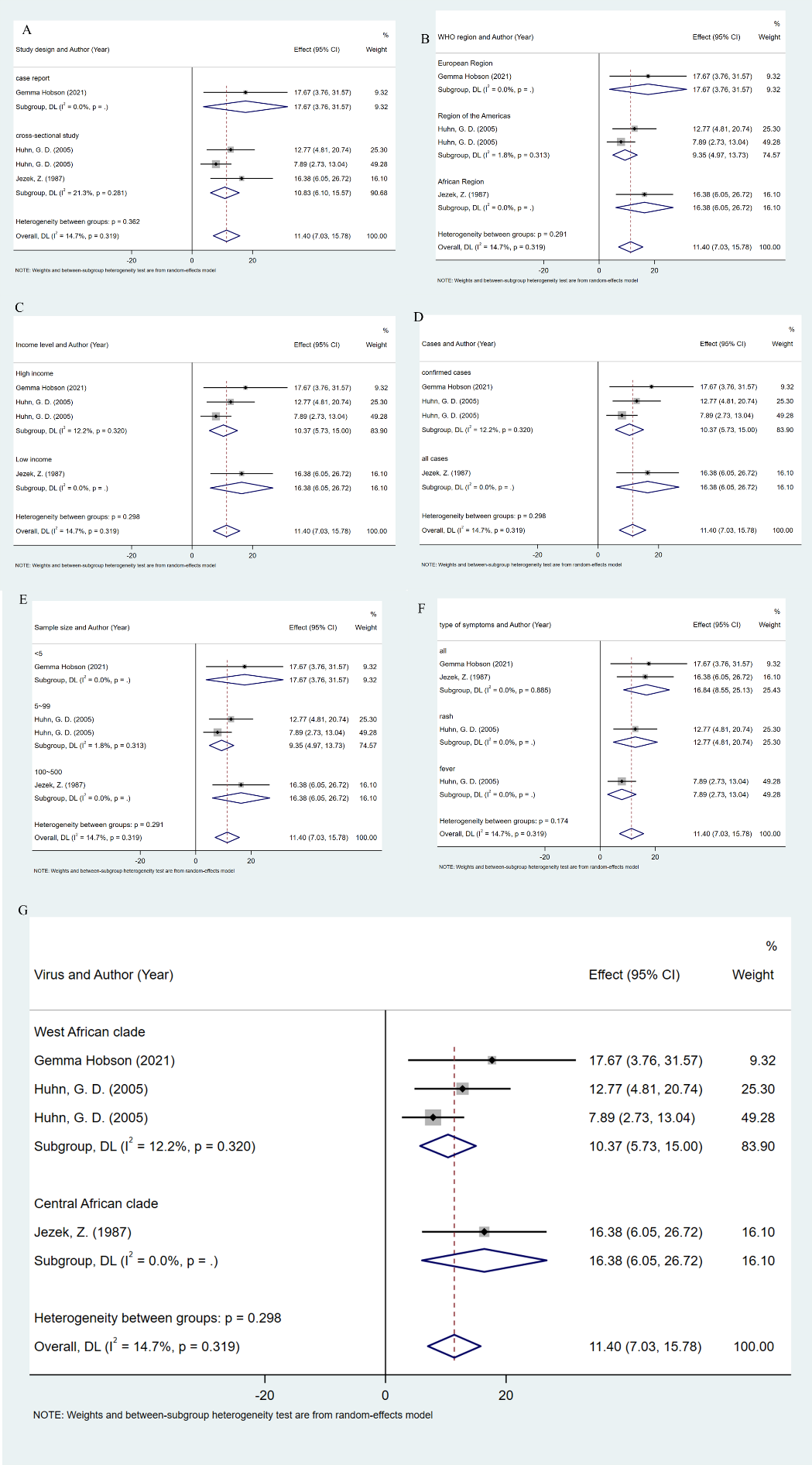
**

**Figure S5: Forest plots for the estimation of duration of symptoms (days) by subgroups (Global. 2022)**

**A: Study design; B: WHO region; C: Income level; D: Type of cases; E: Sample size; F: Type of symptoms; G:** **Virus**

**
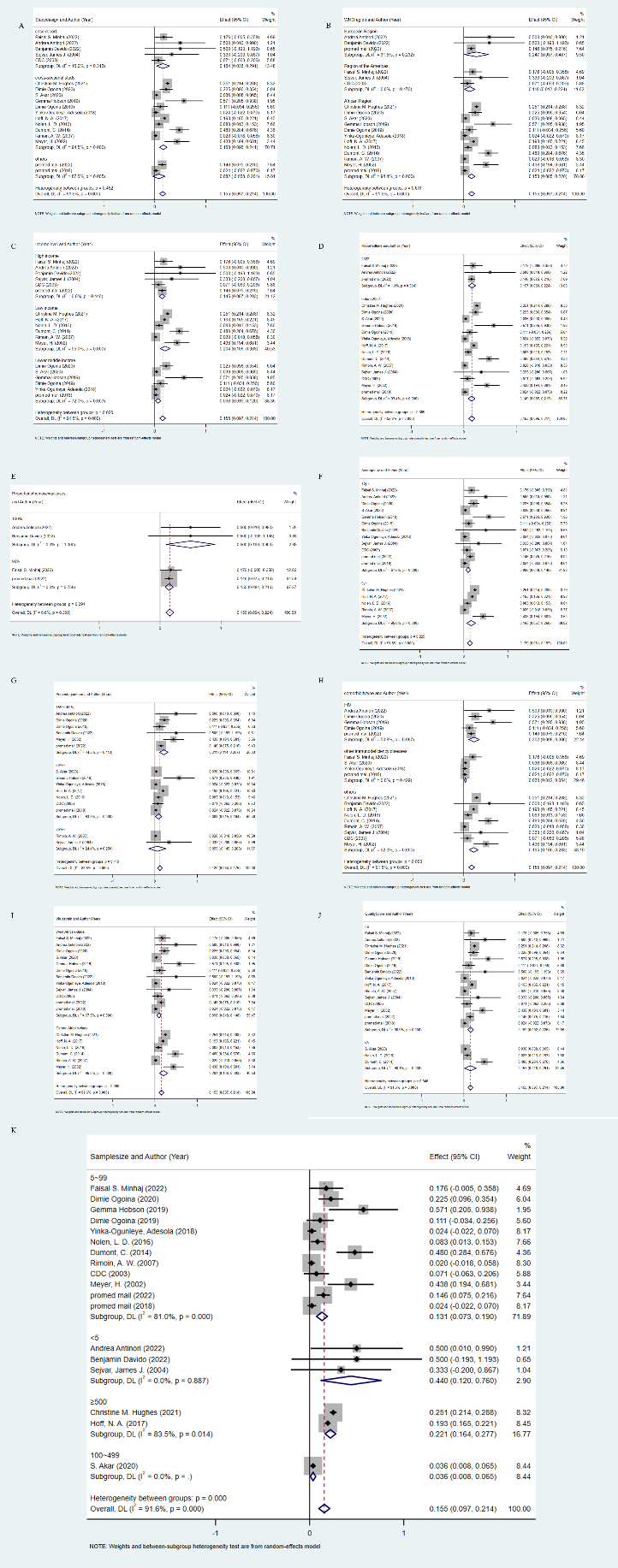
**

**Figure S6: Forest plots for the estimation of comorbidity rate by subgroups (Global. 2022)**

**A: Study design; B: WHO region; C: Income level; D: Reported time; E: Proportion of MSM; F: Average age; G:** **Percentage of men; H: Comorbidity type; I: Virus strain; J: Quality scores; K: Sample size.**

**
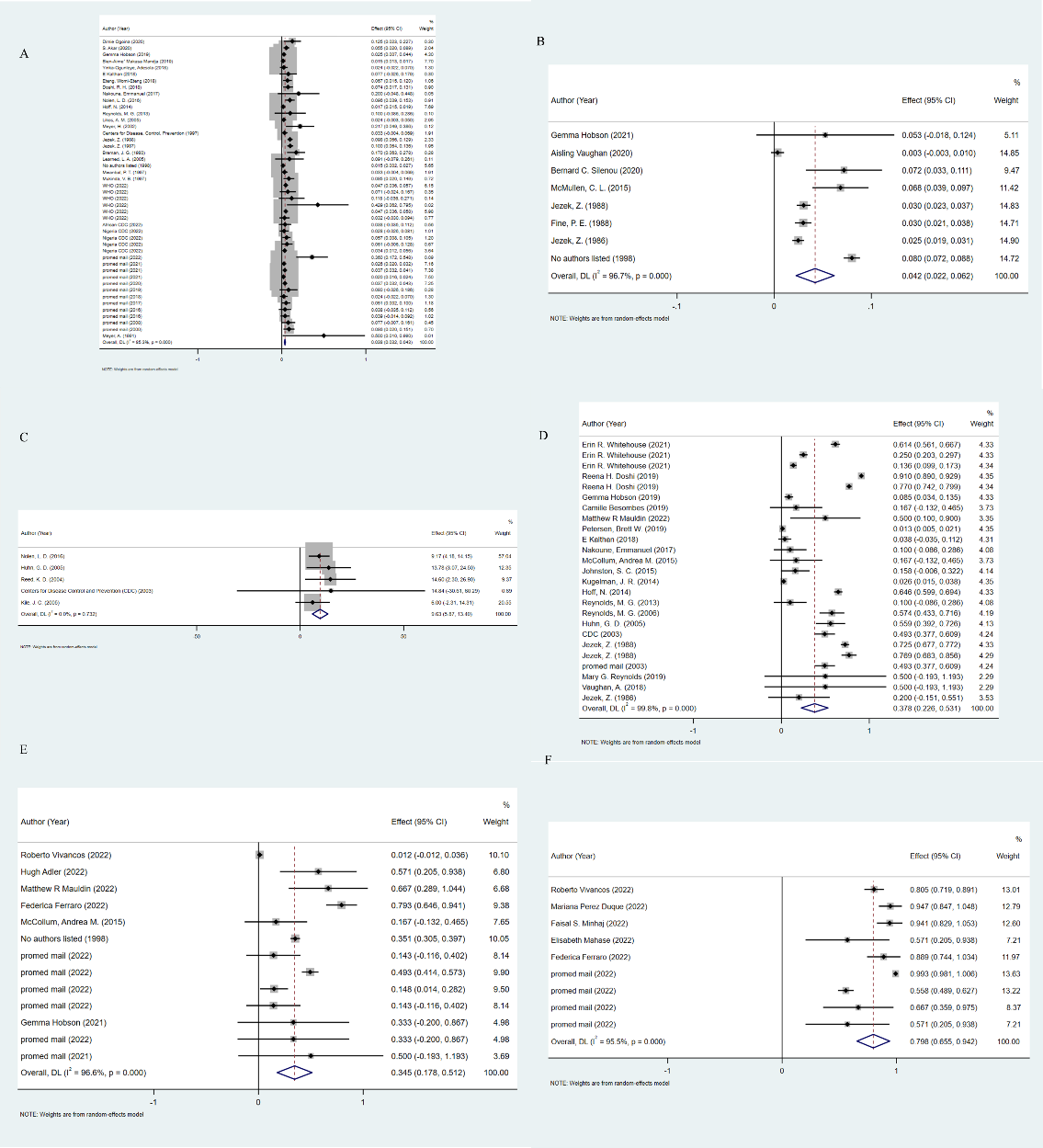
**

**Figure S7: Forest plots for the estimation of epidemiological characteristics for monkeypox cases (Global. 2022)**

**A: Case fatality rate; B: Secondary attack rate; C: Average incubation period (days) ; D: Proportion of animal contact history; E: Proportion of travel history; F: Proportion of homosexual.**

**
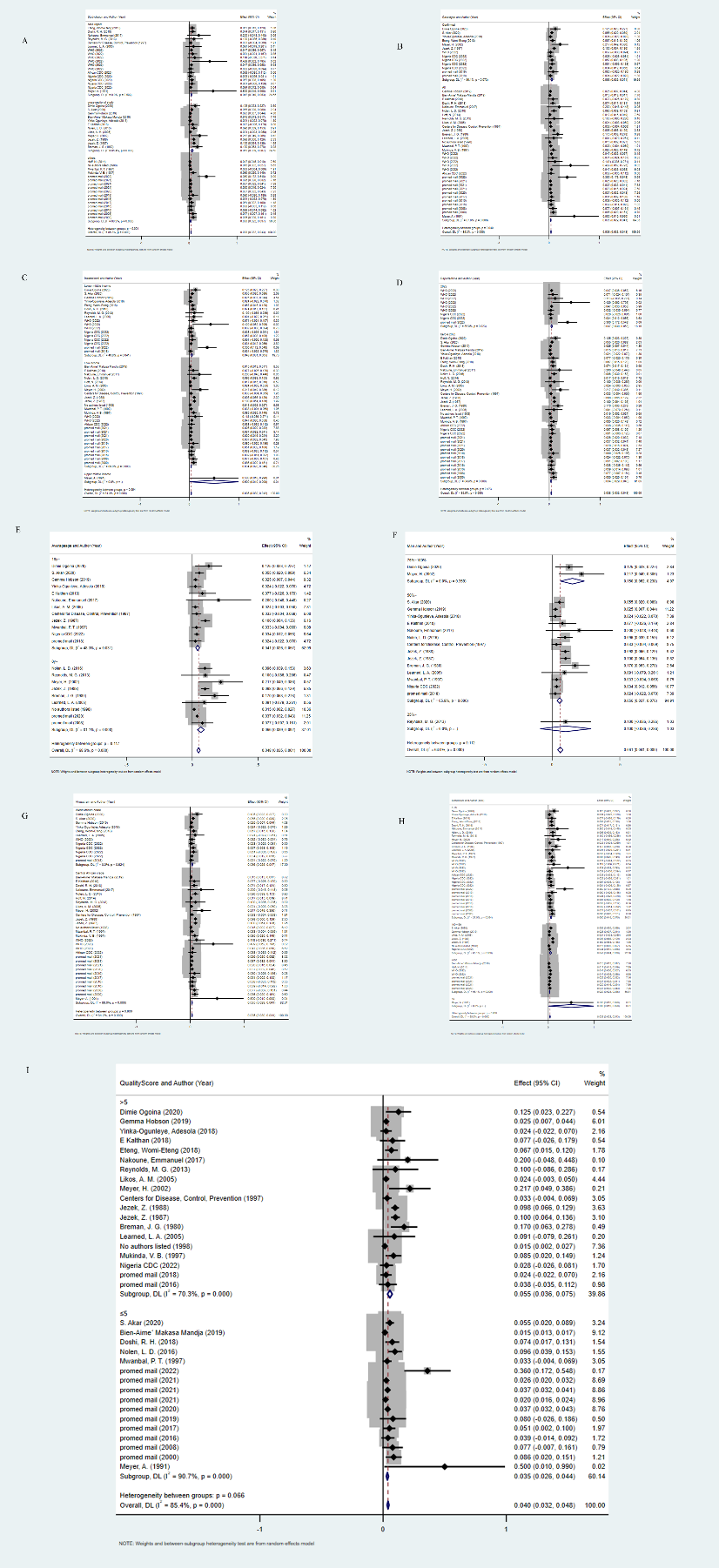
**

**Figure S8: Forest plots for the estimation of case fatality rates by subgroups (Global. 2022)**

**A: Study design; B: Type of cases; C: Income level; D: Reported time; E: Average age; F:** **Percentage of men; G: Virus strain; H: Sample size; I: Quality scores.**

**
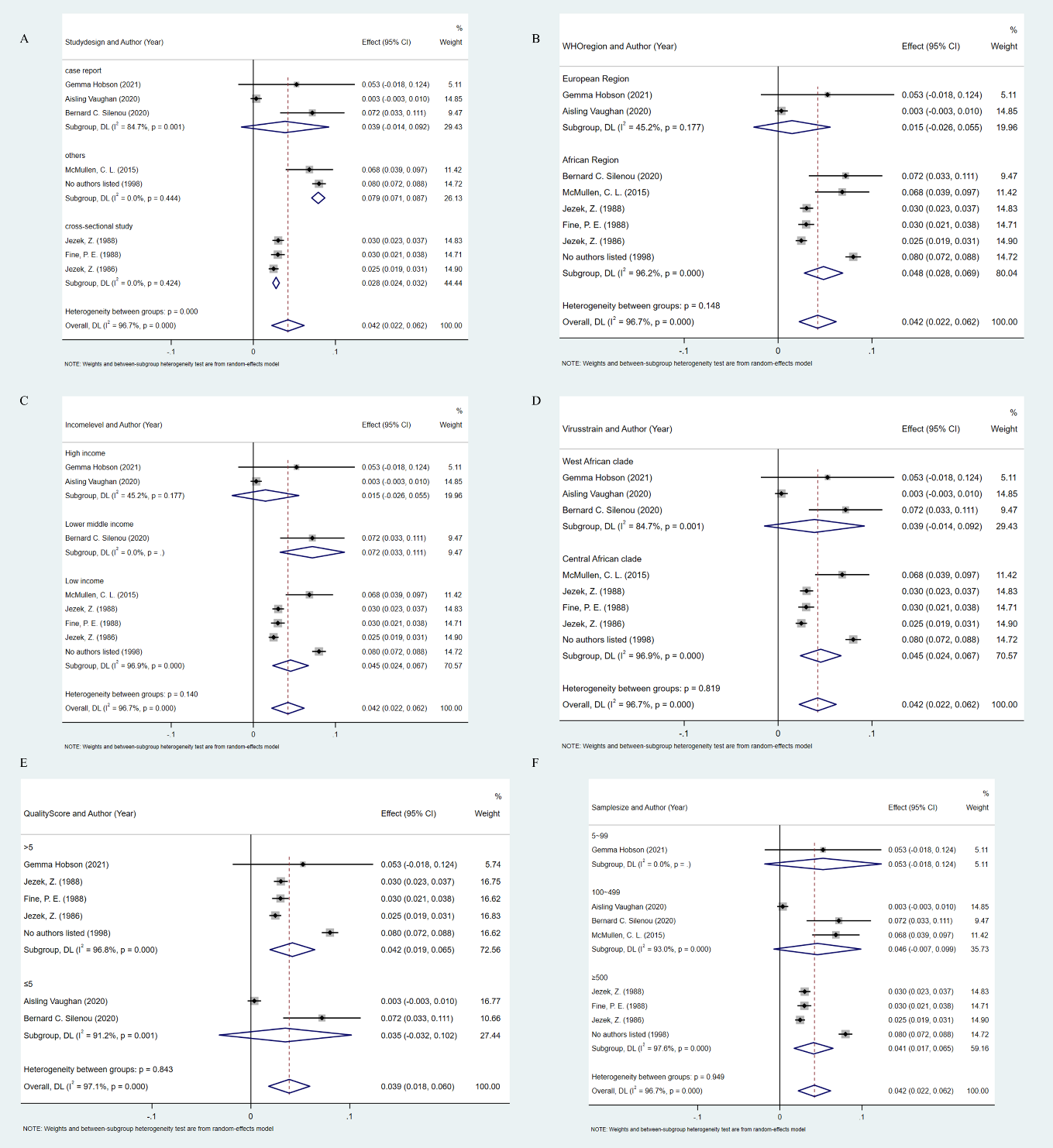
**

**Figure S9: Forest plots for the estimation of secondary attack rate by subgroups (Global. 2022)**

**A: Study design; B: WHO region; C: Income level; D: Virus strain; E: Quality scores; F: Sample size.**

**
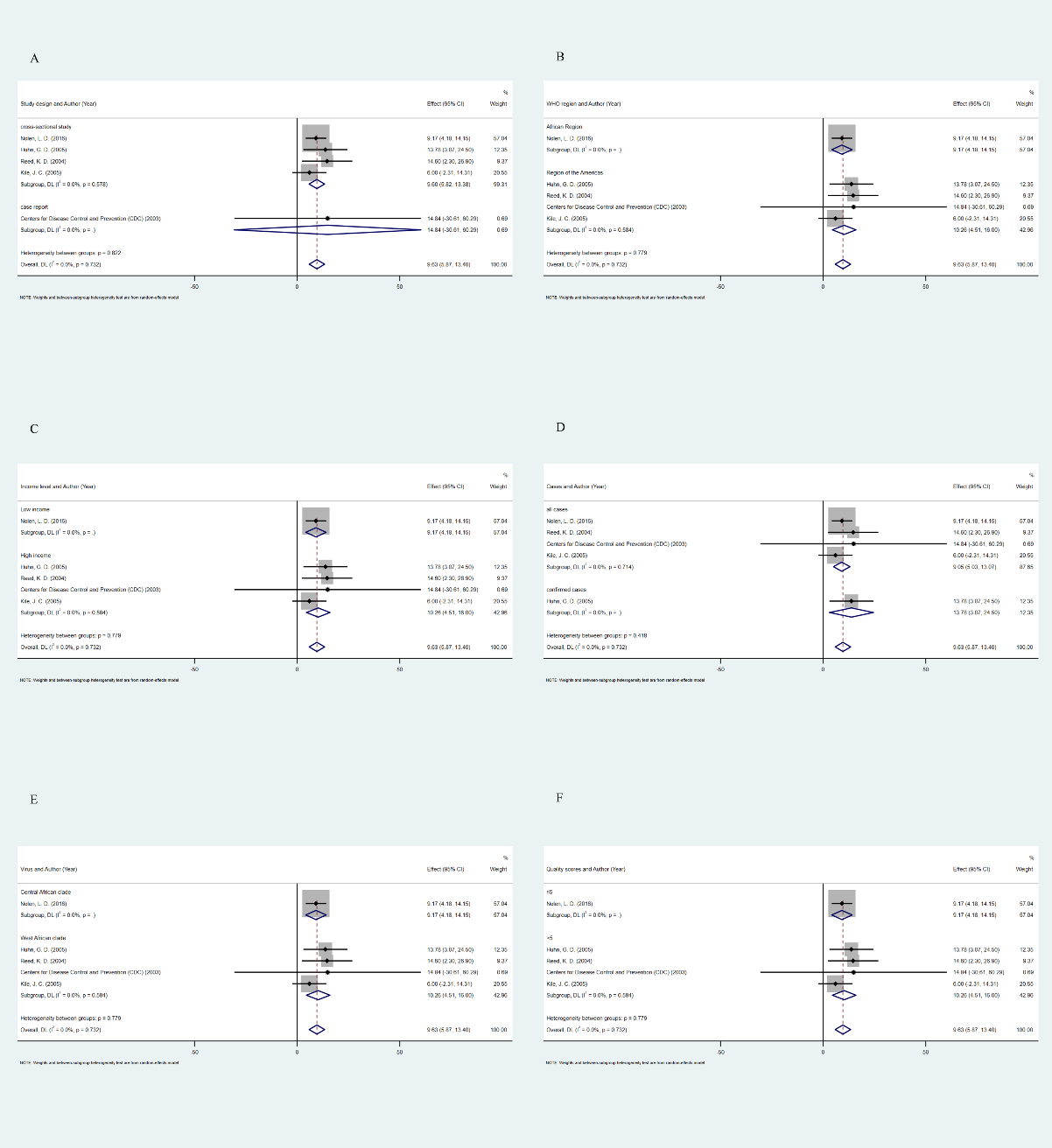
**

**Figure S10: Forest plots for the estimation of average incubation period by subgroups (Global. 2022)**

**A: Study design; B: WHO region; C: Income level; D: Type of cases; E: Virus; F: Quality scores.**

**
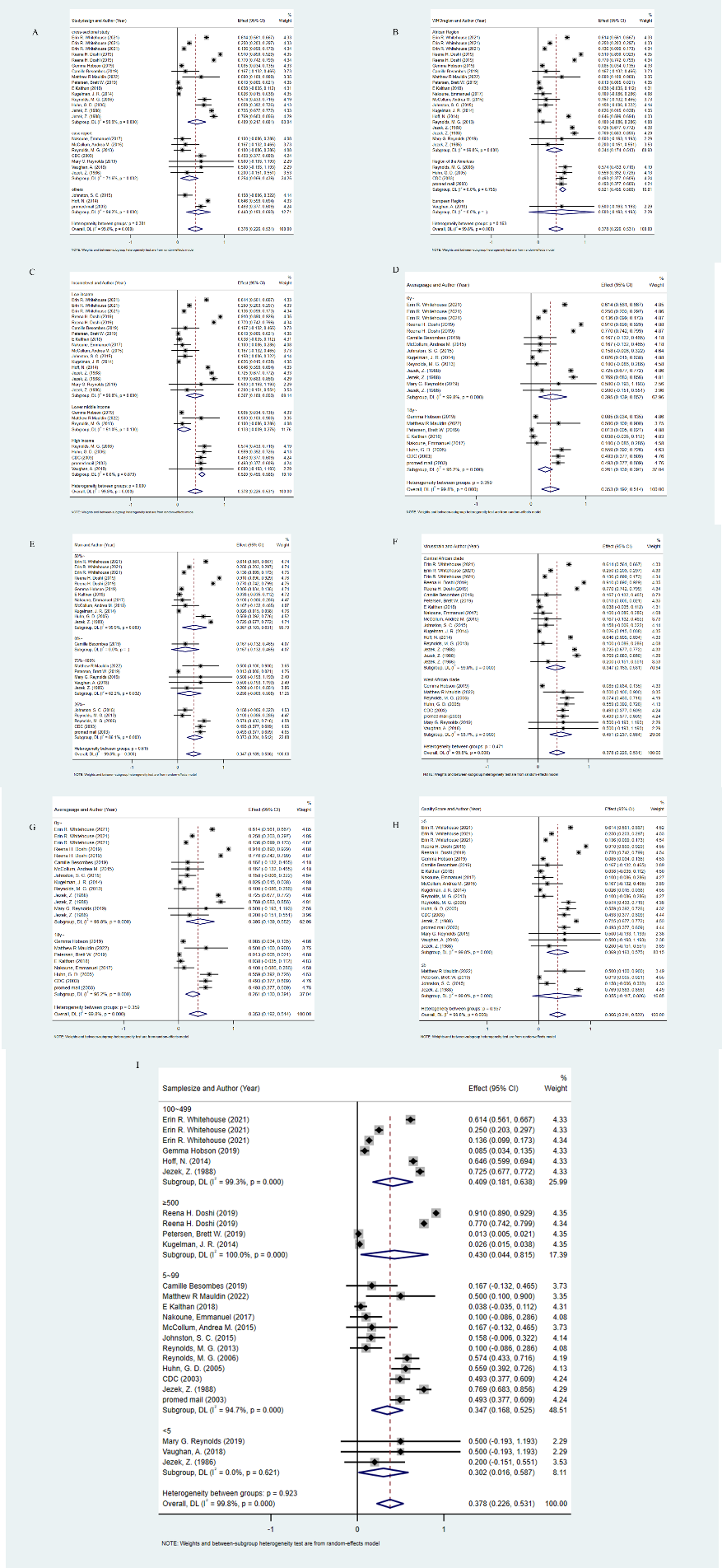
**

**Figure S11: Forest plots for the estimation of proportion of animal contact history by subgroups (Global. 2022)**

**A: Study design; B: WHO region; C: Income level; D: Average age; E: Percentage of men; F: Virus strain; G: Types of animal; H: Quality scores; I: Sample size.**

**
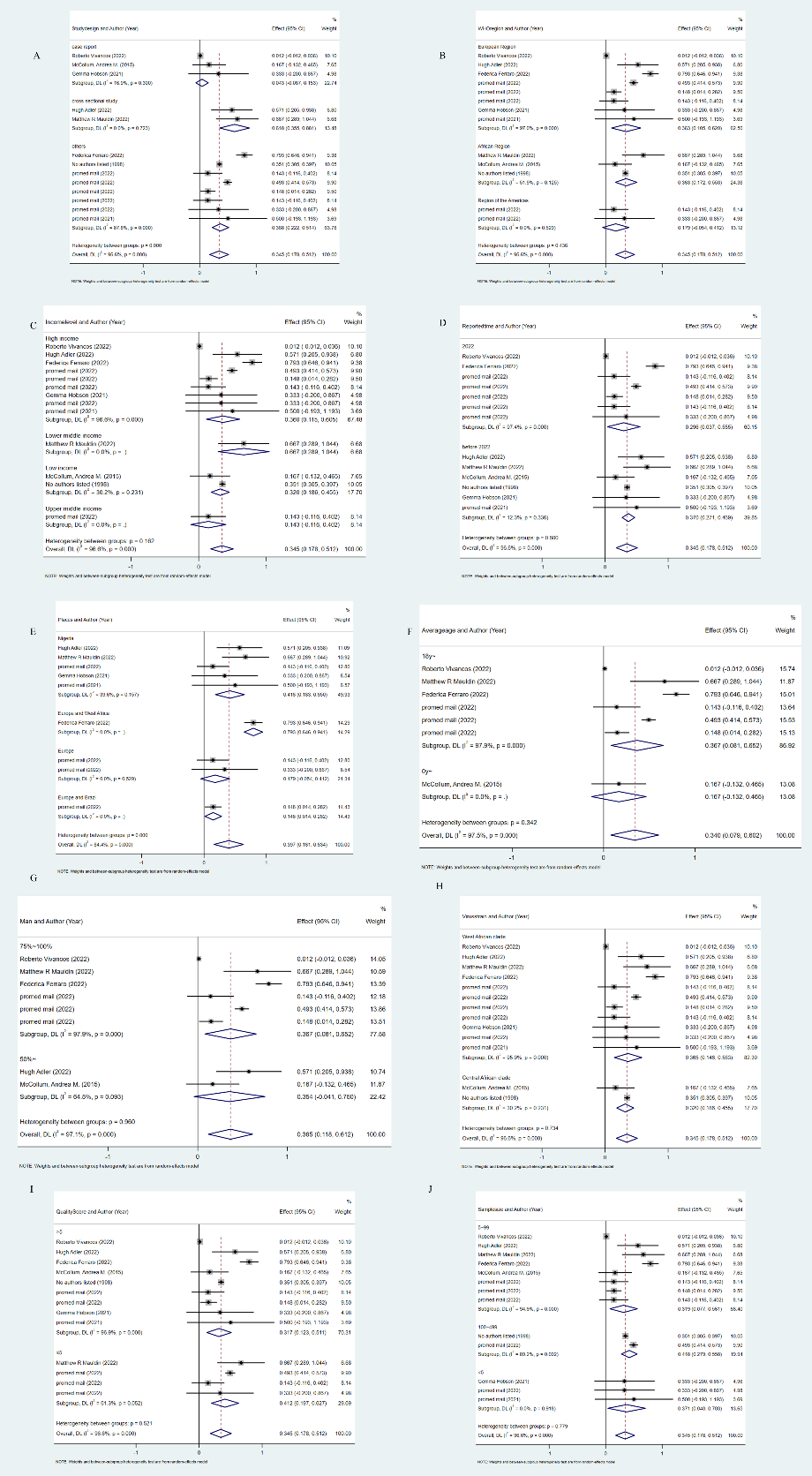
**

**Figure S12: Forest plots for the estimation of proportion of travel history by subgroups (Global. 2022)**

**A: Study design; B: WHO region; C: Income level; D: Reported time; E: Traveling area; F: Average age; G: Percentage of men; H: Virus strain; I: Quality scores; J: Sample size.**

Sensitivity analysis for estimation of average age and proportion of male patients:

1. Estimation of average age: after excluding 7 studies with sample size less than 5, the pooled average age of monkeypox cases was 20.52 years (95%CI: 15.78, 25.27), which was nearly with the original results. After excluding 3 studies with quality scores≤5, the pooled average age was 19.95 years (95%CI: 16.40, 23.50) which was slightly lower than the original results.
2. Estimation of the proportion of male patients: After excluding 14 studies with sample size less than 5, the proportion of monkeypox cases among the tested population was 58.1% (95%CI: 53.9%, 62.2%), which was slightly higher with the original results. After excluding 6 studies with quality scores≤5, the proportion of male patients was 57.5% (95%CI: 53.2%, 61.8%) which was slightly lower than the original results.
3. Estimation of the duration of symptoms (days): After excluding 1 study with sample size less than 5, the average duration of symptoms among monkeypox cases was 10.84 d (95%CI: 6.10, 15.57), which was slightly higher with the original results.
4. Estimation of the comorbidity rate: After excluding 3 studies with sample size less than 5, the pooled comorbidity rate of monkeypox cases among the tested population was 14.7% (95%CI: 8.8%, 20.6%), which was lower than the original results. After excluding 3 studies with quality scores≤5, the pooled comorbidity rate of monkeypox cases was 15.9% (95%CI: 9.2%, 22.6%) which was nearly with the original results.
5. Estimation of case fatality rate: After excluding 1 studies with sample size less than 5, the pooled case fatality rate was 3.8% (95%CI: 3.2%, 4.3%), which was as same as the original results. After excluding 16 studies with quality scores≤5, the pooled case fatality rate was 4.7% (95%CI: 3.5%, 5.9%) which was higher than the original results.
6. Estimation of secondary attack rate: After excluding 2 studies with quality scores≤5, the pooled secondary attack rate of monkeypox cases was 4.6% (95%CI: 2.5%, 6.7%) which was slightly higher than the original results.
7. Estimation of incubation period (days): After excluding 1 study with quality scores≤5, the incubation period was 10.26 d (95%CI: 4.51, 16.00) which was slightly lower than the original results.
8. Estimation of proportion of animal contact history: After excluding 2 studies with sample size less than 5, the pooled proportion of animal contact history was 37.3% (95%CI: 21.7%, 52.9%), which was slightly lower than the original results. After excluding 4 studies with quality scores≤5, the pooled proportion of animal contact history was 38.3% (95%CI: 18.5%, 58.1%) which was nearly with the original results.
9. Estimation of proportion of travel history: After excluding 3 studies with sample size less than 5, the pooled proportion of travel history was 34.0% (95%CI: 15.9%, 52.0%), which was nearly with the original results. After excluding 4 studies with quality scores≤5, the pooled proportion of travel history was 31.7% (95%CI: 12.3%, 51.1%) which was lower than the original results.
10. Estimation of proportion of homosexual: After excluding 4 studies with quality scores≤5, the pooled proportion of homosexual was 82.5% (95%CI: 65.6%, 99.3%) which was slightly higher than the original results.

**
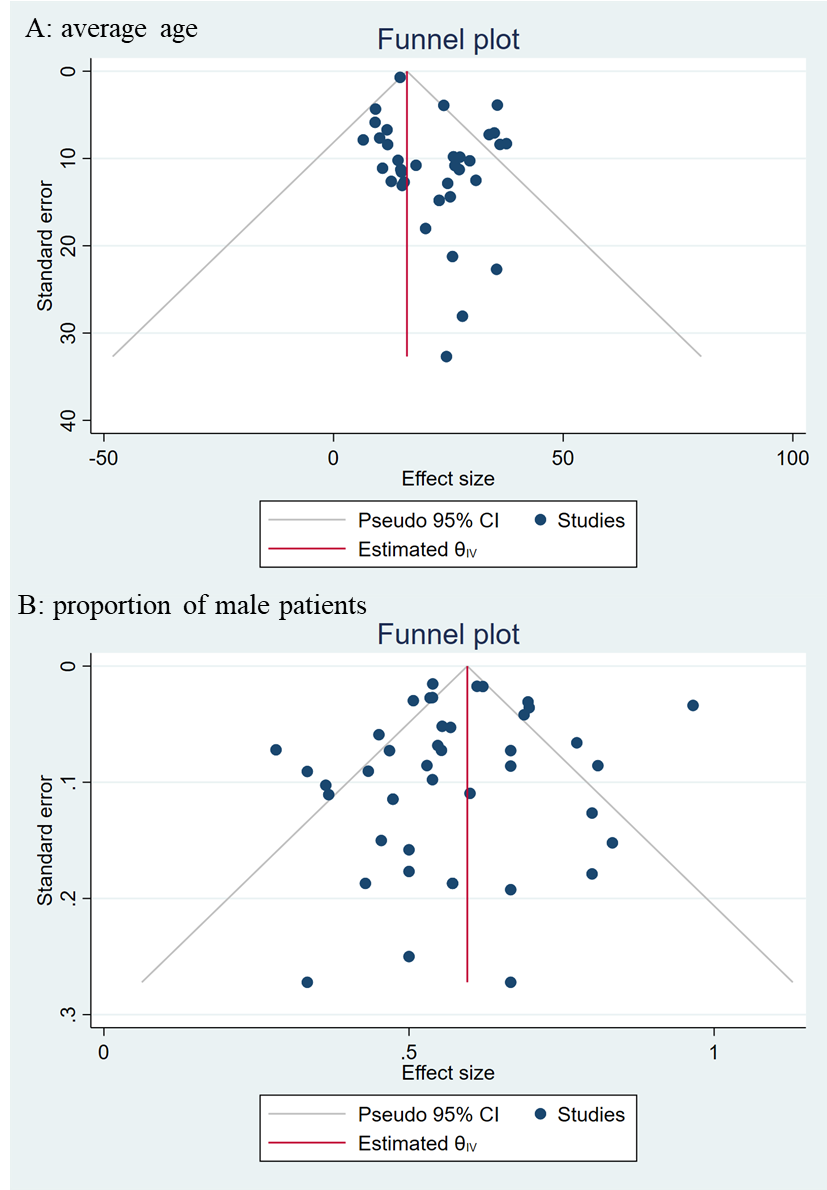
**

**Figure S13: Funnel plots for the estimation of average age and proportion of male patients (Global. 2022)**

**
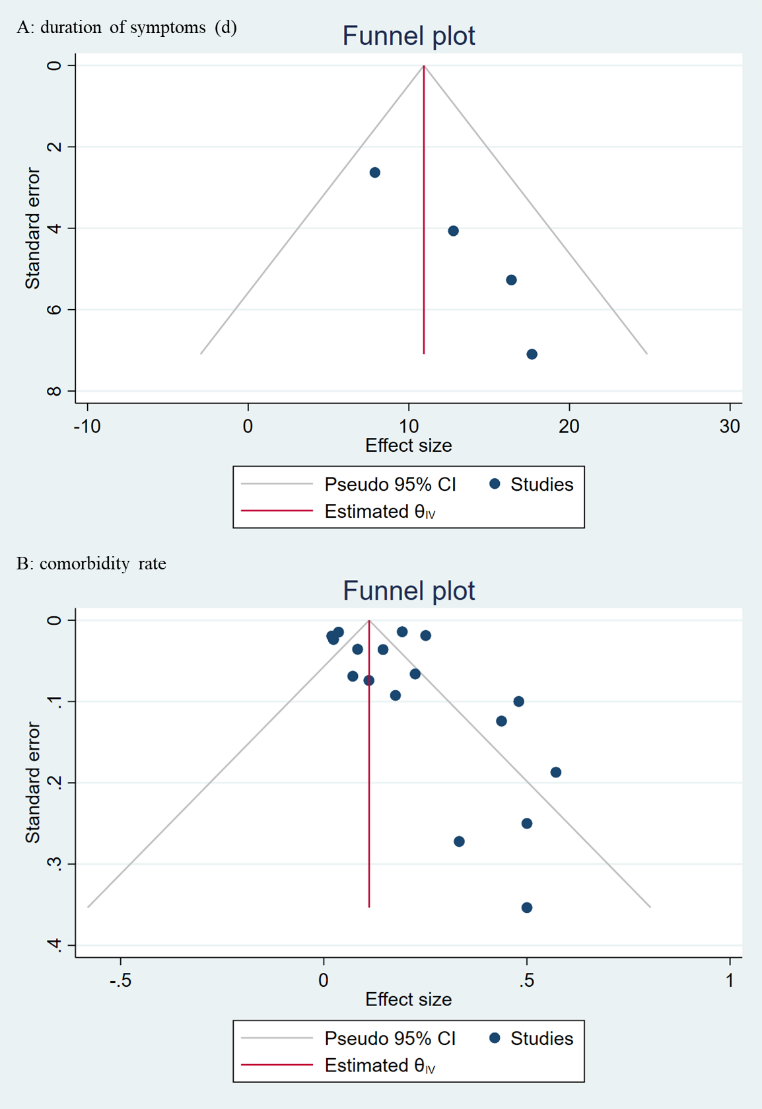
**

**Figure S14: Funnel plots for the estimation of duration of symptoms (days) and comorbidity rate (Global. 2022)**

**
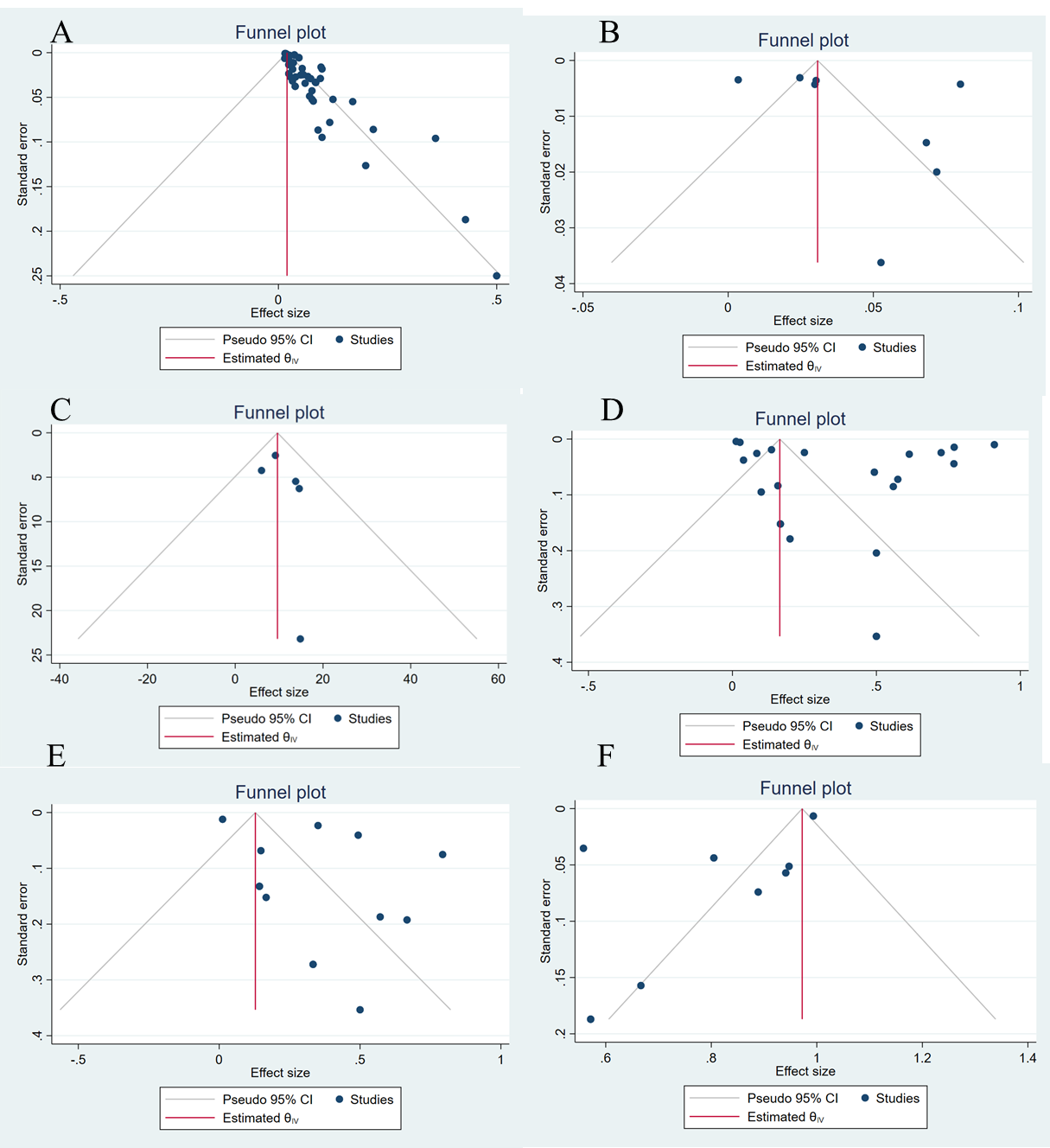
**

**Figure S15: Funnel plots for the estimation of epidemiological characteristics of monkeypox cases (Global. 2022)**

**A: Case fatality rate; B: Secondary attack rate; C: Average incubation period (days); D: Proportion of animal contact history; E: Proportion of travel history; F: Proportion of homosexual.**

**
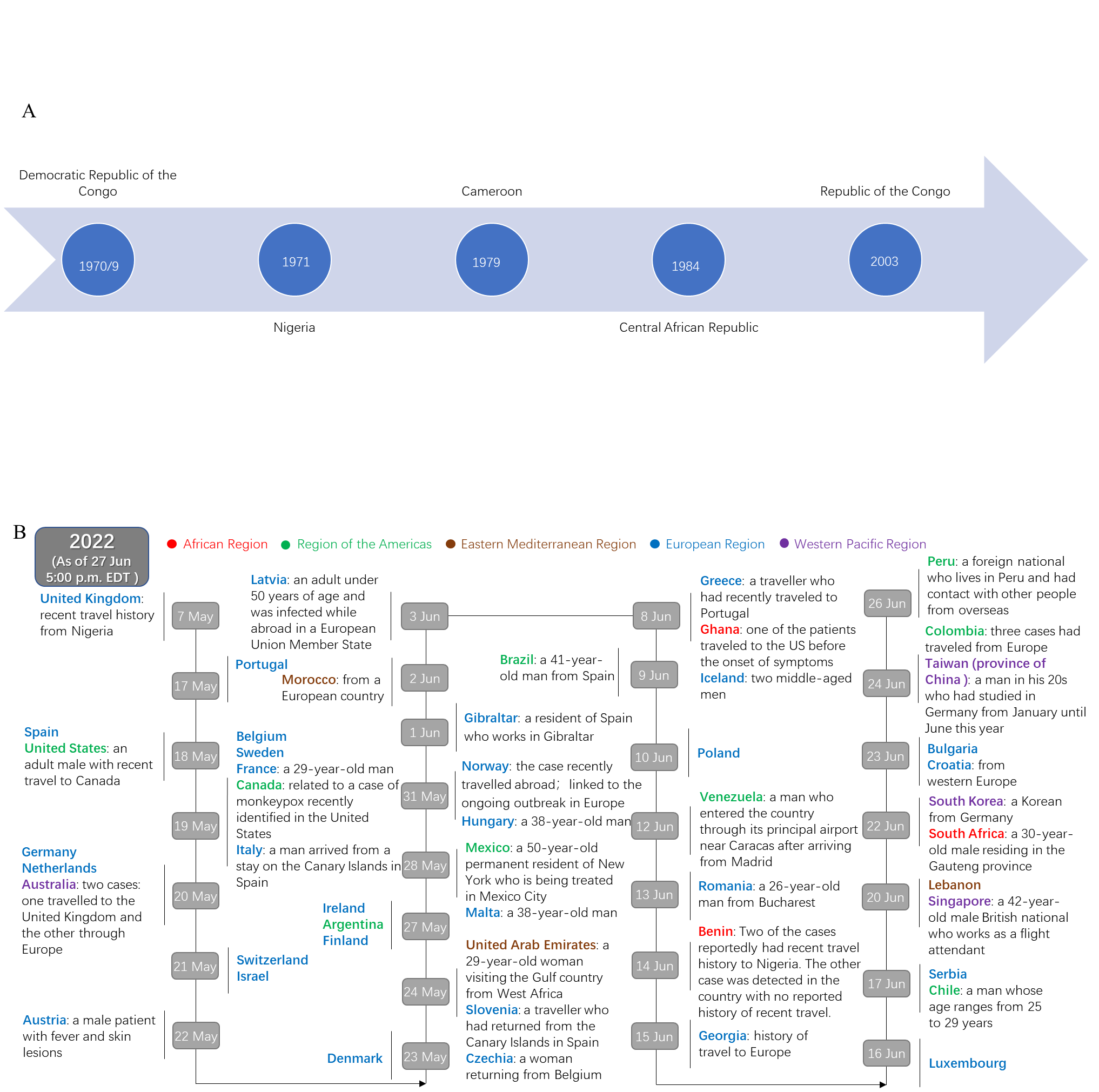
**

**Figure S16: The progression of monkeypox epidemic in countries or territories (Global. 2022)**

**A: major endemic countries before 2022 May; B: the reported countries from 7 May, 2022 to 16 June, 2022.**

**Table S20: The specific information sources of the history of mpox epidemic (Global. 2022)**

| Countries or territories | Confirmed date |  | Features |  | Source |
| --- | --- | --- | --- | --- | --- |
| United Kingdom | 2022/5/7 |  | ①has a recent travel history from Nigeria; ②Initial symptoms include fever, headache, muscle aches, backache, swollen lymph nodes, chills and exhaustion. A rash can develop, often beginning on the face, then spreading to other parts of the body. The rash changes and goes through different stages before finally forming a scab, which later falls off. | | [https://www.gov.uk/government/news/monkeypox-cases-confirmed-in-england-latest-updates#full-publication-update-history](https://www.gov.uk/government/news/monkeypox-cases-confirmed-in-england-latest-updates) |
| Portugal | 2022/5/17 |  | not mentioned | | <https://baijiahao.baidu.com/s?id=1733150000798260054&wfr=baike> |
| Spain | 2022/5/18 |  | not mentioned | | <https://content-static.cctvnews.cctv.com/snow-book/index.html?item_id=3267154065762020641&toc_style_id=feeds_default&share_to=qq&track_id=e3bd93d5-fbc8-427d-b884-aa6dccac7e10> |
| United States | 2022/5/18 |  | an adult male with recent travel to Canada | | <https://www.mass.gov/news/massachusetts-public-health-officials-confirm-case-of-monkeypox> |
| France | 2022/5/19 |  | ①a 29-year-old man  ②with no history of traveling to a country where the virus is circulating | | <https://www.santepubliquefrance.fr/presse/2022/un-premier-cas-confirme-de-monkeypox-sur-le-territoire-national> |
| Canada | 2022/5/19 |  | ①two cases ②related to a case of monkeypox recently identified in the United States who travelled to Canada from the U.S. | | <https://www.canada.ca/en/public-health/news/2022/05/public-health-agency-of-canada-confirms-2-cases-of-monkeypox.html> |
| Italy | 2022/5/19 |  | a man arrived from a stay on the Canary Islands in Spain | | <https://baijiahao.baidu.com/s?id=1733257741866294941&wfr=spider&for=pc> |
| Belgium | 2022/5/19 |  | not mentioned | | <https://www.itg.be/E/Article/institute-of-tropical-medicine-confirms-first-case-of-monkeypox-in-belgium> |
| Sweden | 2022/5/19 |  | not mentioned | | <https://www.thelocal.se/20220519/sweden-records-first-proven-monkeypox-infection/> |
| Germany | 2022/5/20 |  | not mentioned | | <https://www.reuters.com/business/healthcare-pharmaceuticals/germany-detects-first-case-monkeypox-military-medical-service-2022-05-20/> |
| Netherlands | 2022/5/20 |  | not mentioned | | <https://www.government.nl/latest/news/2022/05/24/monkeypox-cases-must-be-reported> |
| Australia | 2022/5/20 |  | two cases：one travelled to the United Kingdom and the other through Europe. | | <https://www.health.gov.au/news/monkeypox-update-from-acting-cmo-dr-sonya-bennett> |
| Switzerland | 2022/5/21 |  | not mentioned | | <https://www.bag.admin.ch/bag/de/home/krankheiten/ausbrueche-epidemien-pandemien/aktuelle-ausbrueche-epidemien/affenpocken.html> |
| Israel | 2022/5/21 |  | not mentioned | | <https://www.gov.il/en/departments/news/21052021-02> |
| Austria | 2022/5/22 |  | a male patient with fever and skin lesions | | <https://www.reuters.com/world/europe/austrias-first-case-monkeypox-confirmed-vienna-health-authority-says-2022-05-22/> |
| Denmark | 2022/5/23 |  | an adult male who had returned from a trip to Spain | | <https://www.reuters.com/world/europe/denmark-registers-first-case-monkeypox-infection-health-ministry-2022-05-23/> |
| United Arab Emirates | 2022/5/24 |  | a 29-year-old woman visiting the Gulf country from West Africa | | <https://www.reuters.com/business/healthcare-pharmaceuticals/uae-announces-first-case-monkeypox-country-wam-2022-05-24/> |
| Slovenia | 2022/5/24 |  | a traveller who had returned from the Canary Islands in Spain | | <https://www.reuters.com/world/europe/slovenia-reports-first-case-monkeypox-infection-media-2022-05-24/> |
| Czechia | 2022/5/24 |  | ①a woman returning from a festival in Belgium ②fever and rash | | <https://www.seznamzpravy.cz/clanek/domaci-prvni-pacient-s-opicimi-nestovicemi-je-v-cesku-203532> |
| Ireland | 2022/5/27 |  | not mentioned | | <https://www.bbc.com/news/articles/cqlvpk00k42o> |
| Argentina | 2022/5/27 |  | two cases |  | <https://www.reuters.com/world/americas/argentina-confirms-latin-americas-first-case-monkeypox-2022-05-27/> |
| Finland | 2022/5/27 |  | not mentioned | | <https://www.reuters.com/world/europe/first-case-monkeypox-confirmed-finland-2022-05-27/> |
| Mexico | 2022/5/28 |  | a 50-year-old permanent resident of New York who is being treated in Mexico City | | <https://www.reuters.com/world/americas/mexico-confirms-first-case-monkeypox-health-official-2022-05-28/> |
| Malta | 2022/5/28 |  | a 38-year-old man | | <https://www.gov.mt/en/Government/DOI/Press%20Releases/Pages/2022/05/28/pr220711.aspx> |
| Hungary | 2022/5/31 |  | a 38-year-old man | | https://www.reuters.com/world/europe/hungary-reports-first-case-monkeypox-2022-05-31/ |
| Norway | 2022/5/31 |  | The infected person had recently travelled abroad, and the case is linked to the ongoing outbreak in Europe | | <https://www.fhi.no/en/news/2022/one-person-with-confirmed-monkeypox-in-norway/> |
| Gibraltar | 2022/6/1 |  | a resident of Spain who works in Gibraltar | | https://www.chronicle.gi/gha-confirms-first-case-of-monkeypox-in-gibraltar/ |
| Morocco | 2022/6/2 |  | from a European country | | <https://moroccolatestnews.com/morocco-records-1-first-case-of-monkeypox/> |
| Latvia | 2022/6/3 |  | an adult under 50 years of age and was infected while abroad in a European Union Member State | | <https://eng.lsm.lv/article/society/health/latvia-gets-first-case-of-monkeypox.a459889/> |
| Greece | 2022/6/8 |  | a traveller who had recently traveled to Portugal | | <https://www.reuters.com/world/europe/greece-detects-first-case-monkeypox-infection-2022-06-08/> |
| Ghana | 2022/6/8 |  | 5 cases：one of the patients traveled to the US before the onset of symptoms | | <https://crisis24.garda.com/alerts/2022/06/ghana-officials-report-confirmed-monkeypox-cases-in-eastern-greater-accra-and-western-regions-as-of-early-june-low-risk-of-transmission> |
| Brazil | 2022/6/9 |  | ①a 41-year-old man ②from Spain ③fever,diarrhea,muscle aches | | <https://www.gov.br/saude/pt-br/canais-de-atendimento/sala-de-imprensa/notas-a-imprensa/2022/brasil-confirma-primeiro-caso-de-monkeypox> |
| Poland | 2022/6/10 |  | not mentioned | | <https://www.gov.pl/web/wsse-warszawa/ospa-malpia--podstawowe-informacje> |
| Venezuela | 2022/6/12 |  | a man who entered the country through its principal airport near Caracas after arriving from Madrid | | <https://www.reuters.com/business/healthcare-pharmaceuticals/venezuela-confirms-first-case-monkeypox-2022-06-12/> |
| Romania | 2022/6/13 |  | a 26-year-old man from Bucharest | | <https://www.ms.ro/2022/06/13/primul-caz-de-variola-maimutei-diagnosticat-in-romania/> |
| Benin | 2022/6/14 |  | 3 cases：Two of the cases reportedly had recent travel history to Nigeria. The other case was detected in the North of the country with no reported history of recent travel. | | <http://outbreaknewstoday.com/africa-benin-reports-3-monkeypox-cases-16828/> |
| Georgia | 2022/6/15 |  | history of travel to Europe | | <https://agenda.ge/en/news/2022/2279> |
| Luxembourg | 2022/6/16 |  | not mentioned | | <https://www.luxtimes.lu/en/luxembourg/first-case-of-monkeypox-detected-in-luxembourg-62aaebfede135b92360305db> |
| Chile | 2022/6/17 |  | a man whose age ranges from 25 to 29 years | | <https://www.minsal.cl/casos-confirmados-viruela-del-mono/> |
| Serbia | 2022/6/17 |  | not mentioned | | <https://www.reuters.com/business/healthcare-pharmaceuticals/serbia-reports-first-case-monkeypox-2022-06-17/> |
| Lebanon | 2022/6/20 |  | not mentioned | | <https://www.moph.gov.lb/en/Pages/17/62474/the-moph-confirms-first-case-of-monkeypox-in-lebanon> |
| Singapore | 2022/6/20 |  | ①a 42-year-old male British national who works as a flight attendant ②The case had onset of headache on 14 June, and fever on 16 June,and he then developed skin rashes on 19 June | | <https://www.moh.gov.sg/news-highlights/details/confirmed-imported-case-of-monkeypox-in-singapore2022> |
| South Korea | 2022/6/22 |  | a Korean from Germany | | <https://www.reuters.com/world/asia-pacific/skorea-reports-first-two-monkeypox-cases-yonhap-2022-06-21/> |
| South Africa | 2022/6/22 |  | ①a 30-year-old male residing in the Gauteng province ②no recent travel history | | <https://www.nicd.ac.za/monkeypox-case-identified-in-south-africa/> |
| Bulgaria | 2022/6/23 |  | two men |  | <https://www.reuters.com/business/healthcare-pharmaceuticals/bulgaria-reports-first-monkeypox-cases-2022-06-23/> |
| Croatia | 2022/6/23 |  | from western Europe | | <https://www.hzjz.hr/sluzba-epidemiologija-zarazne-bolesti/potvrden-prvi-slucaj-majmunskih-boginja-u-hrvatskoj/> |
| Colombia | 2022/6/24 |  | three cases had traveled from Europe | | <https://www.reuters.com/business/healthcare-pharmaceuticals/colombia-reports-first-cases-monkeypox-2022-06-24/> |
| Taiwan (province of China ) | 2022/6/24 |  | ①a man in his 20s who had studied in Germany from January until June this year ②On June 20 he developed symptoms including a fever, sore throat and a rash | | <https://www.reuters.com/business/healthcare-pharmaceuticals/taiwan-confirms-first-imported-case-monkeypox-2022-06-24/> |
| Peru | 2022/6/26 |  | a foreign national who lives in Peru and had contact with other people from overseas | | <https://andina.pe/ingles/noticia-health-minister-confirms-first-monkeypox-case-in-peru-898961.aspx> |
| Iceland | 2022/6/8 confirmed date was unknown） | | two middle-aged men | | <https://icelandmonitor.mbl.is/news/news/2022/06/09/first_cases_of_monkeypox_reported_in_iceland/> |
| Democratic Republic of the Congo | 1970 |  |  | | ① Durski KN, McCollum AM, Nakazawa Y, Petersen BW, Reynolds MG, Briand S, Djingarey MH, Olson V, Damon IK, Khalakdina A. Emergence of Monkeypox - West and Central Africa, 1970-2017. MMWR Morb Mortal Wkly Rep. 2018 Mar 16;67(10):306-310. doi: 10.15585/mmwr.mm6710a5. Erratum in: MMWR Morb Mortal Wkly Rep. 2018 Apr 27;67(16):479. PMID: 29543790; PMCID: PMC5857192.[85] ② Ladnyj ID, Ziegler P, Kima E. A human infection caused by monkeypox virus in Basankusu Territory, Democratic Republic of the Congo. Bull World Health Organ. 1972;46(5):593-7. PMID: 4340218; PMCID: PMC2480792.[86] |
| Nigeria | 1971 |  |  | |  |
| Cameroon | 1979 |  |  | |  |
| Central African Republic | 1984 |  |  | |  |
| Republic of the Congo | 2003 |  |  | |  |

**Table S21: The original values of confirmed cases in 2022 and healthcare access and quality index in 2015, human development index in 2019, sociodemographic index in 2019, and international total arrivals in 2020 among the 55 countries (Global. 2022)**

| Updated date | Countries or territories | Confirmed cases | WHO region | World bank income level | Population 2020 | HIQ 2015 | HDI 2019 | SDI 2019 | International travel arrivals 2019 (thousand) |
| --- | --- | --- | --- | --- | --- | --- | --- | --- | --- |
| 2022/6/22 | Benin | 3 | African Region | Lower middle income | 12123198 | 43 | 0.545 | 0·352 | 337 |
| 2022/6/22 | Cameroon | 3 | African Region | Lower middle income | 26545864 | 44.4 | 0.563 | 0·49 | 997 |
| 2022/6/22 | Central African Republic | 8 | African Region | Low income | 4829764 | 28.6 | 0.397 | 0·274 | 109 |
| 2022/6/22 | Congo, Rep. | 2 | African Region | Lower middle income | 5518092 | 43.5 | 0.574 | 0.57 | 158 |
| 2022/6/22 | Congo, Dem. Rep. | 10 | African Region | Low income | 89561404 | 40.4 | 0.48 | 0.38 | 351 |
| 2022/6/22 | Ghana | 5 | African Region | Lower middle income | 31072945 | 49.7 | 0.611 | 0·557 | 897 |
| 2022/6/22 | Nigeria | 41 | African Region | Lower middle income | 2.06E+08 | 51.3 | 0.539 | 0·515 | 5265 |
| 2022/6/27 | South Africa | 1 | African Region | Upper middle income | 59308690 | 52 | 0.709 | 0·678 | 14797 |
| 2022/6/27 | Lebanon | 1 | Eastern Mediterranean Region | Upper middle income | 6825442 | 80 | 0.744 | 0·708 | 1964 |
| 2022/6/27 | Morocco | 1 | Eastern Mediterranean Region | Lower middle income | 36910558 | 61.3 | 0.686 | 0·548 | 12489 |
| 2022/6/27 | United Arab Emirates | 13 | Eastern Mediterranean Region | High income | 9890400 | 72.2 | 0.89 | 0·88 | 21600 |
| 2022/6/27 | Austria | 20 | European Region | High income | 8916864 | 88.2 | 0.922 | 0·849 | 31900 |
| 2022/6/27 | Belgium | 77 | European Region | High income | 11544241 | 87.9 | 0.931 | 0·851 | 9300 |
| 2022/6/27 | Bulgaria | 2 | European Region | Upper middle income | 6934015 | 71.4 | 0.816 | 0·764 | 12552 |
| 2022/6/27 | Croatia | 1 | European Region | High income | 4047680 | 81.6 | 0.851 | 0·794 | 60021 |
| 2022/6/27 | Czech Republic | 7 | European Region | High income | 10697858 | 84.8 | 0.9 | 0·828 | 36,268 |
| 2022/6/27 | Denmark | 16 | European Region | High income | 5831404 | 85.7 | 0.94 | 0·89 | 31608 |
| 2022/6/27 | Finland | 4 | European Region | High income | 5529543 | 89.6 | 0.938 | 0·856 | 3300 |
| 2022/6/27 | France | 330 | European Region | High income | 67379908 | 87.9 | 0.901 | 0·834 | 211,998 |
| 2022/6/27 | Georgia | 1 | European Region | Upper middle income | 3722716 | 62.1 | 0.812 | 0·702 | 7726 |
| 2022/6/27 | Germany | 765 | European Region | High income | 83160871 | 86.4 | 0.947 | 0·898 | 39600 |
| 2022/6/27 | Gibraltar | 1 | European Region | High income | 33691 |  |  |  |  |
| 2022/6/27 | Greece | 3 | European Region | High income | 10700556 | 87 | 0.888 | 0·794 | 34005 |
| 2022/6/27 | Hungary | 7 | European Region | High income | 9750149 | 79.6 | 0.854 | 0·791 | 61397 |
| 2022/6/27 | Iceland | 3 | European Region | High income | 366463 | 93.6 | 0.949 | 0·869 | 2202 |
| 2022/6/27 | Ireland | 28 | European Region | High income | 4985674 | 88.4 | 0.955 | 0·867 | 11000 |
| 2022/6/27 | Israel | 29 | European Region | High income | 9215100 | 85.5 | 0.919 | 0·803 | 4900 |
| 2022/6/27 | Italy | 127 | European Region | High income | 59449527 | 88.7 | 0.892 | 0·801 | 95399 |
| 2022/6/27 | Latvia | 2 | European Region | High income | 1900449 | 77.7 | 0.866 | 0·82 | 8342 |
| 2022/6/27 | Luxembourg | 3 | European Region | High income | 630419 | 89.3 | 0.916 | 0·895 | 1000 |
| 2022/6/27 | Malta | 2 | European Region | High income | 515332 | 85.1 | 0.895 | 0·801 | 3519 |
| 2022/6/27 | Netherlands | 257 | European Region | High income | 17441500 | 89.5 | 0.944 | 0·883 | 20100 |
| 2022/6/27 | Norway | 4 | European Region | High income | 5379475 | 90.5 | 0.957 | 0·913 | 5900 |
| 2022/6/27 | Poland | 7 | European Region | High income | 37899070 | 79.6 | 0.88 | 0·802 | 88500 |
| 2022/6/27 | Portugal | 365 | European Region | High income | 10297081 | 84.5 | 0.864 | 0·743 | 17200 |
| 2022/6/27 | Serbia | 1 | European Region | Upper middle income | 6899126 | 75.4 | 0.806 | 0·767 | 1800 |
| 2022/6/27 | Slovenia | 9 | European Region | High income | 2102419 | 87.4 | 0.917 | 0·84 | 4425 |
| 2022/6/27 | Spain | 736 | European Region | High income | 47363419 | 89.6 | 0.904 | 0·767 | 126170 |
| 2022/6/27 | Sweden | 13 | European Region | High income | 10353442 | 90.5 | 0.945 | 0·872 | 7600 |
| 2022/6/27 | Switzerland | 68 | European Region | High income | 8636561 | 91.8 | 0.955 | 0·929 | 11800 |
| 2022/6/27 | United Kingdom | 910 | European Region | High income | 67215293 | 84.6 | 0.932 | 0.85 | 40857 |
| 2022/6/27 | Romania | 6 | European Region | Upper middle income | 19257520 | 74.4 | 0.828 | 0·76 | 12815 |
| 2022/6/27 | Argentina | 4 | Region of the Americas | Upper middle income | 45376763 | 68.4 | 0.845 | 0·708 | 7400 |
| 2022/6/27 | Brazil | 20 | Region of the Americas | Upper middle income | 2.13E+08 | 64.9 | 0.765 | 0·64 | 6400 |
| 2022/6/27 | Canada | 235 | Region of the Americas | High income | 38037204 | 87.6 | 0.929 | 0·873 | 32430 |
| 2022/6/27 | Chile | 6 | Region of the Americas | High income | 19116209 | 76 | 0.851 | 0·759 | 5431 |
| 2022/6/27 | Colombia | 3 | Region of the Americas | Upper middle income | 50882884 | 67.8 | 0.767 | 0·633 | 4529 |
| 2022/6/27 | Mexico | 11 | Region of the Americas | Upper middle income | 1.29E+08 | 62.6 | 0.779 | 0·649 | 97400 |
| 2022/6/27 | Peru | 1 | Region of the Americas | Upper middle income | 32971846 | 69.6 | 0.777 | 0·648 | 5300 |
| 2022/6/27 | United States | 243 | Region of the Americas | High income | 3.32E+08 | 81.3 | 0.926 | 0.86 | 166009 |
| 2022/6/27 | Venezuela, RB | 1 | Region of the Americas | not calssfied | 28435943 | 64.7 | 0.711 | 0.61 | 429 |
| 2022/6/27 | Australia | 10 | Western Pacific Region | High income | 25693267 | 89.8 | 0.944 | 0·839 | 9500 |
| 2022/6/27 | Singapore | 1 | Western Pacific Region | High income | 5685807 | 86.3 | 0.938 | 0·861 | 19116 |
| 2022/6/27 | Korea, Rep. | 1 | Western Pacific Region | High income | 51836239 | 85.8 | 0.916 | 0.88 | 17503 |
| 2022/6/27 | Taiwan (province of China) | 1 | Western Pacific Region | Upper middle income | | 77.6 |  | 0·868 | 11864 |

HAQ: healthcare access and quality index; HDI: human development index; SDI: sociodemographic index.

**
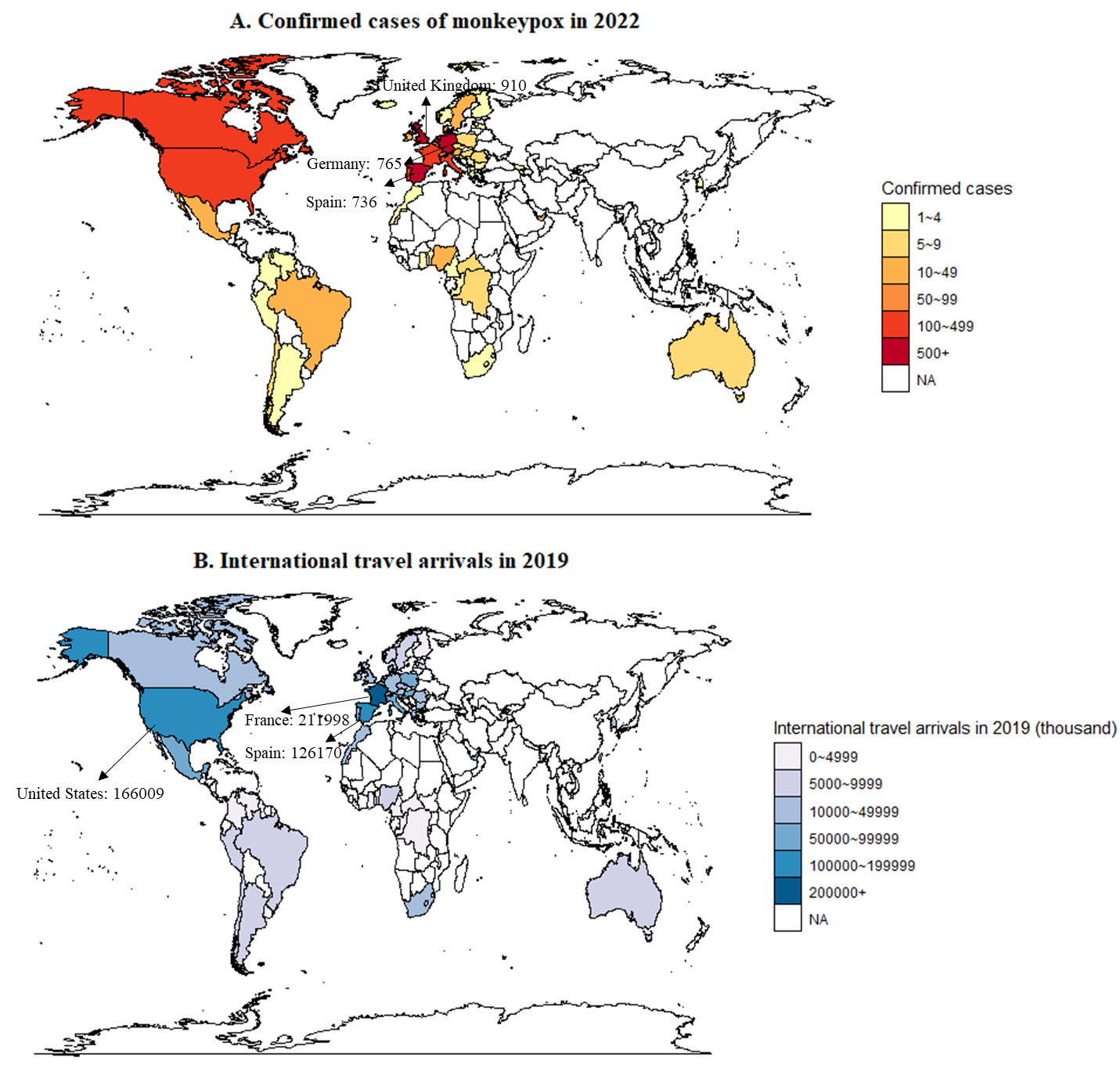
Figure S17: The global map of mpox confirmed cases in 2022 multiple country mpox outbreak and international travel arrivals in 2019 at the country and territorial levels (Global. 2022)**

Confirmed Mpox cases were reported by the WHO and CDC on June 27, 2022. A: confirmed mpox cases; B: international travel arrivals. CDC: center for disease control and prevention; WHO: World Health Organization.

**Box:The changes of symptoms and sites of rash in 2022 multiple country monkeypox outbreak, compare with that before 2022 (Global. 2022)**

| Author | Country | Date of cases | Sample size | The top three symptoms (%, n) | Sites of rash (%, n) | Men who have sex with men (MSM) (%, n) |
| --- | --- | --- | --- | --- | --- | --- |
| Girometti, Nicolò | London, UK | between May 14 and May 25, 2022 | 54 | rash (100%, 54/54); fatigue, asthenia, or lethargy (67%,36/54); fever (57%, 31/54) | genital lesions (61%, 33/54); perianal lesions (44%, 24/54), lesions on limbs (50%, 27/54)……facial lesions (20%, 11/54) | (100%, 54/54) |
| Gemma Hobson | Nigeria | between Sept 22, 2017, and Sept 16, 2018 | 122 (confirmed or probable cases) | vesiculopustular rash (100%, 122 /122) fever (88%, 81 /92); headache (79%,61/77); pruritus (73%, 57/78) | face (96%, 68/71), legs (91%, 63/69), trunk (80%, 56/70)……genitalia (68%, 44/65) | Not mentioned |
| Erin R. Whitehouse | Tshuapa Province, Democratic Republic of the Congo | 2011–2015 | 1057 | rash (100%, 1057/1057); subjective or measured fever before rash onset (94%, 1023/1029); lymphadenopathy (84.7%, 876/1034) | face (98%, 1036/1057), trunk（97.3%,, 1028/1057）, arms（97.1%, , 1026/1057）……genitals (28.4%, 300/1057)~ | Not mentioned, but the proportion of animal contact was 36.9% (36.9%, 309/837) |
| Lynda Osadebe | Democratic Republic of the Congo | 2009–2014 | 333 | fever (100%, 329/329), febrile prodrome (99.1%, 327/330), rash(95.2%,316/332), | face (99.1%, 330/333), thorax (98.5%, 328/333),arms(98.2%, 326/332)......genitals (28.2%, 87/309） | Not mentioned |
| Gregory D. Huhn | United States | 2003 | 34 | rash（97%）, fever（85%）, chills and adenopathy（71%） | arms and/or hands (81.3%, 26/32), legs and/or feet（65.6%, 21/32）, head and/or neck（62.5%, 20/32）...... mucosa (including ocular)（6.3%, 2/32） | Not mentioned, but the proportion of animal contact was 56%( 56%,19/34) |


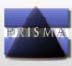
**PRISMA 2020 Checklist**

| **Section and Topic** | **Item #** | **Checklist item** | **Location where item is reported** |
| --- | --- | --- | --- |
| **TITLE** | | |  |
| Title | 1 | Identify the report as a systematic review. | 1 |
| **ABSTRACT** | | |  |
| Abstract | 2 | See the PRISMA 2020 for Abstracts checklist. | 1 |
| **INTRODUCTION** | | |  |
| Rationale | 3 | Describe the rationale for the review in the context of existing knowledge. | 2-3 |
| Objectives | 4 | Provide an explicit statement of the objective(s) or question(s) the review addresses. | 2-3 |
| **METHODS** | | |  |
| Eligibility criteria | 5 | Specify the inclusion and exclusion criteria for the review and how studies were grouped for the syntheses. | 3 |
| Information sources | 6 | Specify all databases, registers, websites, organisations, reference lists and other sources searched or consulted to identify studies. Specify the date when each source was last searched or consulted. | 3-4 |
| Search strategy | 7 | Present the full search strategies for all databases, registers and websites, including any filters and limits used. | 3-4 |
| Selection process | 8 | Specify the methods used to decide whether a study met the inclusion criteria of the review, including how many reviewers screened each record and each report retrieved, whether they worked independently, and if applicable, details of automation tools used in the process. | 3-4 |
| Data collection process | 9 | Specify the methods used to collect data from reports, including how many reviewers collected data from each report, whether they worked independently, any processes for obtaining or confirming data from study investigators, and if applicable, details of automation tools used in the process. | 3-4 |
| Data items | 10a | List and define all outcomes for which data were sought. Specify whether all results that were compatible with each outcome domain in each study were sought (e.g. for all measures, time points, analyses), and if not, the methods used to decide which results to collect. | 3 |
|  | 10b | List and define all other variables for which data were sought (e.g. participant and intervention characteristics, funding sources). Describe any assumptions made about any missing or unclear information. | 3 |
| Study risk of bias assessment | 11 | Specify the methods used to assess risk of bias in the included studies, including details of the tool(s) used, how many reviewers assessed each study and whether they worked independently, and if applicable, details of automation tools used in the process. | 4 |
| Effect measures | 12 | Specify for each outcome the effect measure(s) (e.g. risk ratio, mean difference) used in the synthesis or presentation of results. | 4 |
| Synthesis methods | 13a | Describe the processes used to decide which studies were eligible for each synthesis (e.g. tabulating the study intervention characteristics and comparing against the planned groups for each synthesis (item #5)). | 4 |
|  | 13b | Describe any methods required to prepare the data for presentation or synthesis, such as handling of missing summary statistics, or data conversions. | 4 |
|  | 13c | Describe any methods used to tabulate or visually display results of individual studies and syntheses. | 4 |
|  | 13d | Describe any methods used to synthesize results and provide a rationale for the choice(s). If meta-analysis was performed, describe the model(s), method(s) to identify the presence and extent of statistical heterogeneity, and software package(s) used. | 4 |
|  | 13e | Describe any methods used to explore possible causes of heterogeneity among study results (e.g. subgroup analysis, meta-regression). | 4-5 |
|  | 13f | Describe any sensitivity analyses conducted to assess robustness of the synthesized results. | 4-5 |
| Reporting bias assessment | 14 | Describe any methods used to assess risk of bias due to missing results in a synthesis (arising from reporting biases). | N/A |
| Certainty assessment | 15 | Describe any methods used to assess certainty (or confidence) in the body of evidence for an outcome. | 4 |
| **RESULTS** | | |  |
| Study selection | 16a | Describe the results of the search and selection process, from the number of records identified in the search to the number of studies included in the review, ideally using a flow diagram. | 6 |
|  | 16b | Cite studies that might appear to meet the inclusion criteria, but which were excluded, and explain why they were excluded. | N/A |
| Study characteristics | 17 | Cite each included study and present its characteristics. | Appendix 3-75 |
| Risk of bias in studies | 18 | Present assessments of risk of bias for each included study. | Appendix 3-75 and page 6-9 |
| Results of individual studies | 19 | For all outcomes, present, for each study: (a) summary statistics for each group (where appropriate) and (b) an effect estimate and its precision (e.g. confidence/credible interval), ideally using structured tables or plots. | Appendix 3-75 and page 17-21 |
| Results of syntheses | 20a | For each synthesis, briefly summarise the characteristics and risk of bias among contributing studies. | page 6-9 |
|  | 20b | Present results of all statistical syntheses conducted. If meta-analysis was done, present for each the summary estimate and its precision (e.g. confidence/credible interval) and measures of statistical heterogeneity. If comparing groups, describe the direction of the effect. | page 6-9 |
|  | 20c | Present results of all investigations of possible causes of heterogeneity among study results. | N/A |
|  | 20d | Present results of all sensitivity analyses conducted to assess the robustness of the synthesized results. | Appendix 3-75 |
| Reporting biases | 21 | Present assessments of risk of bias due to missing results (arising from reporting biases) for each synthesis assessed. | N/A |
| Certainty of evidence | 22 | Present assessments of certainty (or confidence) in the body of evidence for each outcome assessed. | page 6-9 |
| **DISCUSSION** | | |  |
| Discussion | 23a | Provide a general interpretation of the results in the context of other evidence. | 9 |
|  | 23b | Discuss any limitations of the evidence included in the review. | 11 |
|  | 23c | Discuss any limitations of the review processes used. | 9 |
|  | 23d | Discuss implications of the results for practice, policy, and future research. | 11-12 |
| **OTHER INFORMATION** | | |  |
| Registration and protocol | 24a | Provide registration information for the review, including register name and registration number, or state that the review was not registered. | 5 |
|  | 24b | Indicate where the review protocol can be accessed, or state that a protocol was not prepared. | 5 |
|  | 24c | Describe and explain any amendments to information provided at registration or in the protocol. | N/A |
| Support | 25 | Describe sources of financial or non-financial support for the review, and the role of the funders or sponsors in the review. | 12 |
| Competing interests | 26 | Declare any competing interests of review authors. | 12 |
| Availability of data, code and other materials | 27 | Report which of the following are publicly available and where they can be found: template data collection forms; data extracted from included studies; data used for all analyses; analytic code; any other materials used in the review. | 3-4 |

*From:*  Page MJ, McKenzie JE, Bossuyt PM, Boutron I, Hoffmann TC, Mulrow CD, et al. The PRISMA 2020 statement: an updated guideline for reporting systematic reviews. BMJ 2021;372:n71. doi: 10.1136/bmj.n71

**Reference**

1. Isidro J, Borges V, Pinto M, Sobral D, Santos JD, Nunes A, Mixao V, Ferreira R, Santos D, Duarte S *et al*: **Phylogenomic characterization and signs of microevolution in the 2022 multi-country outbreak of monkeypox virus**. *Nat Med* 2022.

2. WHO. Surveillance, case investigation and contact tracing for monkeypox: interim guidance, 24 June 2022. Avalible from https://www.who.int/publications/i/item/WHO-MPX-Surveillance-2022.2

3. Vivancos R, Anderson C, Blomquist P, Balasegaram S, Bell A, Bishop L, Brown CS, Chow Y, Edeghere O, Florence I *et al*: **Community transmission of monkeypox in the United Kingdom, April to May 2022**. *Euro Surveill* 2022, **27**(22).

4. Perez Duque M, Ribeiro S, Martins JV, Casaca P, Leite PP, Tavares M, Mansinho K, Duque LM, Fernandes C, Cordeiro R *et al*: **Ongoing monkeypox virus outbreak, Portugal, 29 April to 23 May 2022**. *Euro Surveill* 2022, **27**(22).

5. Whitehouse ER, Bonwitt J, Hughes CM, Lushima RS, Likafi T, Nguete B, Kabamba J, Monroe B, Doty JB, Nakazawa Y *et al*: **Clinical and Epidemiological Findings from Enhanced Monkeypox Surveillance in Tshuapa Province, Democratic Republic of the Congo during 2011-2015**. *Journal of Infectious Diseases* 2021, **223**(11):1870-1878.

6. Hughes CM, Liu L, Davidson WB, Radford KW, Wilkins K, Monroe B, Metcalfe MG, Likafi T, Lushima RS, Kabamba J *et al*: **A Tale of Two Viruses: Coinfections of Monkeypox and Varicella Zoster Virus in the Democratic Republic of Congo**. *American Journal of Tropical Medicine and Hygiene* 2021, **104**(2):604-611.

7. Ogoina D, Iroezindu M, James HI, Oladokun R, Yinka-Ogunleye A, Wakama P, Otike-Odibi B, Usman LM, Obazee E, Aruna O *et al*: **Clinical Course and Outcome of Human Monkeypox in Nigeria**. *Clinical infectious diseases : an official publication of the Infectious Diseases Society of America* 2020, **71**(8):e210-e214.

8. Ibegu M, Numbere TW, Balogun M, Nguku P: **Descriptive epidemiology of Monkeypox outbreak in Bayelsa State South-South Nigeria, November 2017**. *International Journal of Infectious Diseases* 2020, **101**:255.

9. Doshi RH, Alfonso VH, Morier D, Hoff NA, Sinai C, Mulembakani P, Kisalu N, Cheng A, Ashbaugh H, Gadoth A *et al*: **Monkeypox Rash Severity and Animal Exposures in the Democratic Republic of the Congo**. *Ecohealth* 2020, **17**(1):64-73.

10. Reynolds MG, Wauquier N, Li Y, Satheshkumar PS, Kanneh LD, Monroe B, Maikere J, Saffa G, Gonzalez JP, Fair J *et al*: **Human Monkeypox in Sierra Leone after 44-Year Absence of Reported Cases**. *Emerging infectious diseases* 2019, **25**(5):1023-1025.

11. Sadeuh-Mba SA, Yonga MG, Els M, Batejat C, Eyangoh S, Caro V, Etoundi A, Carniel E, Njouom R: **Monkeypox virus phylogenetic similarities between a human case detected in Cameroon in 2018 and the 2017-2018 outbreak in Nigeria**. *Infection, genetics and evolution : journal of molecular epidemiology and evolutionary genetics in infectious diseases* 2019, **69**:8-11.

12. Ogoina D, Izibewule JH, Ogunleye A, Ederiane E, Anebonam U, Neni A, Oyeyemi A, Etebu EN, Ihekweazu C: **The 2017 human monkeypox outbreak in Nigeria—Report of outbreak experience and response in the Niger Delta University Teaching Hospital, Bayelsa State, Nigeria**. *PLoS ONE* 2019, **14**(4).

13. Doshi RH, Guagliardo SAJ, Doty JB, Babeaux AD, Matheny A, Burgado J, Townsend MB, Morgan CN, Satheshkumar PS, Ndakala N *et al*: **Epidemiologic and Ecologic Investigations of Monkeypox, Likouala Department, Republic of the Congo, 2017**. *Emerging infectious diseases* 2019, **25**(2):281-289.

14. Besombes C, Gonofio E, Konamna X, Selekon B, Grant R, Gessain A, Berthet N, Manuguerra JC, Fontanet A, Nakouné E: **Intrafamily Transmission of Monkeypox Virus, Central African Republic, 2018**. *Emerging infectious diseases* 2019, **25**(8):1602-1604.

15. Mauldin MR, McCollum AM, Nakazawa YJ, Mandra A, Whitehouse ER, Davidson W, Zhao H, Gao J, Li Y, Doty J *et al*: **Exportation of Monkeypox Virus From the African Continent**. *The Journal of infectious diseases* 2022, **225**(8):1367-1376.

16. Ferraro F, Caraglia A, Rapiti A, Cereda D, Vairo F, Mattei G, Maraglino F, Rezza G: **Letter to the editor: multiple introductions of MPX in Italy from different geographic areas**. *Euro Surveill* 2022, **27**(23).

17. Amao LK, Olatunji DI, Igbodo G, Okoli SC, Amaechi I, Goni MI, Ehiakhamen O, Aderinola O, Ogunleye A, Ogunbode O *et al*: **Trend and enhanced surveillance of Monkeypox during COVID-19 pandemic in Nigeria**. *Journal of Public Health in Africa* 2022, **13**(1).

18. Kalthan E, Tenguere J, Ndjapou SG, Koyazengbe TA, Mbomba J, Marada RM, Rombebe P, Yangueme P, Babamingui M, Sambella A *et al*: **Investigation of an outbreak of monkeypox in an area occupied by armed groups, Central African Republic**. *Medecine et maladies infectieuses* 2018, **48**(4):263-268.

19. Osadebe L, Hughes CM, Lushima RS, Kabamba J, Nguete B, Malekani J, Pukuta E, Karhemere S, Tamfum J-JM, Okitolonda EW *et al*: **Enhancing case definitions for surveillance of human monkeypox in the Democratic Republic of Congo**. *Plos Neglected Tropical Diseases* 2017, **11**(9).

20. Nakoune E, Lampaert E, Ndjapou SG, Janssens C, Zuniga I, Van Herp M, Fongbia JP, Koyazegbe TD, Selekon B, Komoyo GF *et al*: **A Nosocomial Outbreak of Human Monkeypox in the Central African Republic**. *Open Forum Infectious Diseases* 2017, **4**(4).

21. Mbala PK, Huggins JW, Riu-Rovira T, Ahuka SM, Mulembakani P, Rimoin AW, Martin JW, Muyembe J-JT: **Maternal and Fetal Outcomes Among Pregnant Women With Human Monkeypox Infection in the Democratic Republic of Congo**. *Journal of Infectious Diseases* 2017, **216**(7):824-828.

22. Nolen LD, Osadebe L, Katomba J, Likofata J, Mukadi D, Monroe B, Doty J, Hughes CM, Kabamba J, Malekani J *et al*: **Extended human-to-human transmission during a monkeypox outbreak in the Democratic Republic of the Congo**. *Emerging infectious diseases* 2016, **22**(6):1014-1021.

23. Johnston SC, Johnson JC, Stonier SW, Lin KL, Kisalu NK, Hensley LE, Rimoin AW: **Cytokine modulation correlates with severity of monkeypox disease in humans**. *Journal of clinical virology : the official publication of the Pan American Society for Clinical Virology* 2015, **63**:42-45.

24. Reynolds MG, Emerson G, Pakuta E, Karhemere S, McCollum A, Moses C, Wilkins K, Zhao H, Karem K, Carroll D *et al*: **Detection of human monkeypox in the republic of the congo following intensive community education**. *American Journal of Tropical Medicine and Hygiene* 2012, **87**(5):136.

25. Berthet N, Nakouné E, Whist E, Selekon B, Burguière AM, Manuguerra JC, Gessain A, Kazanji M: **Maculopapular lesions in the Central African Republic**. *Lancet (London, England)* 2011, **378**(9799):1354.

26. Rimoin AW, Mulembakani PM, Johnston SC, Lloyd Smith JO, Kisalu NK, Kinkela TL, Blumberg S, Thomassen HA, Pike BL, Fair JN *et al*: **Major increase in human monkeypox incidence 30 years after smallpox vaccination campaigns cease in the Democratic Republic of Congo**. *Proceedings of the National Academy of Sciences of the United States of America* 2010, **107**(37):16262-16267.

27. Croft DR, Sotir MJ, Williams CJ, Kazmierczak JJ, Wegner MV, Rausch D, Graham MB, Foldy SL, Wolters M, Damon IK *et al*: **Occupational risks during a monkeypox outbreak, Wisconsin, 2003**. *Emerging infectious diseases* 2007, **13**(8):1150-1157.

28. Huhn GD, Bauer AM, Yorita K, Graham MB, Sejvar J, Likos A, Damon IK, Reynolds MG, Kuehnert MJ: **Clinical characteristics of human monkeypox, and risk factors for severe disease**. *Clinical infectious diseases : an official publication of the Infectious Diseases Society of America* 2005, **41**(12):1742-1751.

29. Sejvar JJ, Chowdary Y, Schomogyi M, Stevens J, Patel J, Karem K, Fischer M, Kuehnert MJ, Zaki SR, Paddock CD *et al*: **Human monkeypox infection: a family cluster in the midwestern United States**. *The Journal of infectious diseases* 2004, **190**(10):1833-1840.

30. Centers for Disease C, Prevention: **Multistate outbreak of monkeypox--Illinois, Indiana, and Wisconsin, 2003**. *MMWR Morbidity and mortality weekly report* 2003, **52**(23):537-540.

31. Centers for Disease C, Prevention: **Update: multistate outbreak of monkeypox--Illinois, Indiana, Kansas, Missouri, Ohio, and Wisconsin, 2003**. *MMWR Morbidity and mortality weekly report* 2003, **52**(26):616-618.

32. Meyer H, Perrichot M, Stemmler M, Emmerich P, Schmitz H, Varaine F, Shungu R, Tshioko F, Formenty P: **Outbreaks of disease suspected of being due to human monkeypox virus infection in the Democratic Republic of Congo in 2001**. *Journal of clinical microbiology* 2002, **40**(8):2919-2921.

33. Hutin YJF, Williams RJ, Malfait P, Pebody R, Loparev VN, Ropp SL, Rodriguez M, Knight JC, Tshioko FK, Khan AS *et al*: **Outbreak of human monkeypox, Democratic Republic of Congo, 1996-1997**. *Emerging infectious diseases* 2001, **7**(3):434-438.

34. Breman JG, Kalisa R, Steniowski MV, Zanotto E, Gromyko AI, Arita I: **Human monkeypox, 1970-79**. *Bull World Health Organ* 1980, **58**(2):165-182.

35. Boumandouki P, Bileckot R, Ibara JR, Satounkazi C, Wassa Wassa D, Libama F, Moudzeo H, Bolanda JD, Ngokaba C: **Simian smallpox (or monkey smallpox): Study of 8 cases observed at Impfondo hospital in Republic of Congo**. *Bulletin de la Societe de Pathologie Exotique* 2007, **100**(1):17-21.

36. Kile JC, Fleischauer AT, Beard B, Kuehnert MJ, Kanwal RS, Pontones P, Messersmith HJ, Teclaw R, Karem KL, Braden ZH *et al*: **Transmission of monkeypox among persons exposed to infected prairie dogs in Indiana in 2003**. *Archives of pediatrics & adolescent medicine* 2005, **159**(11):1022-1025.

37. **monkeypox in email promed** [<https://promedmail.org/promed-posts/>]

38. Adler H, Gould S, Hine P, Snell LB, Wong W, Houlihan CF, Osborne JC, Rampling T, Beadsworth MB, Duncan CJ *et al*: **Clinical features and management of human monkeypox: a retrospective observational study in the UK**. *The Lancet Infectious diseases* 2022.

39. Akar S, Adesola YO, Burga J, Oluwafemi B, Akinrogbe J, Ihekweazu C: **Descriptive epidemiology of monkeypox in Nigeria, September 2017–June 2019**. *International Journal of Infectious Diseases* 2020, **101**:219-220.

40. Yinka-Ogunleye A, Aruna O, Dalhat M, Ogoina D, McCollum A, Disu Y, Mamadu I, Akinpelu A, Ahmad A, Burga J *et al*: **Outbreak of human monkeypox in Nigeria in 2017–18: a clinical and epidemiological report**. *The Lancet Infectious Diseases* 2019, **19**(8):872-879.

41. Guagliardo SAJ, Doshi RH, Reynolds MG, Dzabatou-Babeaux A, Ndakala N, Moses C, McCollum AM, Petersen BW: **Do Monkeypox Exposures Vary by Ethnicity? Comparison of Aka and Bantu Suspected Monkeypox Cases**. *The American journal of tropical medicine and hygiene* 2020, **102**(1):202-205.

42. Yinka-Ogunleye A, Aruna O, Ogoina D, Aworabhi N, Eteng W, Badaru S, Mohammed A, Agenyi J, Etebu EN, Numbere T-W *et al*: **Reemergence of Human Monkeypox in Nigeria, 2017**. *Emerging infectious diseases* 2018, **24**(6):1149-1151.

43. Hoff NA, Morier DS, Kisalu NK, Johnston SC, Doshi RH, Hensley LE, Okitolonda-Wemakoy E, Muyembe-Tamfum JJ, Lloyd-Smith JO, Rimoin AW: **Varicella Coinfection in Patients with Active Monkeypox in the Democratic Republic of the Congo**. *Ecohealth* 2017, **14**(3):564-574.

44. McCollum AM, Nakazawa Y, Ndongala GM, Pukuta E, Karhemere S, Lushima RS, Ilunga BK, Kabamba J, Wilkins K, Gao J *et al*: **Human Monkeypox in the Kivus, a Conflict Region of the Democratic Republic of the Congo**. *The American journal of tropical medicine and hygiene* 2015, **93**(4):718-721.

45. Formenty P, Muntasir MO, Damon I, Chowdhary V, Opoka ML, Monimart C, Mutasim EM, Manuguerra JC, Davidson WB, Karem KL *et al*: **Human monkeypox outbreak caused by novel virus belonging to Congo Basin clade, Sudan, 2005**. *Emerging infectious diseases* 2010, **16**(10):1539-1545.

46. Karem KL, Reynolds M, Hughes C, Braden Z, Nigam P, Crotty S, Glidewell J, Ahmed R, Amara R, Damon IK: **Monkeypox-induced immunity and failure of childhood smallpox vaccination to provide complete protection**. *Clinical and vaccine immunology : CVI* 2007, **14**(10):1318-1327.

47. Reynolds MG, Yorita KL, Kuehnert MJ, Davidson WB, Huhn GD, Holman RC, Damon IK: **Clinical manifestations of human monkeypox influenced by route of infection**. *The Journal of infectious diseases* 2006, **194**(6):773-780.

48. Reed KD, Melski JW, Graham MB, Regnery RL, Sotir MJ, Wegner MV, Kazmierczak JJ, Stratman EJ, Li Y, Fairley JA *et al*: **The detection of monkeypox in humans in the Western Hemisphere**. *N Engl J Med* 2004, **350**(4):342-350.

49. Centers for Disease C, Prevention: **Human monkeypox--Kasai Oriental, Zaire, 1996-1997**. *MMWR Morbidity and mortality weekly report* 1997, **46**(14):304-307.

50. Jezek Z, Grab B, Szczeniowski M, Paluku KM, Mutombo M: **Clinico-epidemiological features of monkeypox patients with an animal or human source of infection**. *Bull World Health Organ* 1988, **66**(4):459-464.

51. Jezek Z, Szczeniowski M, Paluku KM, Mutombo M: **Human monkeypox: clinical features of 282 patients**. *The Journal of infectious diseases* 1987, **156**(2):293-298.

52. Jezek Z, Arita I, Mutombo M: **Four generations of probable person-to-person transmission of human monkeypox**. *American Journal of Epidemiology* 1986, **123**(6):1004-1012.

53. Foster SO, Brink EW, Hutchins DL, Pifer JM, Lourie B, Moser CR, Cummings EC, Kuteyi OE, Eke RE, Titus JB *et al*: **Human monkeypox**. *Bull World Health Organ* 1972, **46**(5):569-576.

54. Learned LA, Reynolds MG, Wassa Wassa D, Li Y, Olson VA, Karem K, Stempora LL, Braden ZH, Kline R, Likos A *et al*: **Extended interhuman transmission of monkeypox in a hospital community in the Republic of the Congo, 2003**. *American Journal of Tropical Medicine and Hygiene* 2005, **73**(2):428-434.

55. Hobson G, Adamson J, Adler H, Firth R, Gould S, Houlihan C, Johnson C, Porter D, Rampling T, Ratcliffe L *et al*: **Family cluster of three cases of monkeypox imported from Nigeria to the United Kingdom, May 2021**. *Euro Surveill* 2021, **26**(32).

56. Minhaj FS, Ogale YP, Whitehill F, Schultz J, Foote M, Davidson W, Hughes CM, Wilkins K, Bachmann L, Chatelain R *et al*: **Monkeypox Outbreak - Nine States, May 2022**. *MMWR Morbidity and mortality weekly report* 2022, **71**(23):764-769.

57. Antinori A, Mazzotta V, Vita S, Carletti F, Tacconi D, Lapini LE, D'Abramo A, Cicalini S, Lapa D, Pittalis S *et al*: **Epidemiological, clinical and virological characteristics of four cases of monkeypox support transmission through sexual contact, Italy, May 2022**. *Euro surveillance : bulletin Europeen sur les maladies transmissibles = European communicable disease bulletin* 2022, **27**(22).

58. Davido B, D'Anglejan E, Jourdan J, Robinault A, Davido G: **Monkeypox 2022 outbreak: cases with exclusive genital lesions**. *Journal of travel medicine* 2022.

59. Dumont C, Irenge LM, Magazani EK, Garin D, Muyembe JJT, Bentahir M, Gala JL: **Simple technique for in field samples collection in the cases of skin rash illness and subsequent PCR detection of orthopoxviruses and varicella zoster virus**. *PLoS ONE* 2014, **9**(5).

60. Rimoin AW, Kisalu N, Kebela-Ilunga B, Mukaba T, Wright LL, Formenty P, Wolfe ND, Shongo RL, Tshioko F, Okitolonda E *et al*: **Endemic human monkeypox, democratic Republic of Congo, 2001-2004**. *Emerging infectious diseases* 2007, **13**(6):934-937.

61. Mandja BM, Brembilla A, Handschumacher P, Bompangue D, Gonzalez JP, Muyembe JJ, Mauny F: **Temporal and Spatial Dynamics of Monkeypox in Democratic Republic of Congo, 2000-2015**. *Ecohealth* 2019, **16**(3):476-487.

62. Eteng W-E, Mandra A, Doty J, Yinka-Ogunleye A, Aruna S, Reynolds MG, McCollum AM, Davidson W, Wilkins K, Saleh M *et al*: **Notes from the Field: Responding to an Outbreak of Monkeypox Using the One Health Approach - Nigeria, 2017-2018**. *MMWR Morbidity and mortality weekly report* 2018, **67**(37):1040-1041.

63. Doshi RH, Guagliardo SAJ, Dzabatou-Babeaux A, Likouayoulou C, Ndakala N, Moses C, Olson V, McCollum AM, Petersen BW: **Strengthening of Surveillance during Monkeypox Outbreak, Republic of the Congo, 2017**. *Emerging infectious diseases* 2018, **24**(6):1158-1160.

64. Hoff N, Ilunga BK, Shongo R, Muyembe JJ, Mossoko M, Okitolonda E, Rimoin AW: **Human monkeypox disease surveillance and time trends in The Democratic Republic of Congo, 2001-2013**. *American Journal of Tropical Medicine and Hygiene* 2014, **91**(5):339.

65. Likos AM, Sammons SA, Olson VA, Frace AM, Li Y, Olsen-Rasmussen M, Davidson W, Galloway R, Khristova ML, Reynolds MG *et al*: **A tale of two clades: monkeypox viruses**. *The Journal of general virology* 2005, **86**(Pt 10):2661-2672.

66. **From the Centers for Disease Control and Prevention. Human monkeypox--Kasai Oriental, Democratic Republic of Congo, February 1996-October 1997**. *Jama* 1998, **279**(3):189-190.

67. Mwanbal PT, Tshioko KF, Moudi A, Mukinda V, Mwema GN, Messinger D, Okito L, Barakymfyte D, Malfait P, Pebody R *et al*: **Human monkeypox in Kasai Oriental, Zaire (1996-1997)**. *Euro Surveill* 1997, **2**(5):33-35.

68. Mukinda VB, Mwema G, Kilundu M, Heymann DL, Khan AS, Esposito JJ: **Re-emergence of human monkeypox in Zaire in 1996. Monkeypox Epidemiologic Working Group**. *Lancet (London, England)* 1997, **349**(9063):1449-1450.

69. **Multi-country monkeypox outbreak** [<https://www.who.int/emergencies/disease-outbreak-news/item/2022-DON393>]

70. **Monkeypox** [<https://africacdc.org/disease/monkeypox/>]

71. **An Update of Monkeypox Outbreak in Nigeria** [<https://ncdc.gov.ng/diseases/sitreps/?cat=8&name=An%20Update%20of%20Monkeypox%20Outbreak%20in%20Nigeria>]

72. Meyer A, Esposito JJ, Gras F, Kolakowski T, Fatras M, Muller G: **[First appearance of monkey pox in human beings in Gabon]**. *Medecine tropicale : revue du Corps de sante colonial* 1991, **51**(1):53-57.

73. Vaughan A, Aarons E, Astbury J, Brooks T, Chand M, Flegg P, Hardman A, Harper N, Jarvis R, Mawdsley S *et al*: **Human-to-Human Transmission of Monkeypox Virus, United Kingdom, October 2018**. *Emerging infectious diseases* 2020, **26**(4):782-785.

74. Silenou BC, Tom-Aba D, Adeoye O, Arinze CC, Oyiri F, Suleman AK, Yinka-Ogunleye A, Dörrbecker J, Ihekweazu C, Krause G: **Use of Surveillance Outbreak Response Management and Analysis System for Human Monkeypox Outbreak, Nigeria, 2017-2019**. *Emerging infectious diseases* 2020, **26**(2):345-349.

75. McMullen CL, Mulembekani P, Hoff NA, Doshi RH, Mukadi P, Shongo R, Kebela-Illunga B, Okitolonda E, Muyembe JJ, Rimoin AW: **Human monkeypox transmission dynamics thirty years after smallpox eradication in the Sankuru district, democratic republic of Congo**. *American Journal of Tropical Medicine and Hygiene* 2015, **93**(4):341.

76. Jezek Z, Grab B, Szczeniowski MV, Paluku KM, Mutombo M: **Human monkeypox: secondary attack rates**. *Bull World Health Organ* 1988, **66**(4):465-470.

77. Fine PE, Jezek Z, Grab B, Dixon H: **The transmission potential of monkeypox virus in human populations**. *International journal of epidemiology* 1988, **17**(3):643-650.

78. Jezek Z, Marennikova SS, Mutumbo M: **Human monkeypox: A study of 2,510 contacts of 214 patients**. *Journal of Infectious Diseases* 1986, **154**(4):551-555.

79. Petersen BW, Kabamba J, McCollum AM, Lushima RS, Wemakoy EO, Muyembe Tamfum JJ, Nguete B, Hughes CM, Monroe BP, Reynolds MG: **Vaccinating against monkeypox in the Democratic Republic of the Congo**. *Antiviral research* 2019, **162**:171-177.

80. Kugelman JR, Johnston SC, Mulembakani PM, Kisalu N, Lee MS, Koroleva G, McCarthy SE, Gestole MC, Wolfe ND, Fair JN *et al*: **Genomic variability of monkeypox virus among humans, Democratic Republic of the Congo**. *Emerging infectious diseases* 2014, **20**(2):232-239.

81. Hoff N, Mulembakani PM, Johnston SC, Kisalu NK, Muyembe JJ, Hensley LE, Okitolonda E, Rimoin AW: **Risk factors associated with human monkeypox in the democratic republic of Congo**. *American Journal of Tropical Medicine and Hygiene* 2014, **91**(5):199-200.

82. Jezek Z, Grab B, Paluku KM, Szczeniowski MV: **Human monkeypox: disease pattern, incidence and attack rates in a rural area of northern Zaire**. *Tropical and geographical medicine* 1988, **40**(2):73-83.

83. Vaughan A, Aarons E, Astbury J, Balasegaram S, Beadsworth M, Beck CR, Chand M, O'Connor C, Dunning J, Ghebrehewet S *et al*: **Two cases of monkeypox imported to the United Kingdom, September 2018**. *Euro Surveill* 2018, **23**(38).

84. Mahase E: **Seven monkeypox cases are confirmed in England**. *BMJ (Clinical research ed)* 2022, **377**:o1239.

85. **Emergence of monkeypox in West Africa and Central Africa, 1970–2017**. *Releve epidemiologique hebdomadaire* 2018, **93**(11):125-132.

86. Ladnyj ID, Ziegler P, Kima E: **A human infection caused by monkeypox virus in Basankusu Territory, Democratic Republic of the Congo**. *Bull World Health Organ* 1972, **46**(5):593-597.
